# Capture of Mouse and Human Stem Cells with Features of Formative Pluripotency

**DOI:** 10.1016/j.stem.2021.11.002

**Published:** 2021-12-02

**Authors:** Masaki Kinoshita, Michael Barber, William Mansfield, Yingzhi Cui, Daniel Spindlow, Giuliano Giuseppe Stirparo, Sabine Dietmann, Jennifer Nichols, Austin Smith

(Cell Stem Cell *28*, 1–19.e1–e8, March 4, 2021)

The accession number for RNA-seq and ATAC-seq data listed in the Key Resources Table was incorrect due to a typographical error. The correct accession number is “GEO: GSE131556,” not GSE131566. This error has been corrected in the online version of the paper. The authors apologize for this error and any inconvenience caused.

