# Supplementary material for: Capture of Mouse and Human Stem Cells with Features of Formative Pluripotency
Source: Cell Stem Cell. 2021 Mar 4;28(3):453–471.e8. doi: 10.1016/j.stem.2020.11.005 (PMC7939546; doi:10.1016/j.stem.2020.11.005)
Supplement: Document S1. Figures S1–S7 [file mmc1.pdf]

Cell Stem Cell, Volume *28*

Supplemental Information

Capture of Mouse and Human Stem Cells with Features of Formative Pluripotency

Masaki Kinoshita, Michael Barber, William Mansfield, Yingzhi Cui, Daniel Spindlow, Giuliano Giuseppe Stirparo, Sabine Dietmann, Jennifer Nichols, and Austin Smith

Figure S1 (Related to Figure 1)

A

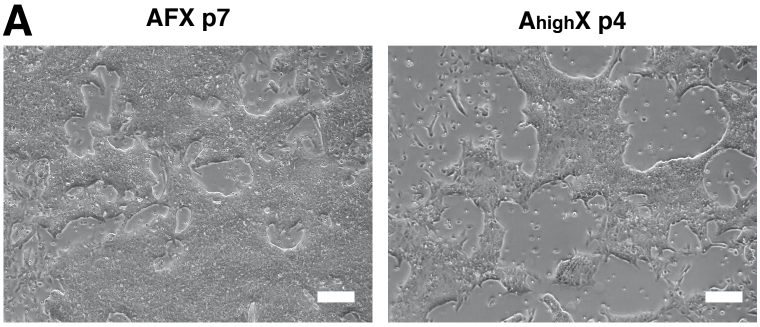

B

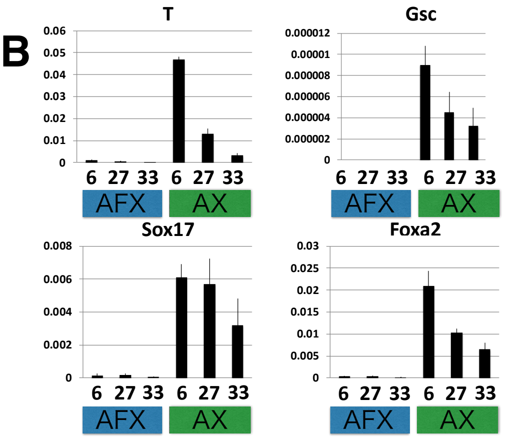

C

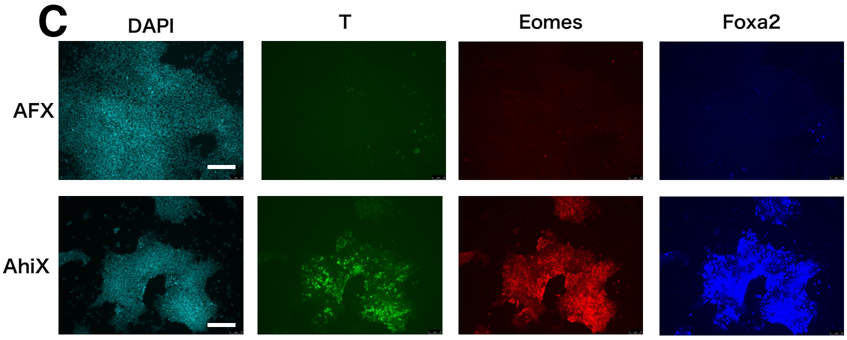

D

Concentration of activin	No of E5.5 embryos	No of established lines (> p10)	Success rate
1ng	8	0	0.0%
1.5ng	8	1	12.5%
2ng	11	3	27.3%
2.5ng	4	0	0.0%
3ng	11	4	36.4%

E

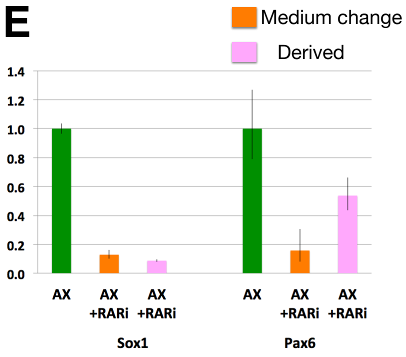

F

	Number of E5.5 embryos	Lines established	Efficiency
AloX	8	4	50%
AloX +RARi	4	4	100%

G

Line	Percentage of diploid (=40) cells	Passage number
5a6	75.0 % (24/32)	19
NBRA3.2	87.1 % (27/31)	22
5ar1	64.0 % (16/25)	21
5ar2	83.3% (20/24)	26

H

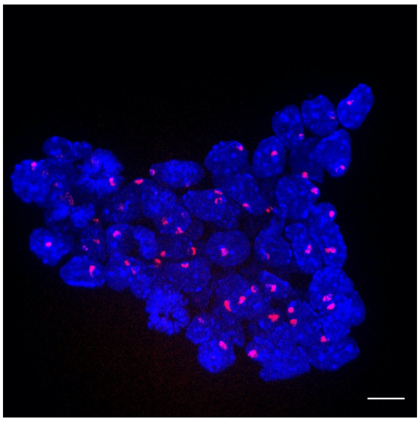

I

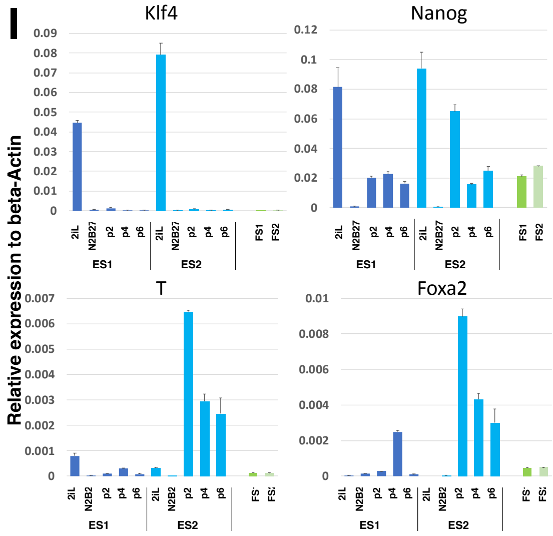

J

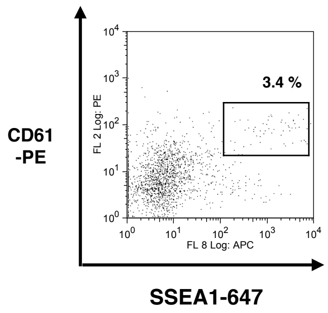

K

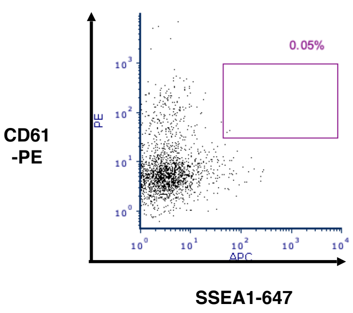

L

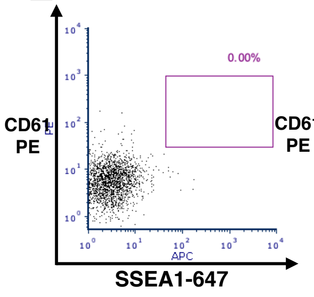

M

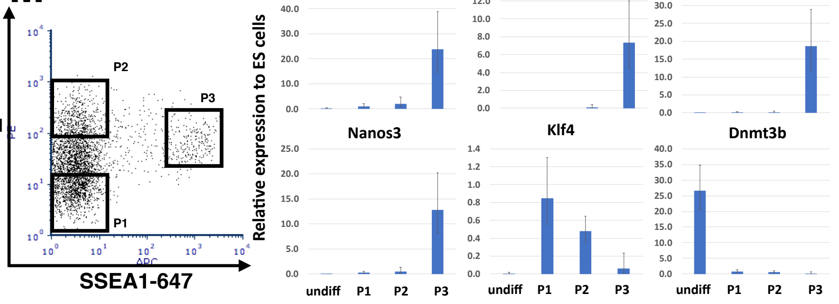

N

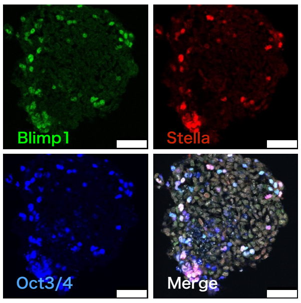

Figure S2 (Related to Figure 2)

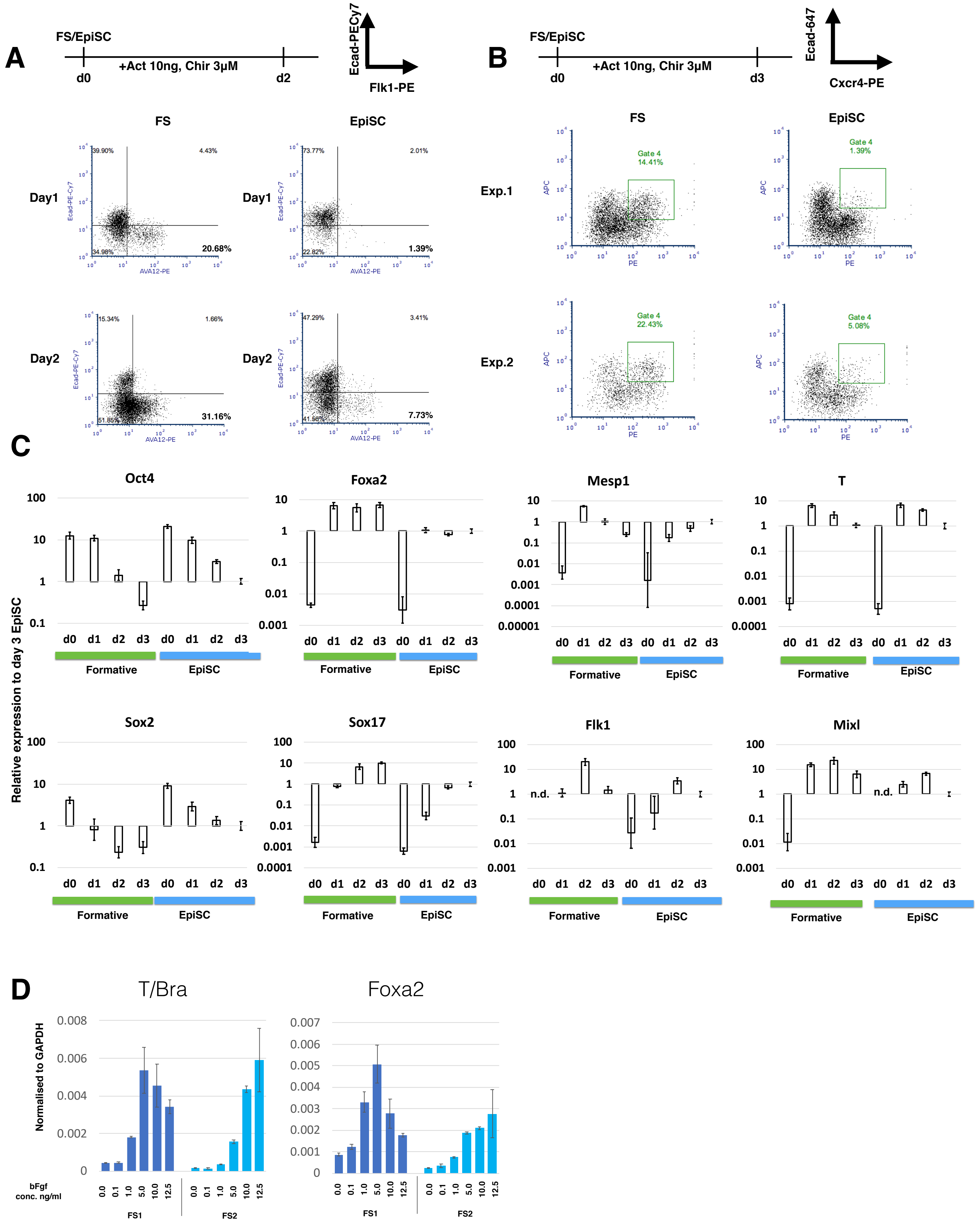

Figure S3 (Related to Figure 3)

A B C

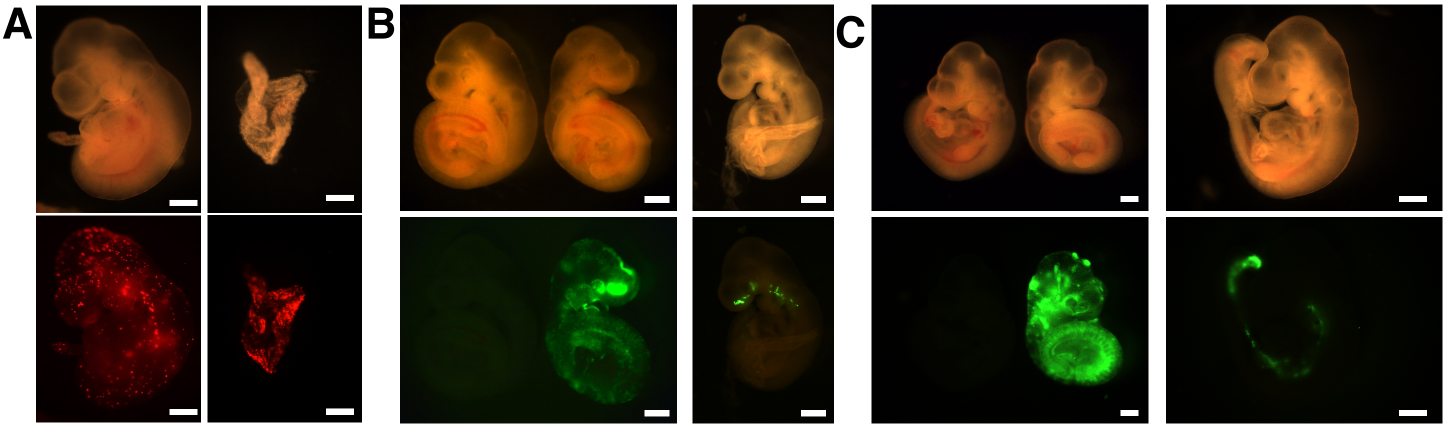

D

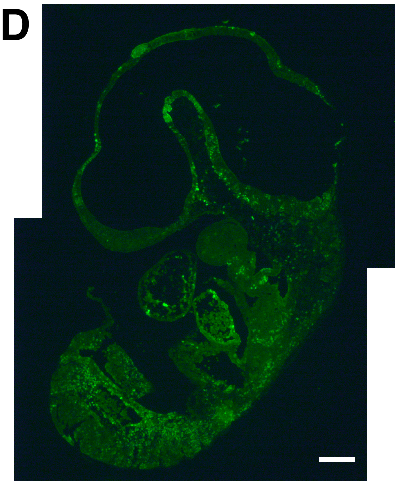

E

Line	Embryos recovered	Widespread Contribution	Localised contribution	Yolk sac contribution	Chimaera rate
NBRA3.2	48	8	11	1*	39.6%
5a6	20	1	2	1	15.0%
5ar1	17	1	3	Not Investigated	23.5%

F

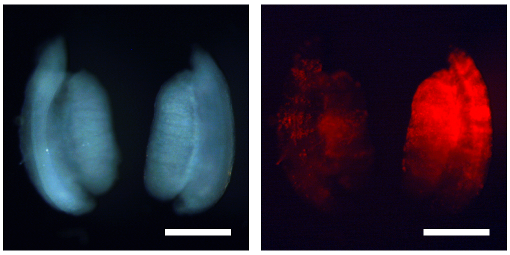

G

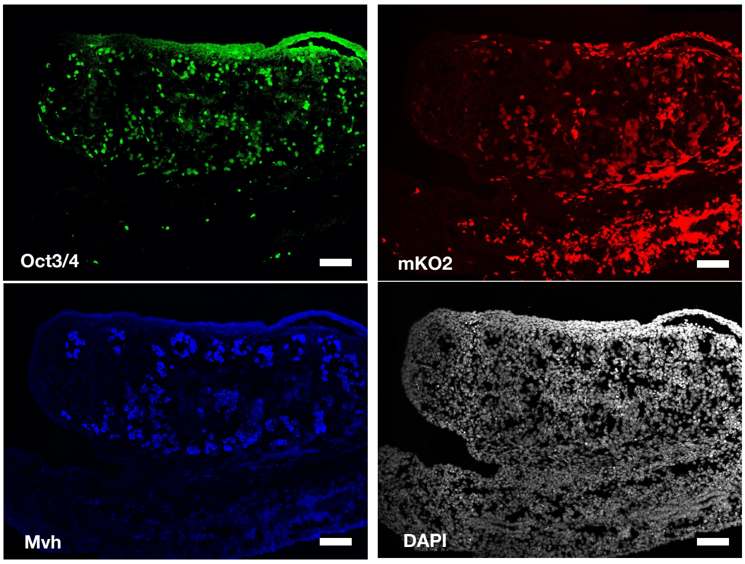

H
E6.0 Epiblast Chimaeras

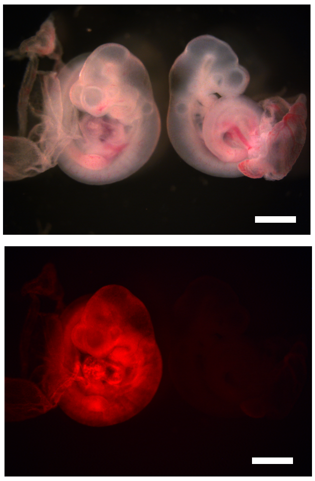

I
E5.5 Epiblast Chimaera

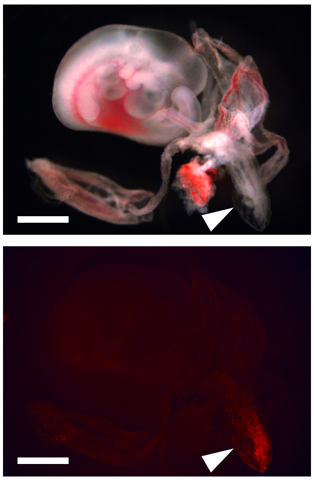

J
E5.5 Epiblast Chimaera

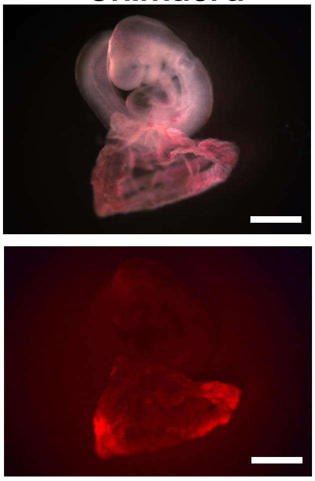

K
K'

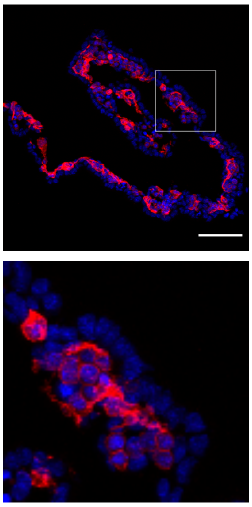

L

Donor stage	Embryos recovered	Widespread Contribution	Localised contribution	contribution yolk sac and/or amnion	Chimaera rate
E5.5	44	2	5	7	16.0%
E6.0	47	1	3	4	8.5%
E6.5	29	0	0	0	0.0%

Figure S4 (Related to Figure 4)

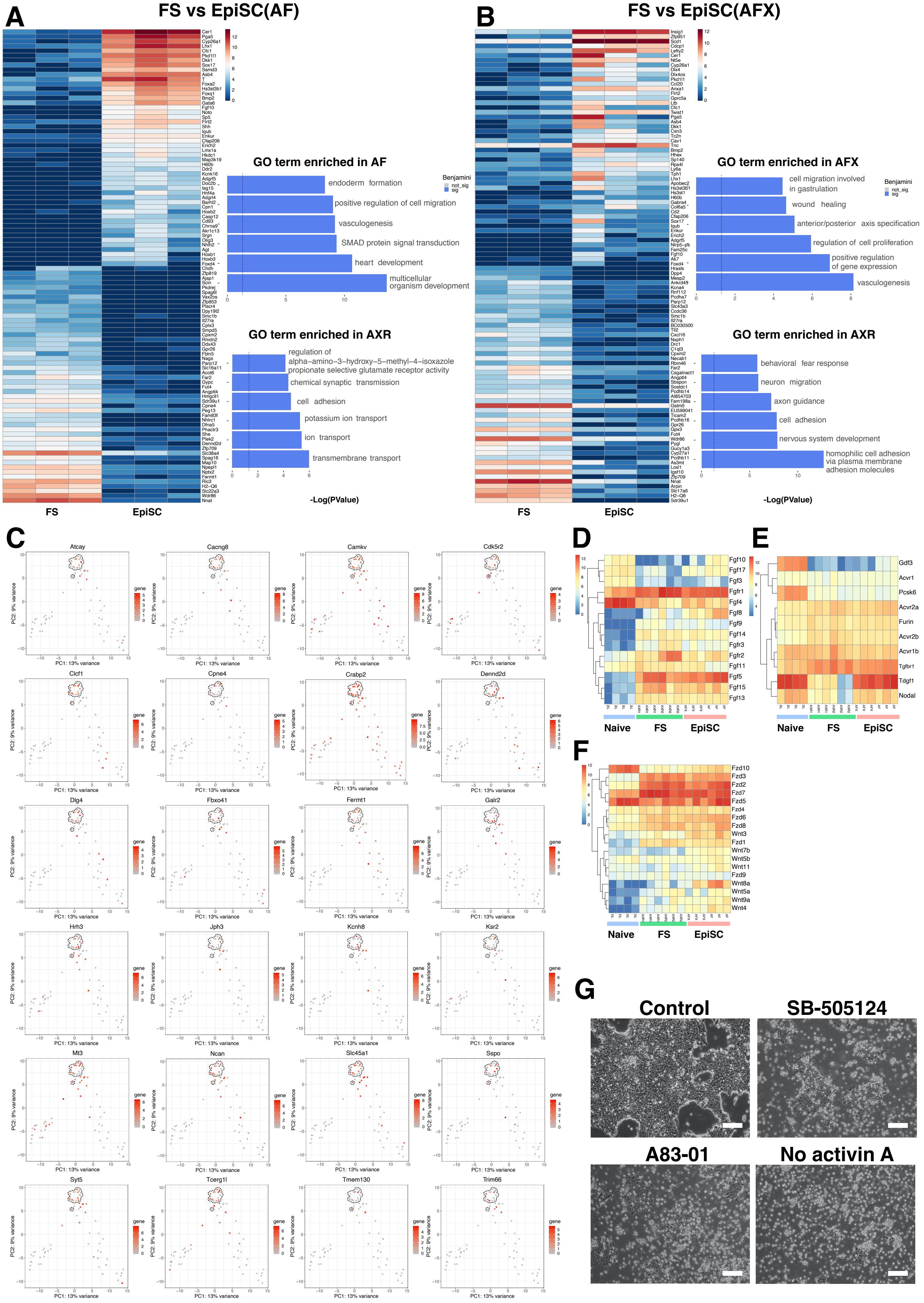

Figure S5 (Related to Figure 5)

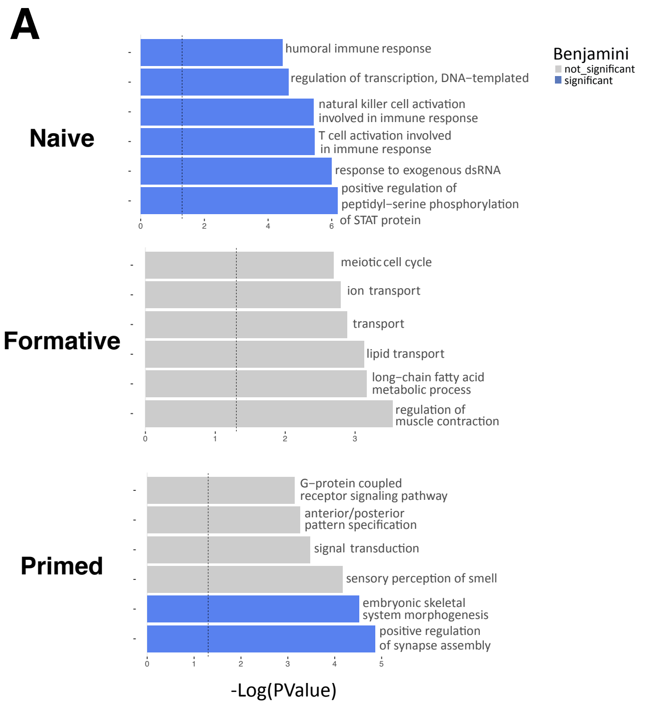

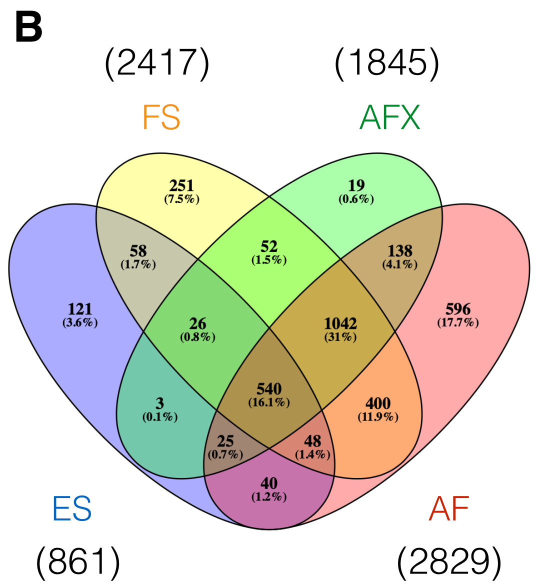

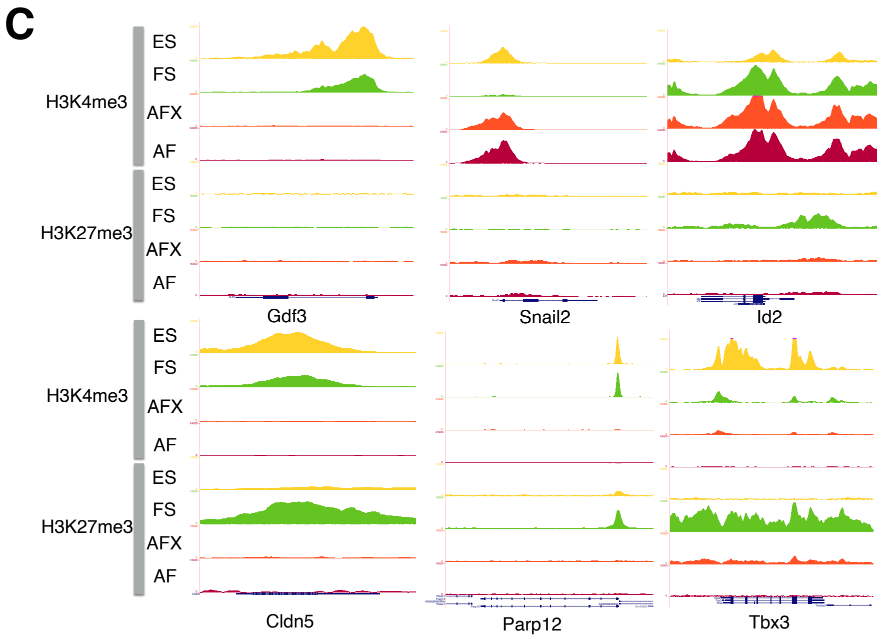

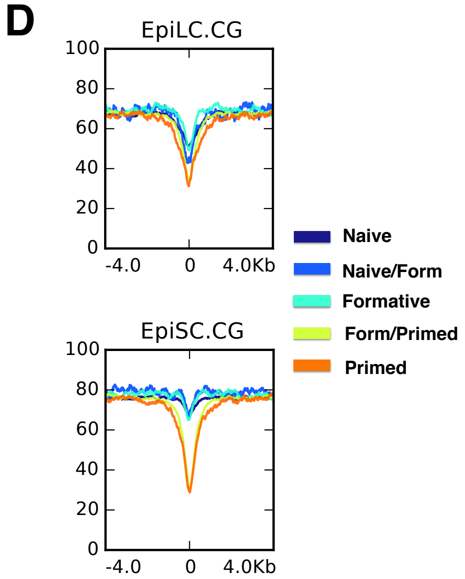

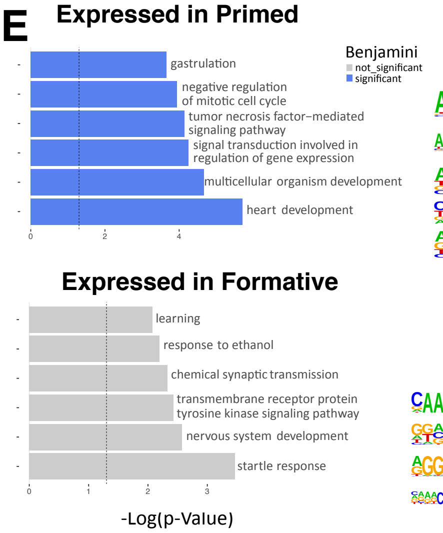

F

Common binding motif

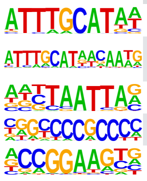

TF	Naive P value	Naive q.value (Benjamini)	Formative P value	Formative q.value (Benjamini)	Primed P value	Primed q.value (Benjamini)
Oct4 (POUHomeobox)	1E-46	0	1E-20	0	1E-37	0
Oct4-Sox2-TCF-NANOG (POU Homeobox HMG)	1E-44	0	1E-06	0	1E-59	0
Lhx3 (Homeobox)	1E-09	0	1E-19	0	1E-04	0.0002
Sp2 (Zf)	1E-06	0	1E-64	0	1E-08	0
Etv4 (ETS)	1E-02	0.0282	1E-04	0.0002	1E-02	0.0214

Naive enriched motif

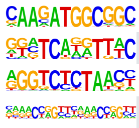

TF	P value	q.value (Benjamini)
YY1 (Zf)	1E-06	0
Six1 (Homeobox)	1.00E-04	5.00E-04
PRDM14 (Zf)	0.01	0.023
Tcfcp2l1 (CP2)	0.01	0.0468

Formative enriched motif

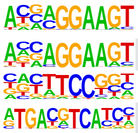

TF	P value	q.value (Benjamini)
ELF5 (ETS)	1.00E-10	0
ETS1 (ETS)	1.00E-08	0
Fli (ETS)	1E-08	0
c-Jun-CRE (bZIP)	1.00E-05	0

Primed enriched motif

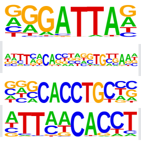

TF	P value	q.value (Benjamini)
GSC (Homeobox)	1.00E-10	0
Brachyury (T-box)	1.00E-08	0
Slug (Zf)	1E-08	0
Eomes (T-box)	1E-06	0

Figure S6 (Related to Figure 6)

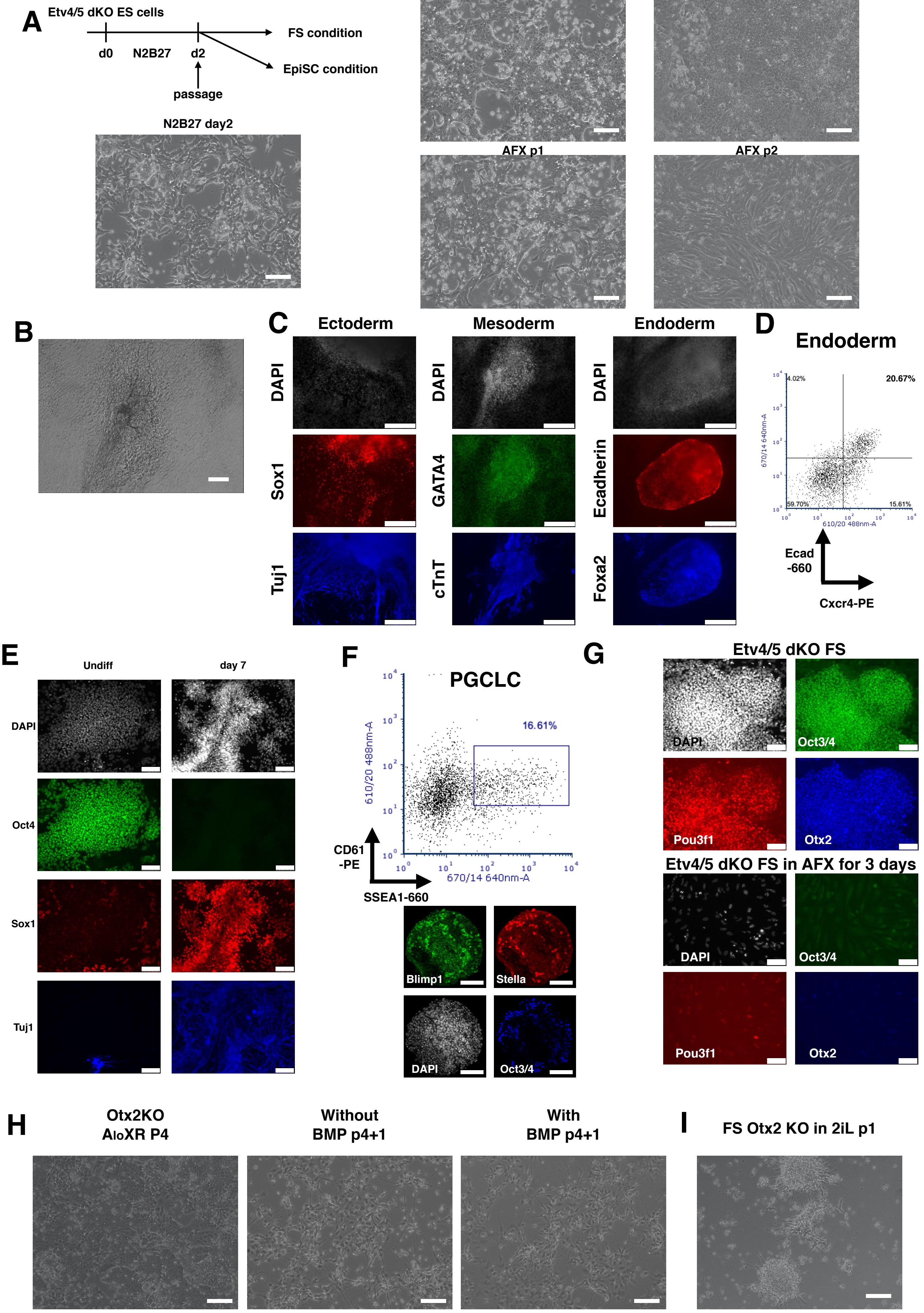

Figure S7 (Related to Figure 7)

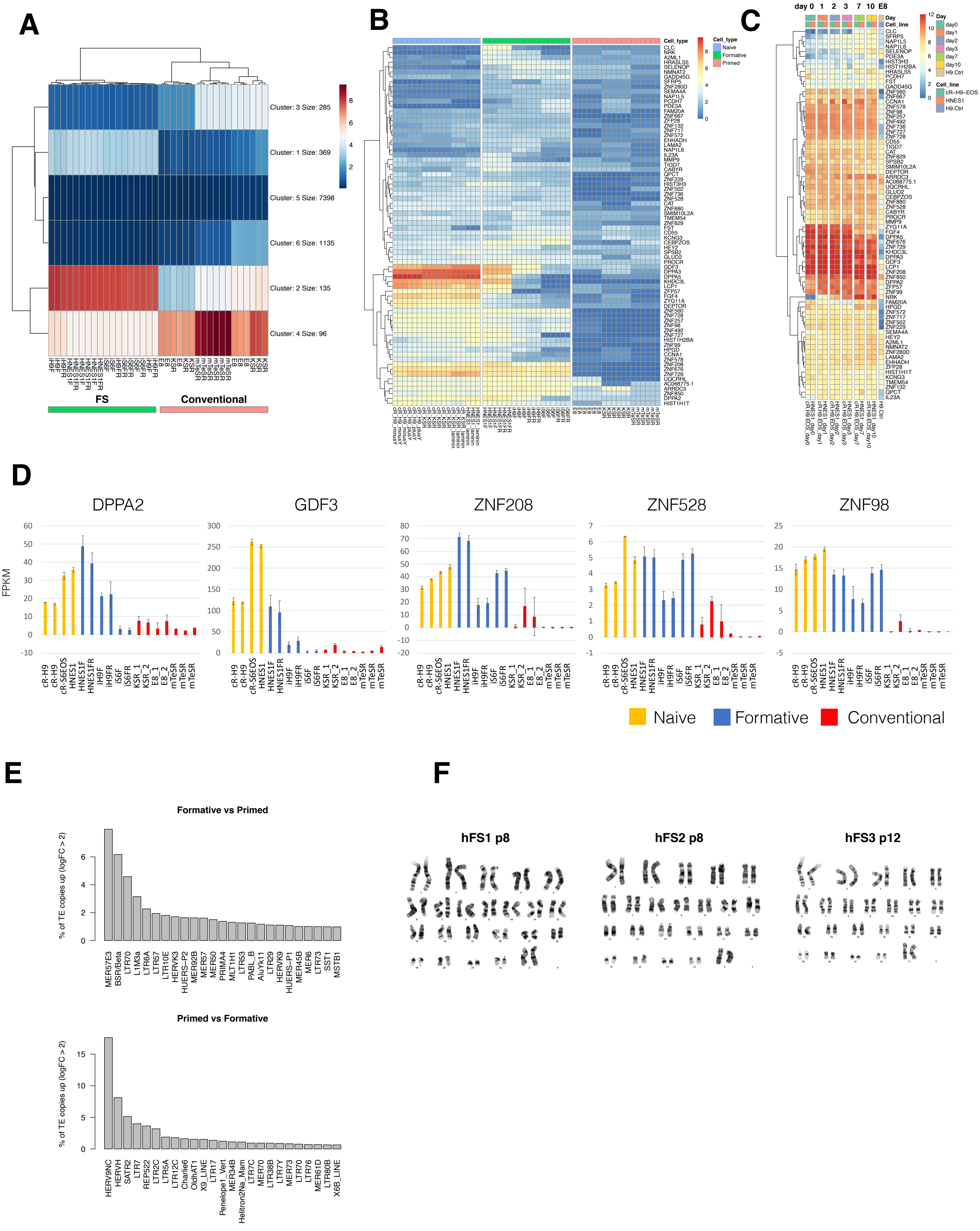

Supplemental Figure Legends

Figure S1. Derivation of stem cell lines from formative epiblast, Related to Figure 1
(A) Bright field image of E5.5 epiblast derived AFX and $A_{hi}X$ cultures. Scale bars, 200μm. (B) Gene expression analysis after FGF withdrawal. Three AFX cell lines (6, 27 and 33) were passaged without FGF and analysed by RT-qPCR. Error bars are S.D. from technical triplicates. (C) Immunostained images of early lineage marker expression in AFX and $A_{hi}X$ cells. Scale bars, 100μm. (D) Summary of derivation efficiency from E5.5 epiblasts in different concentrations of activin A. (E) RT-qPCR analysis of RAR inhibitor treated cells. $A_{lo}XR$ samples established in $A_{lo}X$ and transferred to $A_{lo}XR$ are in orange and a line derived in $A_{lo}XR$ in pink. Error bars, S.D. from technical triplicates. (F) Derivation efficiency in the absence of presence of RAR inhibitor. (G) Percentages of diploid cells for 4 FS cell lines. (H) Maximum projection of Z-stack slices of *Xist* RNA FISH images (red) in female FS cells. Nuclei were stained with DAPI (blue). Scale bar, 10μm. (I) Gene expression analysis by RT-qPCR during ES cell to FS cell conversion. Gene expression is relative to beta-actin. Error bars are S.D. from two technical replicates. (J) Flow cytometry analysis of day 4 PGCLC induction from $A_{lo}X$ FS cells. (K) Analysis of day 4 PGCLC induction from AFX EpiSCs. (L) Analysis of day 4 PGCLC induction from AFX EpiSCs adapted to culture in $A_{lo}XR$. (M) $A_{lo}XR$ cells sorted for SSEA1 and CD61 co-expression on day 6 of PGCLC induction (left) and subject to RT-qPCR analysis (right). Relative expression level to 2iL ES cells (=1) normalized to Tbp. Error bars represent S.D. from technical triplicates. (N) Immunostaining of $A_{lo}X$ cell-derived PGCLC. Scale bars, 50μm.

Figure S2. Lineage potency of FS cells and responsiveness to differentiation cues, Related to Figure 2
(A) Flow cytometry profiles of $Flk1^{+}Ecad^{-}$ mesodermal fraction of differentiated FS cells and EpiSCs at day 1 and day 2. (B) $Cxcr4^{+}Ecad^{+}$ endoderm fraction at day 3. Two experiments are shown. (C) RT-qPCR analysis after activin A and CH treatment for 3 days. AFX EpiSC samples at day 3 were set as 1, normalisation to 36B4 (Rplp0). Error bars represent SD from technical triplicates. n.d. not detected. (D) RT-qPCR analysis of T and Foxa2 expression 24 hours after indicated doses of Fgf2 were added into $A_{lo}XR$ culture. Error bars represent S.D. from technical duplicates.

Figure S3. Blastocyst chimaera contribution by FS cells and formative epiblast, Related to Figure 3
(A) Left, low contribution E9.5 chimaera produced from mKO2-labelled NBRA3.2 FS cells. Right, yolk sac contribution in one of the chimaeras in Fig. 3A. Scale bars, 500μm. (B) E9.5 chimaeras from GFP-labelled 5a6 FS cells. Contributions were widespread (left) or localised (right). Scale bars, 500μm. (C) E9.5 chimaeras from GFP-labelled 5ar1 FS cells. Scale bars, 500μm. (D) Sagittal section of embryo from C, left panel, with widespread contribution of GFP positive cells. Scale bar, 200μm. (E) Summary of FS cell chimaeras examined at E9.5. *Not all yolk sacs from chimaeric embryos were examined. (F) E12.5 chimaeric gonads generated from mKO2-labelled FS cells. Scale bars, 500μm. (G) Section of gonad from (F) stained with anti-Oct4 and anti-Mvh antibodies. Nuclei were stained with DAPI. (H-J). E9.5 chimaeras with contribution from E5.5 and E6.0 donor epiblast. Contributions were detected in the embryo proper and yolk sac (H), amnion (arrowhead) (I), yolk sac (J). Scale bars, 1mm. (K) Yolk sac section showing membrane-tdTomato positive cells in the inner layer of extraembryonic mesoderm. Nuclei were stained with DAPI (blue). Scale bar, 100μm. Magnified image from boxed region is shown as (K'). (L) Summary of post-implantation epiblast chimaeras.

Figure S4. Whole transcriptome analysis and nodal/activin pathway activity, Related to Figure 4

(A) Heatmap for top 50 differentially expressed genes (DEG) between FS cells and EpiSCs (AF). GO terms are shown (Benjamini value<0.05) for analysis of 200 DEG. (B) Heatmap for top 50 DEG between FS cells and EpiSCs (AFX). GO term analysis as in A (Benjamini value<0.05). (C) Example embryo gene expression profiles of FS cell enriched genes identified in Fig. 4B. E5.5 epiblast cells are highlighted by the dashed circle. (D) Heatmap of expression of Fgfs and Fgfrs. (E) Heatmap of Nodal pathway gene expression. (F) Heatmap of expression of Wnts and Fzd receptors. Colour scale in (D-F) is log2(normalised counts +1) from RNA-seq. (G) Cell morphologies after two days in indicated culture conditions: A_{lo}XR; 1μM A83-01 in A_{lo}XR; 5μM SB505124 in A_{lo}XR; without activin A in 2μM XAV939 and 1μM BMS493. Scale bars,100μm.

Figure S5. Chromatin landscape analysis, Related to Figure 5

(A) GO term enrichment for genes proximal to phase specific ATAC-seq sites. Bars in blue have a significant Benjamini value<0.05. (B) Enumeration of bivalent domains in each cell type. (C) Genome browser screenshots of differential histone modifications. Lower three examples show formative specific bivalency. (D) Methylation at ATAC peaks in EpiLCs and EpiSCs (original data from Zylicz et al., 2015). (E) Related to Fig. 5G. GO term analysis performed against significantly expressed genes in EpiSCs or FS cells. Bars in blue have a significant Benjamini value<0.05. (F) Transcription factor binding motifs and P-values enriched in phase specific ATAC sites.

Figure S6. Differential requirements for *Etv4/5* and *Otx2*, Related to Figure 6

(A) Schematic of ES cell differentiation to FS cells or EpiSCs and morphologies of *Etv4/5*dKO cells at day 2, P1 and P2. (B) Bright field image of contracting *Etv4/5*dKO differentiated cells. (C) Immunostaining of *Etv4/5*dKO FS cell EB outgrowth. Neuroectoderm stained with Sox1 (red) and Tuj1 (Blue), mesoderm with Gata4 (Green) and cTnT (blue), and endoderm with Ecadherin (red) and Foxa2 (Blue). DAPI stainings were shown in white. (D) Flow cytometry plot of endoderm differentiated *Etv4/5*dKO FS cells. (E) Immunostaining for Oct3/4 (green), Sox1 (red) and Tuj1 (Blue) after neural differentiation of *Etv4/5*dKO FS cells. (F) PGCLC induction from *Etv4/5*dKO FS cells analysed by flow cytometry for SSEA1-660 and CD61-PE and by immunostaining for Blimp1 (green), Stella (red) and Oct4 (blue). (G) Immunostaining of *Etv4/5*dKO FS cells in A_{lo}XR and after transfer to EpiSC culture (AFX) for three days. (H) *Otx2* KO cells passaged in A_{lo}XR with or without BMP. (I) Bright field image of *Otx2* KO FS cells re-plated in 2iL. Scale bars in (A), (B), (F), (H), (I) 100μm, (C) 250μm and (E), (G) 75μm.

Figure S7. Human FS-like cells established from naïve ES cells and embryos, related to Figure 7

(A) K-mean clustering of differential gene expression between human FS-like cells and conventional PSCs. (B) Gene expression heatmap for cluster 1 protein coding genes. (C) Expression heatmap of cluster 1 protein coding genes during naïve cell capacitation (data from Rostovskaya et al 2019). (D) Related to Figure 7I, FPKM values for additional selected naïve-formative specific genes. (E) Bar charts of differentially expressed TE families between formative and conventional hPSCs. (F) G-banded chromosomes from three independent human embryo derived FS-like cell lines.
