# Supplementary material for: Capture of Mouse and Human Stem Cells with Features of Formative Pluripotency
Source: Cell Stem Cell. 2021 Mar 4;28(3):453–471.e8. doi: 10.1016/j.stem.2020.11.005 (PMC7939546; doi:10.1016/j.stem.2020.11.005)
Supplement: Document S2. Article plus Supplemental Information [file mmc6.pdf]

Cell Stem Cell

Article

# Capture of Mouse and Human Stem Cells with Features of Formative Pluripotency

## Graphical Abstract

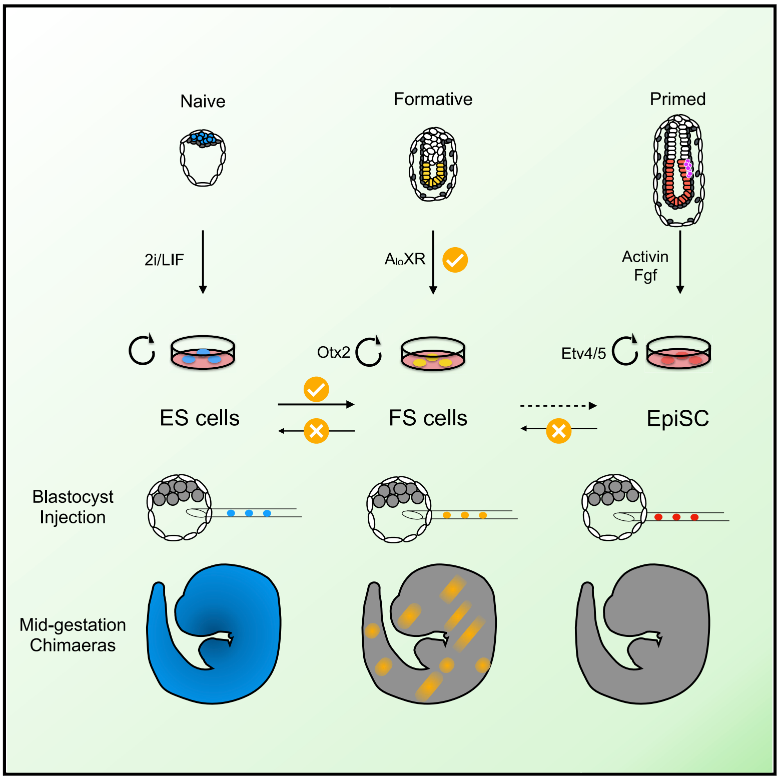


## Highlights

- Derivation of mouse formative pluripotent stem cells from pre-gastrulation epiblast
- Formative stem cells are competent for germline induction
- Formative stem cells can contribute to chimeras
- Candidate human FS cells derived using similar culture conditions to mouse


## Authors

Masaki Kinoshita, Michael Barber, William Mansfield, ..., Sabine Dietmann, Jennifer Nichols, Austin Smith

## Correspondence

mk704@cam.ac.uk (M.K.),
austin.smith@exeter.ac.uk (A.S.)



## In Brief

Three stages of pluripotency have been proposed: naive, formative, and primed. Kinoshita and colleagues derived stem cells with properties anticipated for formative pluripotency by culturing mouse epiblast under conditions of low growth factor stimulation. Application to human embryos resulted in propagation of similar stem cells.


Kinoshita et al., 2021, Cell Stem Cell 28, 453–471
March 4, 2021 © 2020 The Authors. Published by Elsevier Inc.
https://doi.org/10.1016/j.stem.2020.11.005

Cell Stem Cell

CellPress
OPEN ACCESS

Article

# Capture of Mouse and Human Stem Cells with Features of Formative Pluripotency

Masaki Kinoshita,[1,*] Michael Barber,[1,5] William Mansfield,[1] Yingzhi Cui,[2] Daniel Spindlow,[1,4] Giuliano Giuseppe Stirparo,[1,4] Sabine Dietmann,[1,6] Jennifer Nichols,[1,3] and Austin Smith[1,2,4,7,*]

[1]Wellcome-MRC Cambridge Stem Cell Institute, Jeffrey Cheah Biomedical Centre, University of Cambridge, Cambridge CB2 0AW, UK
[2]Department of Biochemistry, University of Cambridge, Cambridge CB2 1GA, UK
[3]Department of Physiology, Development and Neuroscience, University of Cambridge, Cambridge CB2 3DY, UK
[4]Living Systems Institute, University of Exeter, Exeter EX4 4QD, UK
[5]Present address: MRC Human Genetics Unit, University of Edinburgh, Edinburgh EH4 2XU, Scotland, UK
[6]Present address: Department of Developmental Biology and Division of Nephrology, Washington University School of Medicine, St Louis, MO, USA
[7]Lead Contact
*Correspondence: mk704@cam.ac.uk (M.K.), austin.smith@exeter.ac.uk (A.S.)
https://doi.org/10.1016/j.stem.2020.11.005

## SUMMARY

Pluripotent cells emerge as a naive founder population in the blastocyst, acquire capacity for germline and soma formation, and then undergo lineage priming. Mouse embryonic stem cells (ESCs) and epiblast-derived stem cells (EpiSCs) represent the initial naive and final primed phases of pluripotency, respectively. Here, we investigate the intermediate formative stage. Using minimal exposure to specification cues, we derive stem cells from formative mouse epiblast. Unlike ESCs or EpiSCs, formative stem (FS) cells respond directly to germ cell induction. They colonize somatic tissues and germline in chimeras. Whole-transcriptome analyses show similarity to pre-gastrulation formative epiblast. Signal responsiveness and chromatin accessibility features reflect lineage capacitation. Furthermore, FS cells show distinct transcription factor dependencies, relying critically on Otx2. Finally, FS cell culture conditions applied to human naive cells or embryos support expansion of similar stem cells, consistent with a conserved staging post on the trajectory of mammalian pluripotency.

## INTRODUCTION

Mouse embryonic stem cells (ESCs) correspond to naive epiblast, a transient population in the pre-implantation embryo (Hackett and Surani, 2014; Smith, 2017). As the embryo implants, naive pluripotency transcription factors are downregulated and their ability to form ESCs is lost, while transcription factors such as Otx2 and Pou3f1 are upregulated together with *de novo* methyltransferases Dnmt3a and Dnmt3b (Acampora et al., 2016; Auclair et al., 2014; Boroviak et al., 2014, 2015; Brook and Gardner, 1997). After this transition, epiblast cells manifest competence for primordial germ cell (PGC) induction (Ohinata et al., 2009). Subsequently, the epiblast becomes progressively regionally fated and molecularly diverse (Beddington and Robertson, 1998; Cheng et al., 2019; Lawson et al., 1991; Peng et al., 2016, 2019). These events are mirrored by ESCs entering into differentiation (Hayashi et al., 2011; Kalkan et al., 2017; Mulas et al., 2017). We hypothesize that exit from naive pluripotency heralds a formative conversion that instates competence for both soma and germline induction (Kalkan and Smith, 2014; Kinoshita and Smith, 2018; Smith, 2017).

Cultures termed epiblast-derived stem cells (EpiSCs) have been obtained by exposure of embryo explants to fibroblast growth factor (FGF) and activin (Brons et al., 2007; Guo et al., 2009; Tesar et al., 2007). EpiSCs can be derived from all stages of epiblast (Kojima et al., 2014; Najm et al., 2011; Osorno et al., 2012) but invariably converge on mid-gastrula stage phenotypes, generally displaying transcriptome relatedness to primed epiblast of the anterior primitive streak (Kojima et al., 2014; Tsakiridis et al., 2014). Thus, culture of epiblast in relatively high levels of FGF (12.5 ng/ml) and activin (20 ng/ml) results in the propagation of a form of primed pluripotency, which is likely dictated by these strong growth factor signals.

Notably, EpiSCs are refractory to PGC induction, unlike embryonic day 5.5 (E5.5)–6.5 epiblast. (Hayashi et al., 2011; Murakami et al., 2016; Ohinata et al., 2009). Naive ESCs are also unresponsive to germ cell inductive stimuli, unless they are transitioned for 24–48 h into a population termed epiblast-like cells (EpiLCs) (Hayashi et al., 2011; Nakaki et al., 2013). EpiLCs are molecularly as well as functionally distinct from both naive ESCs and EpiSCs (Buecker et al., 2014; Hayashi et al., 2011; Kalkan et al., 2017; Smith, 2017). They are enriched in formative phase cells related to pre-streak epiblast but are heterogeneous and persist only transiently (Hayashi et al., 2011).

Here, we invested in an effort to capture and propagate stem cells representative of mouse post-implantation epiblast between E5.5–E6.0, when the formative transition is expected to be completed but epiblast cells remain mostly unspecified.


This is an open access article under the CC BY license (http://creativecommons.org/licenses/by/4.0/).

## RESULTS

### Derivation of Stem Cell Cultures from Mouse Formative Epiblast

We hypothesized that shielding formative epiblast cells from lineage-inductive stimuli while maintaining autocrine growth and survival signals may stall developmental progression but sustain propagation. Nodal, FGF4, and FGF5 are broadly expressed in the early post-implantation epiblast (Haub and Goldfarb, 1991; Mesnard et al., 2006; Niswander and Martin, 1992; Varlet et al., 1997) and promote lineage capacitation in mouse ESCs (Hayashi et al., 2011; Kunath et al., 2007; Mulas et al., 2017; Stavridis et al., 2007). They are therefore candidates for supporting formative pluripotency. However, together with Wnt3 and bone morphogenetic proteins (BMPs), these growth factors also drive specification in the gastrula (Liu et al., 1999; Winnier et al., 1995).

We speculated that in a context of Wnt inhibition and absence of BMP, moderate stimulation of FGF and Nodal pathways may sustain a formative population. We used the Tankyrase inhibitor XAV939 to block canonical Wnt signaling and excluded undefined components such as feeders, serum, knockout serum replacement (KSR), or matrigel. Autocrine Nodal is known to be downregulated *in vitro* in the absence of extraembryonic tissues (Guzman-Ayala et al., 2004); therefore, we added activin A (20 ng/ml) as a substitute. E5.5 epiblasts were isolated by microdissection and plated intact in individual fibronectin-coated 4-well plates in N2B27 medium under 5% $O_2$ conditions (Figure 1A). After 5–6 days, explants were treated with Accutase for 5–10 s and then gently detached, fragmented into small clumps, and seeded into fresh 4-well plates. With or without added FGF, colonies of tightly packed epithelioid cells grew that could be passaged further and expanded into continuous cell lines (Figures 1A and S1A). In the absence of FGF, we observed an appreciably higher expression of primitive streak markers Brachyury, FoxA2, Eomes, and Gsc (Figures S1B and S1C). Nodal/activin signaling is known to stimulate these genes (Brennan et al., 2001; Conlon et al., 1994; Takenaga et al., 2007). We titrated activin and found that continuous cultures could still be established in the absence of FGF (Figures 1B and S1D). In low activin (3 ng/ml) plus XAV939 ($A_{lo}X$), we obtained cell lines that could be propagated for more than 20 passages (Figures 1B and S1D; Video S1).

Cell lines derived in $A_{lo}X$ expressed *Otx2*, consistent with post-implantation identity but showed no expression of *T* and minimal *FoxA2* (Figures 1C and 1D). They displayed similar levels of *Pou5f1 (Oct4)* mRNA to EpiSCs, slightly higher *Sox2*, and lower *Nanog*. (Figure 1C). Upon embryoid body formation and outgrowth, we detected germ layer markers indicating multi-lineage differentiation (Figure 1E).

These observations suggest that in the absence of other stimuli, limited stimulation of the Nodal/activin pathway combined with autocrine FGF activity may suspend cells in the formative phase of pluripotency.

### Stem Cell Propagation Is Facilitated by Retinoic Acid Receptor Inhibition and Requires Nodal Pathway Activity

During establishment and expansion in $A_{lo}X$, we observed sporadic expression of neural lineage markers and appearance of neuronal morphologies. On occasion, differentiation was extensive and led to loss of cultures. We speculated that retinoids might be acting as neural inductive stimuli (Bain et al., 1995; Stavridis et al., 2010). We therefore applied a pan-retinoic acid receptor inverse agonist (RARi; BMS 493; 1.0 μM) (Figure S1E). Supplementation of $A_{lo}X$ with RARi, henceforth $A_{lo}XR$, resulted in improved derivation efficiency (Figure S1F), reduced ectopic expression of neural specification factors Sox1 and Pax6 (Figure S1E), and stabilized long-term cultures. Using $A_{lo}XR$, we established nine cell lines from embryos of two different strains, namely, 129 and CD1. These lines were all passaged more than 10 times (30 generations) with no indication of crisis or senescence. Established cultures expanded slightly slower than EpiSCs and similar to ESCs, with routine passaging every 2–3 days at a split ratio of 1/10 to 1/15. Chromosome counts showed a majority of diploid cells even at later passages (Figure S1G). Cells were routinely passaged by mild dissociation into small clumps. Survival was poor after dissociation to single cells, but addition of Rho-associated kinase inhibitor (ROCKi) (Watanabe et al., 2007) enabled reliable clonal expansion.

Using fluorescent *in situ* hybridization, we detected a prominent cloud of Xist expression in nuclei of a female line (Figure S1H). Upregulation of Xist is indicative of initiation of X chromosome inactivation, a predicted feature of formative epiblast (Mak et al., 2004; Shiura and Abe, 2019).

Mouse ESCs undergo formative transition when withdrawn from 2iLIF (Hayashi et al., 2011; Kalkan et al., 2017; Mulas et al., 2017). We applied $A_{lo}XR$ during this transition and obtained continuously proliferating epithelial cells. Cultures displayed variable levels of heterogeneity during the first few passages (Figure S1I) but stabilized within 4–6 passages and subsequently expanded similarly to embryo-derived FS cells. We replated cultures in 2iLIF, which supports clonal propagation of ESCs at high efficiency (Kalkan et al., 2017). All cells died or differentiated within a few days, demonstrating complete extinction of ESC identity. This finding is in marked contrast to other reports of "intermediate" pluripotent states, which readily revert to ESCs (D'Aniello et al., 2016; Neagu et al., 2020; Rathjen et al., 1999).

### Germline and Somatic Lineage Induction *In Vitro*

In mice, the formative phase of pluripotency is definitively distinguished from naive and primed phases by competence for germline specification (Hayashi et al., 2011; Ohinata et al., 2009). We examined the response of embryo-derived $A_{lo}XR$ cells to the cytokine cocktail for PGC induction (Ohinata et al., 2009). In each of 8 independent lines tested, we detected the PGC surface marker phenotype $CD61^{+}SSEA1^{+}$ (Figure 1F). This capacity was maintained even in late passage (>P30) cultures. The proportion of marker-positive cells ranged up to >30% in some experiments and was generally between 5%–25%, although one line was consistently less efficient, around 1%. Two lines expanded without RARi also produced $CD61^{+}SSEA1^{+}$ immunopositive cells, albeit at <10% (Figure S1J). In contrast, 4 AFX EpiSC lines derived from E5.5 epiblast did not yield double-positive cells (Figure S1K). Furthermore, AFX EpiSCs adapted to culture in $A_{lo}XR$ over several passages remained unable to produce PGC-like cells (PGCLCs) (Figure S1L).

To confirm PGCLC identity, we sorted the $CD61^{+}SSEA1^{+}$ population and verified expression of a range of germ cell markers by qRT-PCR (Figure S1M). We also observed co-expression of

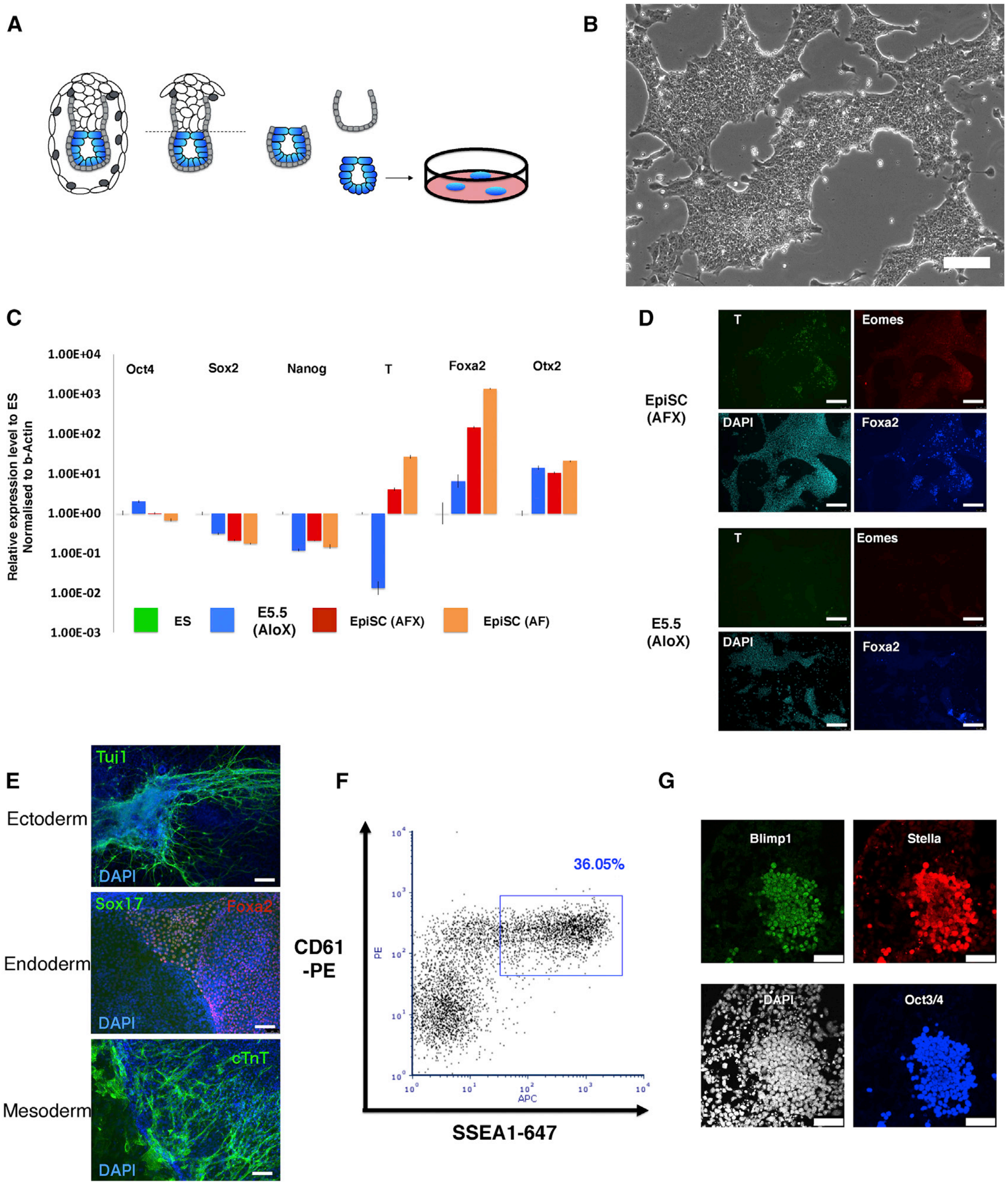


**Figure 1. Derivation of Stem Cell Lines from Formative Epiblast**

(A) Schematic of cell line derivation from E5.5 epiblast.

(B) Image of serially passaged E5.5-epiblast-derived culture. Scale bar, 100 μm.

(C) qRT-PCR analysis of marker gene expression relative to ESCs in 2iL (=1) in $A_{lo}X$ cells and EpiSCs maintained in either activin and FGF (AF) or activin, FGF, and XAV939 (AFX), normalized to beta-actin. Error bars are SD from technical triplicates.

(D) Immunofluorescent staining of EpiSCs and $A_{lo}X$ cultures for early lineage markers. Scale bars, 150 μm.

(E) Immunostaining of embryoid body outgrowths for germ layer markers; DAPI in blue. Scale bars, 150 μm.

(F) Flow cytometry analysis of PGCLC induction at day 4.

(G) Immunostaining of day 4 PGCLC. Scale bars, 50 μm.

Oct4, Blimp1, and Stella proteins by immunostaining in both $A_{lo}XR$ and $A_{lo}X$ cultures (Figures 1G and S1N). Collectively, these features constitute recognized hallmarks of mouse PGCLCs (Hayashi et al., 2011; Ohinata et al., 2005). Based on this competence, we designated $A_{lo}X$ and $A_{lo}XR$ cells as formative stem (FS) cells.

We then investigated directed somatic differentiation of FS cells in comparison with EpiSCs. Inhibition of the Wnt pathway

shifts the character of EpiSCs toward anterior epiblast identity and predisposes them to neuroectodermal fate (Osteil et al., 2019; Tsakiridis et al., 2014). We used the Sox1::GFP reporter (Stavridis and Smith, 2003) to quantify neural induction kinetics of FS cells and EpiSCs maintained with Wnt inhibition. After transfer into permissive N2B27 medium, more than 80% of EpiSCs became GFP positive on day 1 compared with only around 25% of FS cells (Figure 2A). By day 2, however, the GFP+ fraction approached 80% for FS cells and by day 3 reached >80% as for EpiSCs. We examined protein expression by immunostaining and found that FS cells lagged behind EpiSCs in both downregulation of Oct4 and upregulation of Sox1, but by day 3, the vast majority were Oct4 negative and Sox1 positive (Figure 2B). Thus, mouse FS cells have similar capacity to form neuroectoderm as EpiSCs but take longer to do so.

We tested primitive-streak-like induction in response to activin and GSK3 inhibition (Burgold et al., 2019). We observed substantially higher induction of mesendoderm surface markers and gene expression from FS cells than from EpiSCs (Figures S2A–S2C). Using flow cytometry, we quantified Flk1$^{+}$Ecad$^{-}$ lateral mesoderm and Cxcr4$^{+}$Ecad$^{+}$ definitive endoderm. We detected no induction of either lineage directly from ground state ESCs and only modest induction from EpiSCs (Figures 2C and 2E). Across a panel of FS and EpiSC lines, induction of mesoderm was on average 3-fold more efficient from FS cells (Figure 2D) and induction of endoderm was 4-fold higher (Figure 2F).

To probe the basis of differential propensity for primitive streak induction, we examined the response of ESCs, FS cells, and EpiSCs to signals operative during gastrulation. Ground-state ESCs did not upregulate *T* in response to any stimulus tested, with the exception of very low induction by the GSK3 inhibitor CH. EpiSCs also failed to show any appreciable response, apart from induction by CH at 6 h that was not maintained at 24 h. In contrast, FS cells showed sustained upregulation of *T* upon treatment with activin, FGF, CH, or, to a lesser extent, BMP (Figure 2G). Notably, addition of FGF at only 1 ng/ml induced *T* and *FoxA2* expression in FS cells (Figure S2D)

Thus, FS cells show rapid and efficient responsiveness to primitive streak inductive cues but require 48 h for full neural specification. These behaviors are distinct from EpiSCs and consistent with a developmental stage of E5.5–6.0 epiblast.

## Chimera Colonization

EpiSCs (AF) do not normally contribute to blastocyst injection chimeras unless they have been genetically modified to enhance ICM integration or survival (Masaki et al., 2016; Ohtsuka et al., 2012; Tesar et al., 2007). We confirmed this finding for AFX EpiSCs derived from E5.5 epiblast, detecting no mid-gestation chimeras after blastocyst injection of three lines and transfer of 95 embryos. We tested whether FS cells may have a higher probability of enduring from the E3.5 blastocyst until stage-matched early post-implantation epiblast. Following blastocyst injection of three different embryo-derived FS cell lines engineered to express mKO2 or GFP, we saw reporter expression in multiple E9.5 embryos (Figures 3A and S3A–S3E). Contributions are low to moderate compared with typical ESC chimeras and tend to be patchy rather than evenly dispersed. Nonetheless, colonization may be spread over multiple tissue types, including Sox2-positive putative migratory PGCs (Figure 3B). We examined genital ridge contribution at E12.5 and detected mKO2-reporter-positive Oct4$^{+}$ Mvh$^{+}$ PGCs (Figures 3C, S3F, and S3G). By fluorescence imaging, we observed contributions to three newborn pups. Two of these animals developed to adulthood and one was euthanized at post-natal day 21 (P21) due to malocclusion. Post-mortem tissue inspection revealed contributions to brain, bone, skin, heart, lung, and gut (Figure 3D). In addition, we obtained several overt coat color chimeras (Figure 3E).

Chimera formation conceivably might entail reversion of FS cells to naive status in the blastocyst. We therefore inspected embryos 24 h after injection. FS cells were localized to the ICM, but immunostaining showed that in contrast to host naive epiblast or introduced ESCs, FS cells did not express the naive pluripotency specific transcription factor Klf4 and retained the formative marker Oct6 (Figure 3F). Therefore, FS cells maintain formative identity within the blastocyst environment.

Chimera formation by FS cells derived from post-implantation epiblast challenges the conclusion from classic embryo-embryo chimera studies that epiblast cells lose colonization ability entirely by E5.5 (Gardner and Brook, 1997; Gardner et al., 1985). We revisited those experiments by using a fluorescent reporter to allow sensitive detection of contributions. We dissected epiblasts from cavitated E5.5 and pre-streak E6.0–6.25 transgenic embryos expressing membrane-bound tdTomato (mTmG). Epiblasts were dissociated using Accutase with addition of ROCKi to improve viability and 10 cells injected per blastocyst. We detected tdTomato-positive cells in 11 out of 91 embryos recovered at E9.5 (Figures 3G, 3H, and S3H–S3L). Contributions were typically sparse and, interestingly, were most frequently in the yolk sac mesoderm and amnion. In three chimeras, however, colonization was widespread in the embryo proper (Figures 3G, 3H, and S3H). We did not detect any contribution from streak stage (E6.5–7.0) epiblast cells (Figure S3L).

These observations establish that FS cells and primary formative epiblast cells can contribute to blastocyst chimeras, although with lower efficiency than ESCs or ICM cells.

## Transcriptome Relatedness to Pre-streak Epiblast

For global evaluation of cellular identity, we performed RNA sequencing (RNA-seq). We first compared FS cells with ground-state ESCs and with EpiSCs cultured in AF or AFX. Principal component analysis (PCA) grouped ESCs apart on PC1, whereas the two types of EpiSCs and FS cells were resolved on PC2 (Figure 4A). Differential expression analysis ($Log_2$ fold change, >1.4; adjusted $p < 0.05$) identified 531 and 266 genes upregulated and 941 and 168 genes downregulated in FS cells relative to AF and AFX EpiSCs, respectively (Figures S4A ad S4B). Gene Ontology (GO) term enrichment analysis highlighted “cell adhesion” in FS cells, contrasting with gastrulation and development in EpiSCs (Figures S4A and S4B). We identified 328 genes that are upregulated in FS cells compared with ESCs or either class of EpiSC (Figure 4B), with GO term enrichment for “ion transport” and cell adhesion (Figure 4C).

We then used a low cell number RNA-seq protocol with deep read depth (Boroviak et al., 2015) for comparison of FS cells with dissected pre-cavitation (E5.0), early cavitation (E5.5), and pre-streak (E6.0) epiblast. Unsupervised hierarchical clustering showed FS cell relatedness to E5.5 and E6.0 epiblast, with a

CellPress
OPEN ACCESS

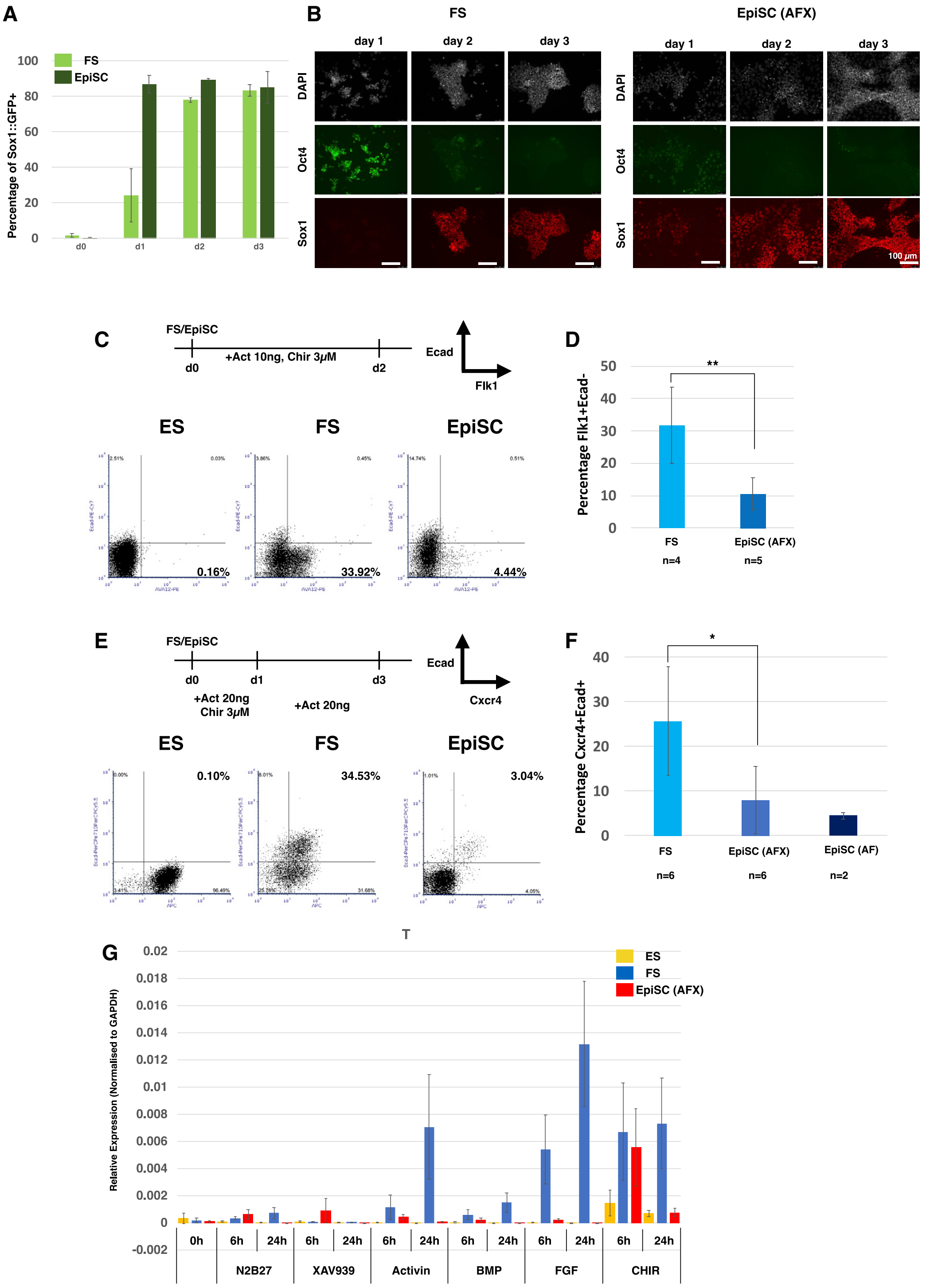


**Figure 2. Lineage Potency of FS Cells and Responsiveness to Differentiation Cues**

(A) Neural differentiation assayed by quantification of Sox1::GFP-positive cells. Error bars represent SD from 4 independent experiments.

(B) Immunostaining of FS cells and EpiSCs during neural differentiation; DAPI in white. Scale bars, 100 μm.

(C) Lateral plate mesoderm differentiation and representative quantifications of the $Flk1^{+}Ecad^{-}$ fractions by flow cytometry.

(D) Average efficiency of Flk1-positive cell production from FS cells and EpiSCs. n, independent cell lines assayed. Error bars represent the SD. $^{**}p < 0.01$.

(E) Definitive endoderm differentiation protocol and representative quantifications of the $Cxcr4^{+}Ecad^{+}$ fraction.

(F) Average proportion of $Cxcr4^{+}Ecad^{+}$ double-positive cells from differentiation of FS and EpiSC lines. Error bars represent SD; $^{*}p < 0.05$.

(G) *T* expression analyzed by qRT-PCR 6 h and 24 h after transfer into N2B27 medium with the indicated supplements; 2 μM XAV939, 20 ng/ml activin A, 10 ng/ml BMP2, 12.5 ng/ml Fgf2, and 3μM CH. Relative expression is normalized to GAPDH. Error bars are SD from two independent cell lines and two technical replicates.

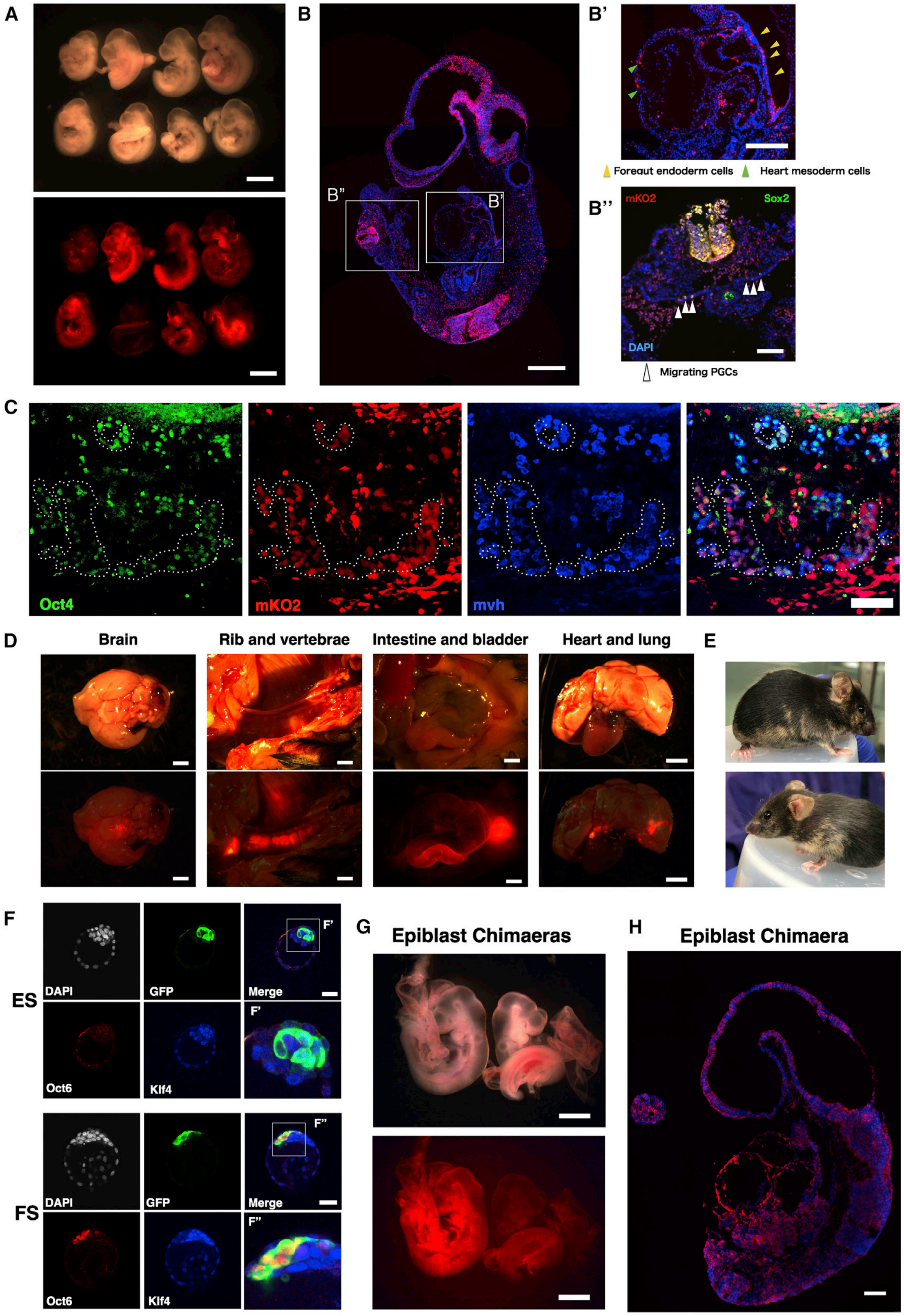


(legend on next page)

lower correlation to the pre-cavitation stage (Figure 4D). EpiSCs, both AF and AFX, were less related to the pre-gastrula epiblast stages. We identified 953 differentially expressed genes between FS cells and EpiSCs. This gene set clustered published embryo and EpiLC single-cell data (Nakamura et al., 2016) by developmental trajectory (Figure 4E). Our RNA-seq E5.5 and E6.0 epiblast profiles projected onto this PCA aligned with E5.5 and EpiLC single cells (Figure 4E). FS cells overlapped with EpiLCs, between E5.5 and E6.5 $T_{Lo}$, whereas EpiSCs were positioned with the E6.5 cells. We inspected several of the FS-cell-specific genes (Figure 4B) and detected dynamic expression in the embryo single-cell data with enrichment at E5.5 (Figures 4F and S4C).

We performed single-cell analysis on FS cells and EpiSCs by using the Smart-seq2 method (Picelli et al., 2014). Applying a threshold of 3 million reads, we examined 326 cells. FS cells from two independent lines formed a single cluster in the PCA plot (Figure 4G), separated from EpiSCs on PC1. Notably, there was no overlap between EpiSCs and FS cells. PC2 separated AF and AFX EpiSCs. Measurement of gene expression correlation by the Jaccard index showed that FS cells are more homogeneous than either class of EpiSC (Figure 4H).

Collectively, these analyses indicate that FS cells capture features of pre-streak epiblast and EpiLCs but are less related to later stage epiblast and EpiSCs.

## Growth Factor Requirements for FS Cell Propagation

As potential autocrine stimuli of self-renewal or differentiation, we evaluated Nodal, FGF, and Wnt family representation in the FS cell transcriptome data (Figures S4D–S4F). We found robust expression of *Fgf5* as expected but also detected several other FGFs at lower levels. However, *Fgf8*, which is active during primitive streak formation (Sun et al., 1999), was lowly expressed compared with EpiSCs. FS cells express both *Fgfr1* and *Fgfr2* (Figure S4D). We tested whether FS cell cultures are dependent on FGF signaling by adding specific inhibitors of FGF receptors (PD173074; 0.1 μM) or downstream MEK1/2 (PD0325901; 1 μM). Both inhibitors caused rapid collapse of FS cell cultures. We conclude that endogenous low-level expression of FGFs supports self-renewal, without inducing the primitive-streak-associated gene expression associated with exposure to exogenous FGF (Figures 2G and S2D).

FS cells express nodal/activin receptors but interestingly present lower mRNA levels for the co-receptor *Tdgf1* and for *Nodal* itself than either ESCs or EpiSCs (Figure S4E). We investigated further the requirement for nodal pathway stimulation. Addition of receptor inhibitors (A83-01 or SB505124) resulted in extensive cell death and differentiation with loss of Oct4 and upregulation of Pax6 (Figures 4I and S4G). Withdrawal of activin also led to reduced viability and increased differentiation, indicating that autocrine activity does not provide sufficient pathway stimulation. In FS cell medium, activin is added at only 3 ng/ml compared with 20 ng/ml typically used for feeder-free culture of EpiSCs. Dosage sensitivity is a well-known feature of nodal signaling in the mouse embryo (Robertson, 2014). We observed markedly less induction of nodal pathway targets in FS cells at 3 ng/ml than at 20 ng/ml activin (Figure 4J). Furthermore, immunoblotting indicated lower steady-state levels of phospho-Smad2 in cells passaged in 3 ng/ml activin (Figure 4K). These observations are consistent with a dose-dependent response to nodal/activin stimulation, whereby low signal sustains the formative gene regulatory network and higher signal promotes primitive streak specification.

Finally, the observed expression of Fzd receptors and low levels of some Wnts may underlie the requirement for inhibition of Wnt signaling to fully suppress differentiation (Figure S4F). Consistent with this interpretation, we observed that the porcupine inhibitor IWP2 could substitute for XAV939 during FS cell maintenance.

Thus, FS cells are maintained by FGF and nodal/activin but are poised to respond to increased levels of either signal or of canonical Wnt by entering into mesendoderm differentiation.

## Chromatin Accessibility in FS Cells

We used the assay for transposase accessible chromatin coupled to deep sequencing (ATAC-seq) (Buenrostro et al., 2013) to survey open chromatin in FS cells. Independent FS cell samples were well correlated (Figure 5A). We classified sites that exhibit differential accessibility between ESCs, FS cells, and EpiSCs based on a fold-change enrichment greater than two ($p < 0.05$). Reorganization was evident between naive and formative cells, with 3,742 sites closing, 4,259 opening, and only 207 shared open sites (Figures 5B and 5C). In contrast, between formative and primed cells, a majority of open sites were shared (3,588), whereas just over 1,000 became more accessible and a similar number closed. We detected 826 peaks specifically enriched in FS cells compared to either ESCs or EpiSCs. These FS-cell-specific open chromatin regions were also accessible in transient EpiLCs (Figures 5C and 5D). Nearby genes (<1 kb) showed no significant GO term enrichment, however (Figure S5A).

Chromatin immunoprecipitation sequencing (ChIP-seq) for histone modifications showed the expected correlation between open chromatin and active marks, H3K4me3, H3K4me1, and

**Figure 3. Blastocyst Chimera Contribution by FS Cells and Formative Epiblast**

(A) Bright-field and fluorescent images of E9.5 embryos generated after blastocyst injection of mKO2 reporter FS cells. Scale bar, 1 mm.

(B) Sagittal section from one chimera, stained for mKO2 and DAPI. (B'), mKO2-positive cells in foregut endoderm (yellow arrowheads) and cardiac mesoderm (green arrowheads). (B'') (rotated 90°), Sox2 immunostaining (white arrowheads) in the hindgut region. Scale bars, 200 μm (B) and 100 μm (B'and B'').

(C) mKO2-positive cells expressing Oct4 and Mvh PGC markers in E12.5 chimeric gonad. Triple-positive cells are highlighted with dashed circles. Scale bars, 75 μm.

(D) Fluorescent images of organs from post-natal day 21 (P21) chimera overlaid with 20% opacity bright-field image. Scale bars, 2 mm.

(E) Coat color chimeras generated from NBRA3.2 FS cells at 7 weeks (above) and 4 weeks (below).

(F) Blastocysts injected with GFP reporter ESCs or FS cells and cultured for 24 h. ESCs are $Klf4^{+}Oct6^{-}$ (n = 11) (F'), whereas FS cells are $Klf4^{-}Oct6^{+}$ (F'') (n = 15). Scale bars, 40 μm.

(G) E9.5 chimeras obtained from blastocyst injection of mTmG expressing E5.5 epiblast cells. Scale bars, 500 μm.

(H) Section from left embryo in (G) stained with anti-RFP to visualize membrane-tdTomato; DAPI in blue. Scale bar, 200 μm.

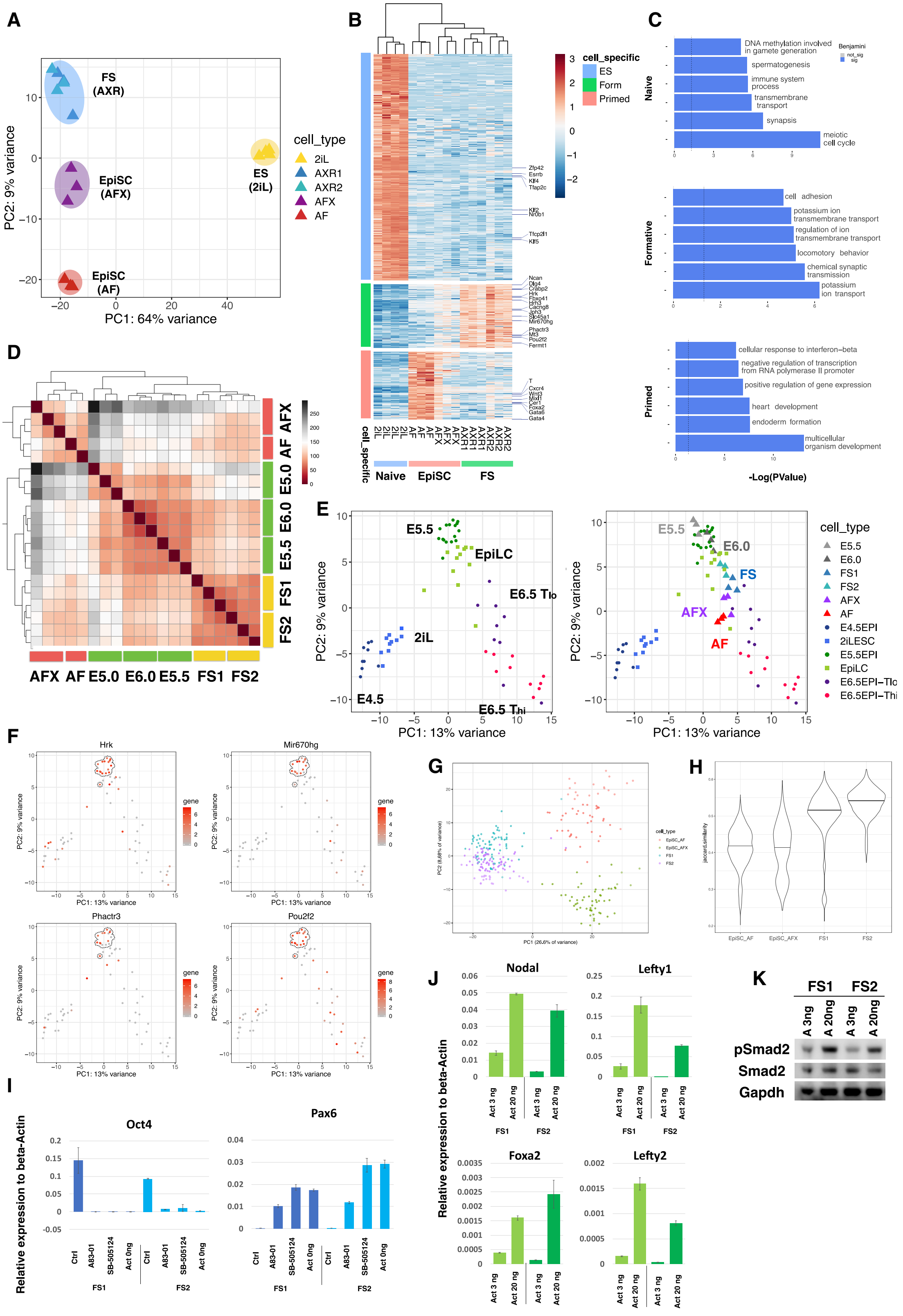


**Figure 4. Whole-Transcriptome Analysis and Nodal/Activin Pathway Activity**

(A) PCA with all genes for ESCs, FS cells, and EpiSCs (AFX and AF).

(B) Heatmap clustering of naive, formative, and primed enriched genes.

(legend continued on next page)

H3K27Ac (Figure 5E). Regions that were more open in naive and formative cells showed marked enrichment for H3K4me3 and H3K27ac that was lost in EpiSCs. Interestingly, active marks were also more highly represented in FS cells than in ESCs at loci that opened only in EpiSCs. We surveyed bivalent promoter regions marked with both H3K4me3 and H3K27me3 (Azuara et al., 2006; Bernstein et al., 2006). We enumerated 2,417 bivalent promoters in FS cells, nearly three times the number in ESCs (Figure S5B). Many, but not all, of these loci were also bivalent in EpiSCs. Figure S5C shows examples of different profiles. Among the FS-cell-specific bivalent promoters was *Prdm14*, encoding one of the key germ cell determination factors (Nakaki et al., 2013). Promoters for other germ cell genes *Tfap2c* and *Prdm1* are also bivalent in FS cells, consistent with being poised for expression (Figure 5F). In EpiSCs, however, *Prdm14* loses both marks, indicating the gene is inactivated. This chromatin change may be a decisive feature in the loss of competence for PGCLC induction in EpiSCs (Hayashi et al., 2011)

We also assessed DNA methylation at open chromatin regions by using published data for EpiLCs and EpiSCs (Zylicz et al., 2015). In EpiLCs, all ATAC peaks were hypomethylated. In EpiSCs, in contrast, only primed peaks maintained low methylation (Figure S5D).

Among genes proximal to shared ATAC peaks in FS cells and EpiSCs, we observed marked differential expression (Figure 5G). GO term analysis of genes more highly expressed in EpiSCs identified enrichment for heart development, multicellular organism development, and gastrulation (Figure S5E). These included gastrulation-associated genes such as *Cer1*, *Gsc*, and *Pax3*. FS-cell-enriched transcripts were more numerous but comprised genes without annotated functions in early development (Table S1).

We used HOMER (Heinz et al., 2010) to identify transcription factor binding motifs enriched in open chromatin regions (Table S2). Core pluripotency factor binding motifs for Oct4 and Oct4-Sox-T*cf.*-Nanog were over-represented in all three cell types. ESC ATAC peaks were also enriched for Tfcp2l1 and Prdm14 motifs, whereas those in EpiSCs featured Gsc, Brachyury, Slug, and Eomes motifs (Figures 5H and S5F). Both FS cells and EpiSCs showed increased accessibility of AP1/Jun sites. Finally, we noted that FS cell open chromatin showed specific enrichment for ETS-domain factor binding motifs.

## FS Cells and EpiSCs Show Contrasting Dependencies on Etv and Otx2

Previously, we presented evidence linking Etv5, an ETS factor of the PEA3 sub-family, to enhancer activation during pluripotency progression (Kalkan et al., 2019). We also showed that ESCs lacking Etv5 show diminished ability to make EpiSCs. Here, we used CRISPR-Cas9 to generate ESCs deficient for both *Etv5* and the related *Etv4*. *Etv4/5*-double-knockout (dKO) cells failed completely to produce EpiSCs upon transfer to AFX and differentiated into fibroblast-like cells (Figure S6A). This phenotype is more severe than that for the *Etv5* mutation alone. Somewhat unexpectedly, however, *Etv4/5*-dKO cells converted to epithelial culture in $A_{lo}$XR and subsequently expanded, albeit with persisting differentiation (Figures 6A and S6A). Relative to ESCs, naive factors were downregulated and post-implantation markers upregulated, including several targets of Etv5, such as *Fgf5*, *Otx2*, and *Pou3f1* (Figure 6B). We detected no compensatory upregulation of the third PEA3 member *Etv1*. *Etv4/5*-dKO FS cells differentiated readily by embryoid bodies and in directed protocols (Figures S6B–S6E), including induction of Blimp1$^+$, Stella$^+$, and Oct4$^+$ PGCLC (Figure S6F). However, when transferred to AFX, *Etv4/5*-dKO cells failed to convert to EpiSCs, lost expression of Oct4 within 3 days, and differentiated into fibroblasts with aberrant expression of *Pou3f1* (Figures 6C, 6D, and S6G). Introduction of an *Etv5* transgene to *Etv4/5*-dKO cells restored the ability to convert to EpiSCs (Figures 6E–6H). These results establish that Etv4 and Etv5 are not essential for lineage competence of FS cells and yet are required for the production of EpiSCs *in vitro*.

Otx2 is prominently upregulated early during formative transition *in vivo* and *in vitro* (Acampora et al., 2016; Kalkan et al., 2017) and is implicated in redirecting genome occupancy of Oct4 (Buecker et al., 2014; Yang et al., 2014). Intriguingly, *Otx2* is dispensable in both ESCs and EpiSCs (Acampora et al., 2013), but homozygous embryo mutants exhibit severe gastrulation phenotypes (Ang et al., 1996). We generated *Otx2* KO ESCs and investigated conversion into FS cells in $A_{lo}$XR. Epithelial colonies emerged and could be expanded for 4–5 passages but continuously differentiated into neural cells (Figure 6I). By passage 5, Oct4 and Nanog were downregulated, and the majority of cells were positive for Sox1 (Figure 6J). Cultures could not be maintained reliably thereafter. In contrast, *Otx2* mutant ESCs could be converted into stable Oct4-positive EpiSCs by direct transfer into AFX (Figure 6I); although, colonies frequently displayed aberrant expression of Sox1 as previously reported (Acampora et al., 2013; Figure 6J). BMP has been shown to enhance stability of *Otx2*-deficient EpiSCs (Acampora et al., 2013). We added BMP to two *Otx2*$^{-/-}$ FS cell cultures in $A_{lo}$XR but observed no suppression of differentiation (Figure S6H).

We also mutated *Otx2* directly in FS cells and observed that colonies became compact and dome-shaped, superficially resembling naive ESCs (Figures 6K, 6L, and 6M). When replated in 2iL, however, *Otx2* mutant FS cells did not expand but instead

(C) GO term analyses based on the genes identified in (B). x axis is −Log(p value). Top 6 significant terms are shown (Benjamini value, <0.05).
(D) Heatmap comparison of FS cells and AFX and AF EpiSCs with E5.0, E5.5, and E6.0 epiblast cells.
(E) Left, PCA with mouse single-cell data from embryos and EpiLCs (Nakamura et al., 2016). Right, samples from (D) were projected onto the single-cell PCA.
(F) Gene expression patterns of selected FS cell enriched genes identified in (B) colored on PCA from (E). E5.5 epiblast cells are highlighted by the dashed circle.
(G) PCA using 2,000 most abundant genes of single-cell RNA sequencing (scRNA-seq) data from two FS cell lines and one AFX and one AF EpiSC line.
(H) Violin plot of Jaccard index analysis of 2,000 most abundant genes shows higher correlation between FS cells than EpiSCs.
(I) qRT-PCR analysis of FS cells in AloXR (Ctrl), with addition of 1 μM A83-01 or 5 μM SB5124, or withdrawal of activin for 2 days. Relative expression to beta-actin. Error bars are SD from technical duplicates.
(J) qRT-PCR analysis of FS cells cultured in low (3 ng/ml) and high (20 ng/ml) activin for 2 days. Relative expression to beta-actin. Error bars are SD from technical duplicates.
(K) Immunoblot analysis of phospho-Smad2. Cells were passaged once with low (3 ng/ml) or high (20 ng/ml) activin A before assay.

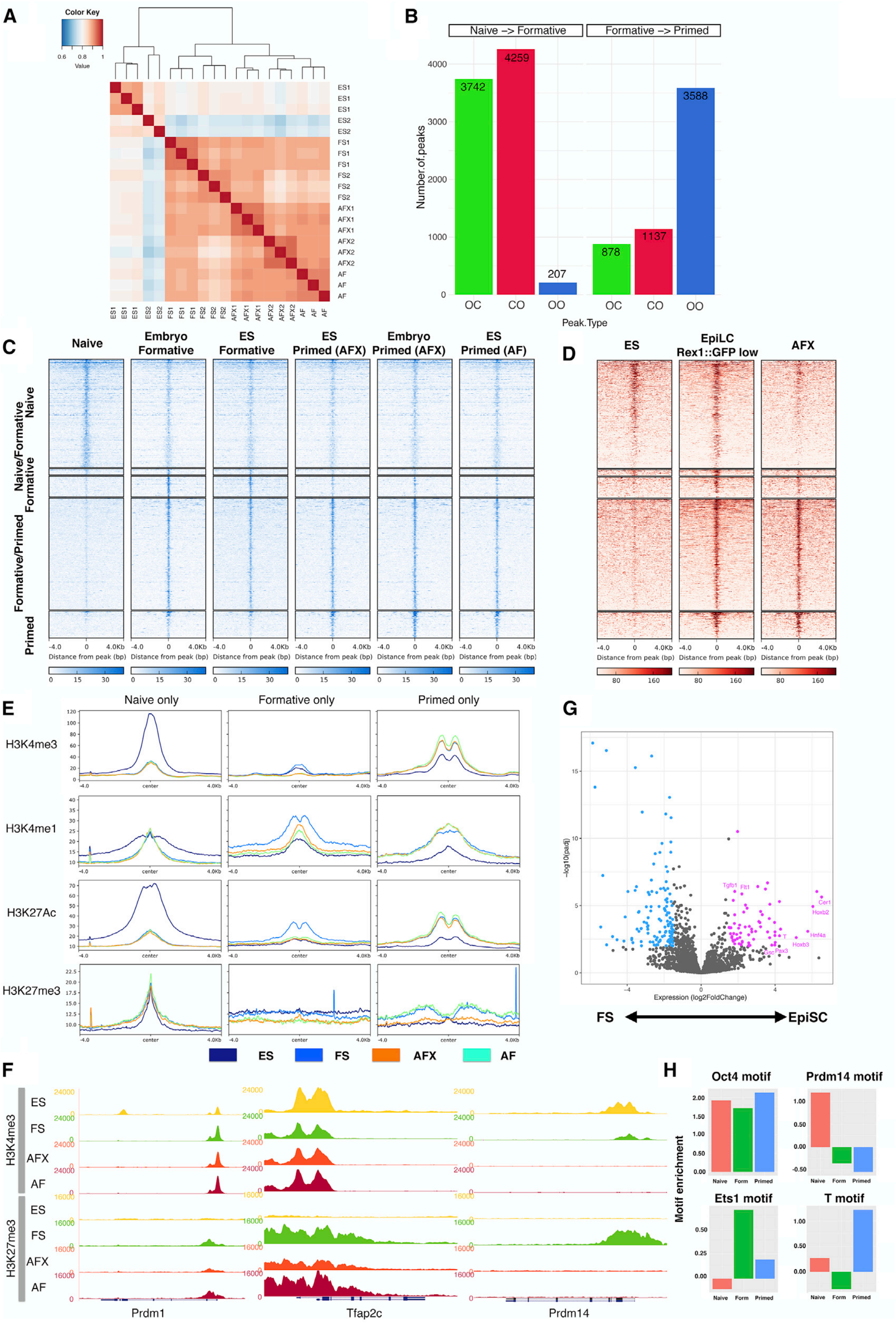


(legend on next page)

differentiated or died (Figure S6I). We managed to achieve initial clonal expansion of targeted FS cells in $A_{lo}$XR, but 8 out of 8 clones subsequently underwent extensive neural differentiation and could not be stably propagated. We added BMP to three cultures, but this did not result in stabilization.

These results indicate that Otx2 but not Etv4/5 is required for a stable FS cell state and conversely for EpiSCs.

## Generation of Human FS-like Cells

We explored the derivation of FS cells from naive human pluripotent stem cells (hPSCs) (Takashima et al., 2014). We used both chemically reset lines, namely, cR-H9EOS and cR-Shef6 (Guo et al., 2017), and embryo-derived HNES1 cells (Guo et al., 2016). $A_{lo}$X and $A_{lo}$XR were applied as for mouse FS cell culture, except that plates were coated with a combination of laminin and fibronectin to improve attachment. The domed naive hPSCs converted to a more flattened epithelioid morphology over several days. Cultures could be propagated continuously thereafter and exhibited a faster doubling rate than naive cells, requiring passage every 4 days at a split ratio of 1/15 (Figure 7A). Cells in $A_{lo}$XR lost naive markers (KLF4, KLF17, and TFCP2L1) but retained the core pluripotency factor OCT4, with little or no upregulation of lineage priming markers, TBXT or FOXA2, often detected in conventional hPSCs (Figure 7B; Allison et al., 2018; Gokhale et al., 2015). They showed gain of SOX11 and OTX2, markers of post-implantation epiblast in the primate embryo (Nakamura et al., 2016).

Naive hPSCs do not respond productively to somatic lineage induction protocols but must first undergo formative transition to lineage competence (Guo et al., 2017). This capacitation process takes place over several days (Rostovskaya et al., 2019). FS cells, in contrast, are expected to be directly responsive to lineage cues. We applied established protocols for differentiation to human FS cells. In response to definitive endoderm induction (Loh et al., 2014), we observed efficient formation of SOX17-positive cells (Figure 7C), whereas neural induction by dual SMAD inhibition (Chambers et al., 2009) resulted in abundant SOX1 immunopositive cells (Figure 7D). We also tested paraxial mesoderm differentiation (Chal et al., 2016) and detected upregulation of *TBX6* and *MSGN1* along with EMT markers such as *SNAIL1* and *ZEB1* (Figure 7E).

We prepared RNA-seq libraries from three human FS-like cell lines and carried out a whole-transcriptome comparison with naive and conventional hPSCs (Figure 7F). PCA distinguished naive cells on PC1 and separated formative from conventional hPSCs on PC2, similar to the analysis of mouse PSCs (Figure 4A). As a reference for *in vivo* early post-implantation development, we used data for the non-human primate *Macaca fascicularis* (Nakamura et al., 2016). We used 9,324 expressed orthologous genes (median Log2 expression, >0.5) to compute the PCA for *Macaca*, onto which we projected the human cell line samples (Figure 7G). FS-like cells and conventional hPSCs aligned with post-implantation embryo stages. FS-like cell samples were positioned adjacent to post-implantation epiblast, whereas conventional hPSCs spread further toward early gastrulating cells.

Single-cell transcriptome data have recently been published for human embryos during extended culture (Xiang et al., 2019). We used variable genes in the epiblast and primitive streak anlage (PSA) stages to compute the PCA for naive, formative, and conventional hPSCs and then projected the embryo single cells. The resulting plot shows a similar pattern to the *Macaca* embryo comparison. Naive cells clustered with pre-implantation epiblast, and formative cells were next to post-implantation stages. Conventional hPSCs were adjacent to FS cells but distributed more toward the PSA cluster (Figure 7H).

We performed K-means clustering (k = 6) between FS-like and conventional PSC cultures (Figure S7A). Cluster 1 comprises 369 genes expressed more highly in FS cells than conventional hPSCs. The majority of protein-coding genes in this cluster are expressed in naive cells and persist during capacitation (Figures S7B and S7C). *DPPA2*, *GDF3*, and several *ZNF* genes were identified as useful markers expressed in both naive and formative cells but variably low or absent in conventional hPSCs (Figures 7I and S7D). Expression of these ZNF genes was detected in human pre- and post-implantation epiblast transcriptome data (Figure 7J).

KRAB-ZNFs such as ZNF676, ZNF560, and ZNF528 can suppress the expression of transposable elements (TEs) (Friedli and Trono, 2015). TEs are dynamically expressed in early development and are highly differential between naive and primed hPSCs (Friedli and Trono, 2015; Guo et al., 2017; Theunissen et al., 2016). We examined TE expression in FS-like cells and observed a distinct profile compared with naive or conventional hPSCs (Figure 7K). For example, FS-like cells distinctively expressed LTR6A and retained expression of certain HERVK TEs also expressed in naive cells but did not express subsets of SVA family members that are prominent in naive cells, nor subsets of HERVH, LTR7C, or LTR12C family members that are prominent in primed cells (Figure S7E).

Finally, we investigated application of FS cell culture conditions directly to human ICM explants that are known to transition to early post-implantation stages (O'Leary et al., 2012). We thawed E5 and E6 blastocysts and cultured for 1 or 2 days, respectively, in N2B27. We then isolated ICMs by immunosurgery or manual dissection and plated them intact on laminin/fibronectin-coated dishes in $A_{lo}$XR with ROCK inhibitor. After 2–4 weeks, primary outgrowths were manually dissociated and re-plated. We established

**Figure 5. Chromatin Landscape Analysis**

(A) Hierarchical clustering of all ATAC-seq peaks.

(B) Peak changes between states. OC, open to closed; CO, closed to open; OO, open to open.

(C) Heatmaps of differential ATAC-seq peaks.

(D) Heatmaps of ATAC-seq peaks from (C) in EpiLCs and EpiSCs derived from RGd2 ESCs.

(E) Histone modification patterns at ATAC-seq peaks.

(F) Genome browser screenshots of H3K4me3 and H3K27me3 distribution at *Prdm1*, *Tfap2c*, and *Prdm14* loci.

(G) Volcano plot showing expression fold changes for genes associated with ATAC-seq peaks shared between FS cells and EpiSCs. Purple, upregulated in EpiSCs; blue, upregulated in FS cells.

(H) Transcription factor binding motif enrichments at ATAC-seq peaks.

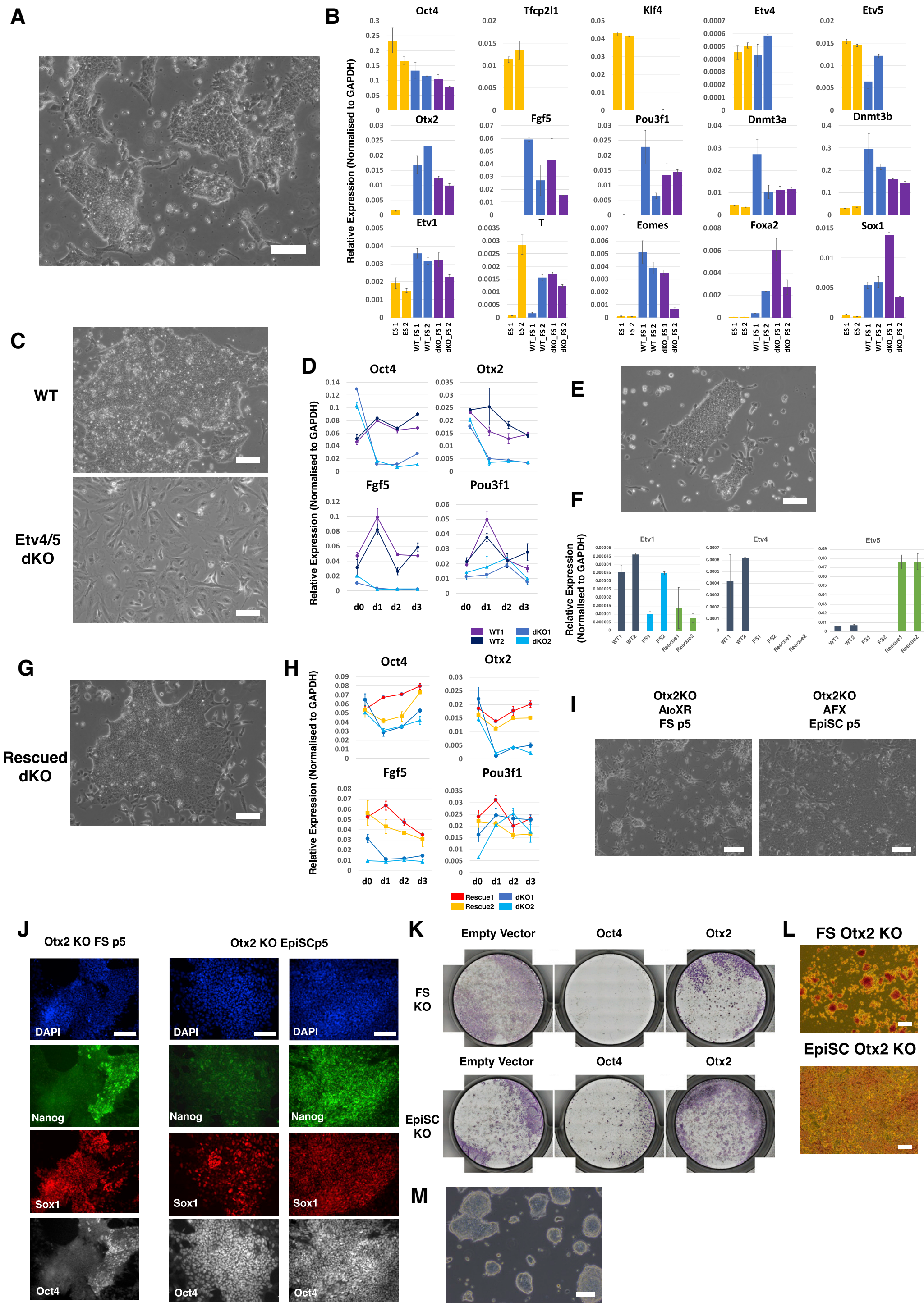


**Figure 6. Differential Requirements for *Etv4/5* and *Otx2***

(A) Morphology of *Etv4/5* dKO FS cells.

(B) qRT-PCR analysis of ESCs (yellow), parental (wild-type [WT]) FS cells (blue), and *Etv4/5*dKO FS cells (purple). Error bars represent SD from technical duplicates.

(C) Morphology of WT and dKO FS cells in EpiSC (AFX) culture medium for 3 days.

(D) Time course qRT-PCR analysis of WT and *Etv4/5*dKO FS cells in EpiSC (AFX) culture. Error bars are SD from technical duplicates.

(E) Morphology of *Etv4/5*dKO FS cells expressing *Etv5* transgene.

(F) qRT-PCR assay of *Etv1*, *-4*, and *-5* in *Etv5* rescue dKO lines. Error bars represent SD from technical duplicates.

(G) Morphology of rescued dKO FS cells in EpiSC (AFX) culture.

(legend continued on next page)

three lines from different embryos. The embryo-derived lines exhibited similar morphology and growth behavior to naive PSC-derived FS-like cells (Figure 7L). G-banded karyotype analysis showed that all three expanded lines were diploid (46XX; 20/20) (Figure S7F). We confirmed a relatively homogeneous expression of OCT4, SOX2, and NANOG by immunostaining (Figure 7M). Expression of naive-specific transcription factors KLF4 and KLF17 was not detected, whereas transcripts were present for several genes that are expressed in naive and formative cells but downregulated in conventional hPSCs (Figure 7N).

## DISCUSSION

Expandable stem cells that retain high fidelity to staging posts of pluripotency in the embryo will be instrumental in harnessing a capacity to recapitulate development, create disease models, and manufacture therapeutic cells. Stem cells representative of naive and primed pluripotency have been established in mice and humans (Davidson et al., 2015; Nichols and Smith, 2009; Rossant, 2015; Rossant and Tam, 2017), but formative pluripotency has only been obtained in the form of transient EpiLCs (Buecker et al., 2014; Hayashi et al., 2011; Kalkan et al., 2017; Mulas et al., 2017). The findings in this study fill the stem cell gap between early and late pluripotency.

Mouse ESC derivatives with features of late blastocyst or peri-implantation epiblast, such as reduced Rex1 or increased Otx2, have been reported previously (D'Aniello et al., 2016; Neagu et al., 2020; Rathjen et al., 1999). However, those cells spontaneously reverted to the canonical ESC phenotype when transferred to ESC culture. Therefore, they remain within the naive spectrum. Significantly, the cytokine LIF, which potently promotes mouse ESC identity (Dunn et al., 2014; Smith et al., 1988; Williams et al., 1988), is a key component of all these culture conditions. In contrast, FS cells are maintained without LIF and have extinguished ESC identity, which is in line with the inability of peri-implantation epiblast to form ESCs (Boroviak et al., 2014).

In mice, a defining functional attribute of formative epiblast is direct responsiveness to germline induction, which is lacking in both naive cells and primed gastrula stage epiblast (Ohinata et al., 2009). Conversion of ESCs into transient EpiLC populations generates a window of germline competence (Hayashi et al., 2011). However, maintenance of competence over many passages is a unique feature of mouse FS cells, signifying stabilization of a transient embryonic state.

Mouse FS cells also differ from ESCs and EpiSCs in their contribution to chimeras. Chimerism is less frequent, to lower levels, and less evenly distributed than typically obtained with ESCs. Poorer contributions are not unexpected given the heterochronicity between FS cells and E3.5 host blastocysts. Pioneering mouse embryo chimera studies suggested that blastocyst colonization capacity was lost entirely after implantation (Gardner, 1985). Here, using more sensitive detection systems and injecting 10 cells rather than single cells with ROCKi to improve viability, we found that formative epiblast cells can contribute to blastocyst chimeras, similarly to FS cells. EpiSCs, in contrast, do not generally show any significant contribution to chimeras by blastocyst injection, unless they have been genetically engineered (Masaki et al., 2016; Ohtsuka et al., 2012; Tesar et al., 2007). Intriguingly, it has been reported that certain EpiSC lines cultured on feeders or serum-coated dishes contain a sub-population of cells that are able to contribute to chimeras (Han et al., 2010; Kurek et al., 2015). The nature of such cells is unclear, but our results raise the possibility that they may represent FS cells co-existing with EpiSCs under those undefined conditions.

FS cells exhibit distinct signal dependency and responsiveness compared to ESCs or EpiSCs. Both mouse EpiSCs and human conventional PSCs are cultured in medium supplemented with FGF. Indeed, high FGF (100 ng/ml) is considered an essential component of defined E8 medium for hPSCs (Chen et al., 2011; Cornacchia et al., 2019). FS cells, in contrast, are cultured without FGF supplementation. Notably mouse FS cells respond directly to FGF or other stimuli for primitive streak induction by upregulating *T*. Consistent with a readiness for *T* induction, FS cells exhibit a greater propensity to form mesendoderm than EpiSCs. We surmise that the relative recalcitrance of EpiSCs to primitive streak induction may reflect adaptation to the high growth factor signals that drive their *in vitro* proliferation. FS cells are also efficient at entering the neural lineage but, consistent with an earlier stage of epiblast, do so more slowly than EpiSCs. High competence for germline, primitive streak, and neural induction are features of pre-streak formative epiblast. Whole-transcriptome analysis substantiates this identity and further confirms that mouse FS cells are related to EpiLCs and are distinct from EpiSCs.

FS cells and EpiSCs show different transcription factor dependencies. FS cells are mildly destabilized by deletion of *Etv5* and *Etv4* but remain expandable and pluripotent, whereas the EpiSC state cannot be established without these factors (Kalkan et al., 2019). Whether the inability to produce *Etv4/5* dKO EpiSCs results from a cryptic change in formative competence or reflects a specific function in EpiSCs remains to be clarified. Interestingly, a proportion of *Etv5* or *Etv4/5* mutants proceed through gastrulation (Lu et al., 2009; Zhang et al., 2009). The *Etv4/5* knockout phenotypes therefore suggest that the *in vitro* EpiSC state may not be fully representative of epiblast progression *in vivo* (Kojima et al., 2014). Conversely, Otx2, which is necessary for *in vivo* gastrulation (Ang et al., 1996), is not required by ESCs or EpiSCs (Acampora et al., 2013) but is indispensable for the stable expansion of FS cells. Defective formative transition may also underlie the precocious neural differentiation of EpiSCs lacking Otx2 (Acampora et al., 2013).

(H) Time course qRT-PCR analysis of rescued lines. Error bars represent SD from technical duplicates.

(I) Phase images of *Otx2* KO ESCs transferred to FS cell or EpiSC (AFX) culture conditions for 5 passages.

(J) Immunostaining of *Otx2* KO cells at passage 5 (p5) in FS cell or EpiSC culture. Two classes of EpiSC colony were observed: left, homogeneous Oct4 with heterogenous Nanog and Sox1; right, uniformly Oct4, Sox1, and Nanog triple positive.

(K) Alkaline phosphatase (AP) staining of control and *Oct4* and *Otx2* KOs generated by Cas9/guide RNA (gRNA) transfection in FS cells and EpiSCs. Colonies were stained 3 days after replating transfected cells.

(L) Morphology of AP-positive *Otx2* KO FS cells and EpiSCs.

(M) Representative image of *Otx2* KO FS cells before culture collapse. Scale bars, 100 μm, except (J) 50 μm.

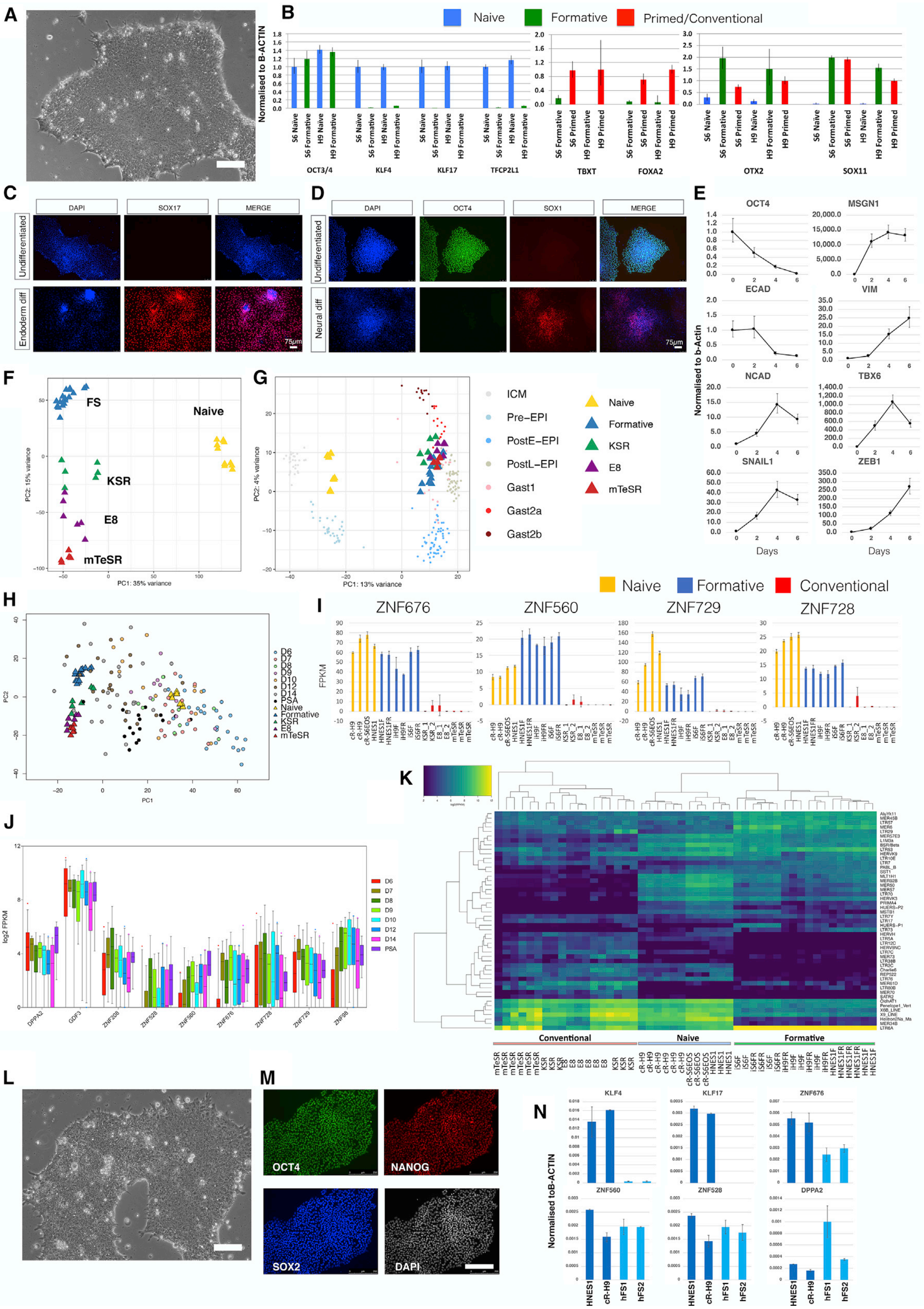


(legend on next page)

In FS cells, the transcription factor circuitry governing naive pluripotency (Dunn et al., 2014; Takashima et al., 2014) is dismantled, signaling pathways are rewired, and chromatin accessibility is extensively remodeled compared to ESCs. These events indicate a step change as cells transition from naive to formative pluripotency. By contrast, the separation between FS cells and primed pluripotent stem cells is blurred, which is in line with more continuous developmental progression. We surmise that the gene regulatory network and chromatin landscape are reconfigured in formative cells to provide the requisite context for signaling cues to induce germ layer and germline lineage specification and the subsequent unfolding of gastrulation. Capture of formative phase cells as self-renewing stem cell cultures should facilitate deep interrogation of the machinery that confers multi-lineage potency.

## Limitations of Study

Although the formative phenotype is reached within 48 h of ESC withdrawal from 2i, generation of stable FS cell lines requires several passages. The inherent asynchronicity of exit from naive pluripotency (Strawbridge et al., 2020) together with imperfect *in vitro* transition conditions result in initial heterogeneity, as also observed for EpiLC formation (Hayashi et al., 2011; Kalkan et al., 2017). Passaging enriches for FS cells, similar to stabilization of EpiSC cultures (Guo et al., 2009), but a more streamlined and efficient capture would be advantageous for future research. In mice, FS cells are unambiguously distinguished from EpiSCs by several features, most notably competence for germ cell induction and ability to colonize chimeras by blastocyst injection. Neither of those functional criteria are applicable in the human context. Conventional hPSCs share some features with EpiSCs but do not appear to be direct equivalents (Lau et al., 2020; Rossant and Tam, 2017). Notably, they can be induced to form PGCLCs (Irie et al., 2015; Sasaki et al., 2015). Chimera contribution cannot be tested in human embryos. At the transcriptome level, human FS-like cells differ from populations of conventional hPSCs cultured in E8 or other conditions, but these differences are relative rather than absolute. Heterogeneity and hierarchical substructure has been described in hPSC cultures (Allison et al., 2018; Hough et al., 2009, 2014; Lau et al., 2020; Nakanishi et al., 2019), and we cannot exclude the presence of FS cells at some frequency. Human FS cells and conventional hPSCs may be a continuum spanning post-implantation epiblast progression. It will be valuable in future studies to define marker sets and *in vitro* differentiation behaviors that can better distinguish human formative cells from downstream stages in the spectrum of post-naive pluripotency. To this end, additional transcriptomic and other data on post-implantation epiblast will be important to allow more precise comparison and staging.

## STAR★METHODS

Detailed methods are provided in the online version of this paper and include the following:


- KEY RESOURCES TABLE
- RESOURCE AVAILABILITY
  - Lead Contact
  - Materials Availability
  - Data and Code Availability
- EXPERIMENTAL MODEL AND SUBJECT DETAILS
  - Mice
  - Human Embryos
  - Cell Cultures
  - Mouse FS cell, EpiSC and ES cell culture
  - Derivation of FS and EpiSCs from mouse embryo
  - Derivation of FS and EpiSCs from mouse ES cells
  - Derivation of human FS cells from naive PSCs
  - Derivation of human FS cell from embryos
- METHOD DETAILS
  - Embryoid body differentiation
  - PGCLC differentiation
  - Mesoderm induction
  - Endoderm induction
  - Neural induction
  - Signal responsiveness
  - Flow cytometry analysis
  - RT-qPCR
  - Immunofluorescence analysis
  - FISH for Xist
  - Metaphase chromosome analysis
  - Immunoblotting
  - Etv4/5 and Otx2 knock out analysis
  - RNA-sequencing
  - ATAC-seq


**Figure 7. hFS-like Cells Established from Naive ESCs and Embryos**

(A) Morphology of human $A_{lo}$XR cells derived from naive hPSCs. Scale bar, 100 μm.
(B) qRT-PCR expression analysis of marker genes in two human FS (hFS) cell lines compared with naive and conventional (primed) hPSCs. Error bars represent SD from technical triplicates.
(C) SOX17 immunostaining of hFS cells after endoderm induction.
(D) SOX1 immunostaining of hFS cells after neural induction.
(E) qRT-PCR analysis of hFS cells differentiated into paraxial mesoderm for 6 days. Error bars represent SD from technical triplicates.
(F) PCA of hFS cells with naive and conventional hPSCs computed with 11,051 genes identified by median Log2 expression of >0.5.
(G) Projection of hFS cell and conventional PSC samples onto PCA of *Macaca* ICM/epiblast stages computed with 9,432 orthologous expressed genes.
(H) PCA for cell line populations computed using 922 variable genes across epiblast samples from human embryo extended culture (Xiang et al., 2019) with projection of embryo single cells.
(I) Fragments per kilobase of exon model per million reads mapped (FPKM) values for naive-formative specific genes in naive, formative, or conventional hPSCs.
(J) Boxplots of naive-formative specific gene expression in human epiblast stages and primitive streak anlage (PSA).
(K) Heatmap of differentially expressed transposable elements between naive, formative, and conventional samples.
(L) Morphology of FS cells derived directly from human embryo. Scale bar, 100 μm.
(M) Immunostaining of OCT4, SOX2, and NANOG in embryo-derived hFS cells. Scale bar, 250 μm.
(N) qRT-PCR analysis of embryo-derived hFS cells. Error bars represent SD from technical duplicates.

- ChIP-seq
- Single-cell RNA-seq
- Chimeras
- QUANTIFICATION AND STATISTICAL ANALYSIS
  - Bulk RNA-seq analysis
  - Published RNA-seq data comparison analysis
  - PCA plots
  - scRNA-seq analysis
  - GO-terms
  - ATAC-seq
  - BS-seq
  - ChIP-seq

## SUPPLEMENTAL INFORMATION

Supplemental Information can be found online at https://doi.org/10.1016/j.stem.2020.11.005.

## ACKNOWLEDGMENTS

We thank Vicki Metzis for the ATAC-seq protocol, Elsa Sousa for help with *Xist* FISH, and Meng Amy Li for the gRNA expression vector. Tüzer Kalkan, Nicola Reynolds, and Laurence Bates advised on ChIP-seq. We are grateful to Maike Paramor, Vicki Murray, Peter Humphreys, Darran Clements, Andrew Riddell, Charles-Etienne Dumeau, and biofacility staff for technical support and to the CSCI core bioinformatics team for data processing and routine analysis. Sequencing was performed by the CRUK Cambridge Institute Genomics Core Facility. Shahzaib Ahmed and Benjamin Porteous contributed to experiments. Rosalind Drummond and James Clarke provided laboratory assistance. We thank Brian Hendrich for comments on the manuscript. The Cambridge Stem Cell Institute receives core funding from Wellcome and the Medical Research Council. This research was funded by the Biotechnology and Biological Sciences Research Council and the Medical Research Council United Kingdom. A.S. is a Medical Research Council professor.

## AUTHOR CONTRIBUTIONS

Conceptualization, A.S.; Methods, M.K.; Formal Analysis, M.B., D.S., G.G.S., and S.D.; Investigation, M.K., W.M., Y.C., and J.N.; Writing, M.K. and A.S.; Supervision, A.S.

## DECLARATION OF INTERESTS

The authors declare no competing interests

Received: September 6, 2019
Revised: September 3, 2020
Accepted: November 9, 2020
Published: December 2, 2020

## REFERENCES

Acampora, D., Di Giovannantonio, L.G., and Simeone, A. (2013). Otx2 is an intrinsic determinant of the embryonic stem cell state and is required for transition to a stable epiblast stem cell condition. Development *140*, 43–55.

Acampora, D., Omodei, D., Petrosino, G., Garofalo, A., Savarese, M., Nigro, V., Di Giovannantonio, L.G., Mercadante, V., and Simeone, A. (2016). Loss of the Otx2-Binding Site in the Nanog Promoter Affects the Integrity of Embryonic Stem Cell Subtypes and Specification of Inner Cell Mass-Derived Epiblast. Cell Rep. *15*, 2651–2664.

Allison, T.F., Smith, A.J.H., Anastassiadis, K., Sloane-Stanley, J., Biga, V., Stavish, D., Hackland, J., Sabri, S., Langerman, J., Jones, M., et al. (2018). Identification and Single-Cell Functional Characterization of an Endodermally Biased Pluripotent Substate in Human Embryonic Stem Cells. Stem Cell Reports *10*, 1895–1907.

Ang, S.L., Jin, O., Rhinn, M., Daigle, N., Stevenson, L., and Rossant, J. (1996). A targeted mouse Otx2 mutation leads to severe defects in gastrulation and formation of axial mesoderm and to deletion of rostral brain. Development *122*, 243–252.

Auclair, G., Guibert, S., Bender, A., and Weber, M. (2014). Ontogeny of CpG island methylation and specificity of DNMT3 methyltransferases during embryonic development in the mouse. Genome Biol. *15*, 545.

Azuara, V., Perry, P., Sauer, S., Spivakov, M., Jørgensen, H.F., John, R.M., Gouti, M., Casanova, M., Warnes, G., Merkenschlager, M., and Fisher, A.G. (2006). Chromatin signatures of pluripotent cell lines. Nat. Cell Biol. *8*, 532–538.

Bain, G., Kitchens, D., Yao, M., Huettner, J.E., and Gottlieb, D.I. (1995). Embryonic stem cells express neuronal properties in vitro. Dev. Biol. *168*, 342–357.

Beddington, R.S., and Robertson, E.J. (1998). Anterior patterning in mouse. Trends Genet. *14*, 277–284.

Bernstein, B.E., Mikkelsen, T.S., Xie, X., Kamal, M., Huebert, D.J., Cuff, J., Fry, B., Meissner, A., Wernig, M., Plath, K., et al. (2006). A bivalent chromatin structure marks key developmental genes in embryonic stem cells. Cell *125*, 315–326.

Boroviak, T., Loos, R., Bertone, P., Smith, A., and Nichols, J. (2014). The ability of inner-cell-mass cells to self-renew as embryonic stem cells is acquired following epiblast specification. Nat. Cell Biol. *16*, 516–528.

Boroviak, T., Loos, R., Lombard, P., Okahara, J., Behr, R., Sasaki, E., Nichols, J., Smith, A., and Bertone, P. (2015). Lineage-Specific Profiling Delineates the Emergence and Progression of Naive Pluripotency in Mammalian Embryogenesis. Dev. Cell *35*, 366–382.

Bredenkamp, N., Yang, J., Clarke, J., Stirparo, G.G., von Meyenn, F., Dietmann, S., Baker, D., Drummond, R., Ren, Y., Li, D., et al. (2019). Wnt Inhibition Facilitates RNA-Mediated Reprogramming of Human Somatic Cells to Naive Pluripotency. Stem Cell Reports *13*, 1083–1098.

Brennan, J., Lu, C.C., Norris, D.P., Rodriguez, T.A., Beddington, R.S., and Robertson, E.J. (2001). Nodal signalling in the epiblast patterns the early mouse embryo. Nature *411*, 965–969.

Brons, I.G., Smithers, L.E., Trotter, M.W., Rugg-Gunn, P., Sun, B., Chuva de Sousa Lopes, S.M., Howlett, S.K., Clarkson, A., Ahrlund-Richter, L., Pedersen, R.A., and Vallier, L. (2007). Derivation of pluripotent epiblast stem cells from mammalian embryos. Nature *448*, 191–195.

Brook, F.A., and Gardner, R.L. (1997). The origin and efficient derivation of embryonic stem cells in the mouse. Proc. Natl. Acad. Sci. USA *94*, 5709–5712.

Buecker, C., Srinivasan, R., Wu, Z., Calo, E., Acampora, D., Faial, T., Simeone, A., Tan, M., Swigut, T., and Wysocka, J. (2014). Reorganization of enhancer patterns in transition from naive to primed pluripotency. Cell Stem Cell *14*, 838–853.

Buenrostro, J.D., Giresi, P.G., Zaba, L.C., Chang, H.Y., and Greenleaf, W.J. (2013). Transposition of native chromatin for fast and sensitive epigenomic profiling of open chromatin, DNA-binding proteins and nucleosome position. Nat. Methods *10*, 1213–1218.

Burgold, T., Barber, M., Kloet, S., Cramard, J., Gharbi, S., Floyd, R., Kinoshita, M., Ralser, M., Vermeulen, M., Reynolds, N., et al. (2019). The Nucleosome Remodelling and Deacetylation complex suppresses transcriptional noise during lineage commitment. EMBO J. *38*, e100788.

Butler, A., Hoffman, P., Smibert, P., Papalexi, E., and Satija, R. (2018). Integrating single-cell transcriptomic data across different conditions, technologies, and species. Nat. Biotechnol *36*, 411–420.

Chal, J., Al Tanoury, Z., Hestin, M., Gobert, B., Aivio, S., Hick, A., Cherrier, T., Nesmith, A.P., Parker, K.K., and Pourquié, O. (2016). Generation of human muscle fibers and satellite-like cells from human pluripotent stem cells in vitro. Nat. Protoc. *11*, 1833–1850.

Chambers, S.M., Fasano, C.A., Papapetrou, E.P., Tomishima, M., Sadelain, M., and Studer, L. (2009). Highly efficient neural conversion of human ES and iPS cells by dual inhibition of SMAD signaling. Nat. Biotechnol. *27*, 275–280.

Chen, G., Gulbranson, D.R., Hou, Z., Bolin, J.M., Ruotti, V., Probasco, M.D., Smuga-Otto, K., Howden, S.E., Diol, N.R., Propson, N.E., et al. (2011).

Chemically defined conditions for human iPSC derivation and culture. Nat. Methods *8*, 424–429.

Cheng, S., Pei, Y., He, L., Peng, G., Reinius, B., Tam, P.P.L., Jing, N., and Deng, Q. (2019). Single-Cell RNA-Seq Reveals Cellular Heterogeneity of Pluripotency Transition and X Chromosome Dynamics during Early Mouse Development. Cell Rep. *26*, 2593–2607.e3.

Conlon, F.L., Lyons, K.M., Takaesu, N., Barth, K.S., Kispert, A., Herrmann, B., and Robertson, E.J. (1994). A primary requirement for nodal in the formation and maintenance of the primitive streak in the mouse. Development *120*, 1919–1928.

Cornacchia, D., Zhang, C., Zimmer, B., Chung, S.Y., Fan, Y., Soliman, M.A., Tchieu, J., Chambers, S.M., Shah, H., Paull, D., et al. (2019). Lipid Deprivation Induces a Stable, Naive-to-Primed Intermediate State of Pluripotency in Human PSCs. Cell Stem Cell *25*, 120–136.e10.

D'Aniello, C., Habibi, E., Cermola, F., Paris, D., Russo, F., Fiorenzano, A., Di Napoli, G., Melck, D.J., Cobellis, G., Angelini, C., et al. (2016). Vitamin C and l-Proline Antagonistic Effects Capture Alternative States in the Pluripotency Continuum. Stem Cell Reports *8*, 1–10.

Davidson, K.C., Mason, E.A., and Pera, M.F. (2015). The pluripotent state in mouse and human. Development *142*, 3090–3099.

Dobin, A., Davis, C.A., Schlesinger, F., Drenkow, J., Zaleski, C., Jha, S., Batut, P., Chaisson, M., and Gingeras, T.R. (2013). STAR: ultrafast universal RNA-seq aligner. Bioinformatics *29*, 15–21.

Dunn, S.J., Martello, G., Yordanov, B., Emmott, S., and Smith, A.G. (2014). Defining an essential transcription factor program for naïve pluripotency. Science *344*, 1156–1160.

Friedli, M., and Trono, D. (2015). The developmental control of transposable elements and the evolution of higher species. Annu. Rev. Cell Dev. Biol. *31*, 429–451.

Gardner, R.L. (1985). Clonal analysis of early mammalian development. Philos. Trans. R. Soc. Lond. B Biol. Sci. *312*, 163–178.

Gardner, R.L., and Brook, F.A. (1997). Reflections on the biology of embryonic stem (ES) cells. Int. J. Dev. Biol. *41*, 235–243.

Gardner, R.L., Lyon, M.F., Evans, E.P., and Burtenshaw, M.D. (1985). Clonal analysis of X-chromosome inactivation and the origin of the germ line in the mouse embryo. J. Embryol. Exp. Morphol. *88*, 349–363.

Gokhale, P.J., Au-Young, J.K., Dadi, S., Keys, D.N., Harrison, N.J., Jones, M., Soneji, S., Enver, T., Sherlock, J.K., and Andrews, P.W. (2015). Culture adaptation alters transcriptional hierarchies among single human embryonic stem cells reflecting altered patterns of differentiation. PLoS One *10*, e0123467.

Guo, G., Yang, J., Nichols, J., Hall, J.S., Eyres, I., Mansfield, W., and Smith, A. (2009). Klf4 reverts developmentally programmed restriction of ground state pluripotency. Development *136*, 1063–1069.

Guo, G., von Meyenn, F., Santos, F., Chen, Y., Reik, W., Bertone, P., Smith, A., and Nichols, J. (2016). Naive Pluripotent Stem Cells Derived Directly from Isolated Cells of the Human Inner Cell Mass. Stem Cell Reports *6*, 437–446.

Guo, G., von Meyenn, F., Rostovskaya, M., Clarke, J., Dietmann, S., Baker, D., Sahakyan, A., Myers, S., Bertone, P., Reik, W., et al. (2017). Epigenetic resetting of human pluripotency. Development *144*, 2748–2763.

Guzman-Ayala, M., Ben-Haim, N., Beck, S., and Constam, D.B. (2004). Nodal protein processing and fibroblast growth factor 4 synergize to maintain a trophoblast stem cell microenvironment. Proc. Natl. Acad. Sci. USA *101*, 15656–15660.

Hackett, J.A., and Surani, M.A. (2014). Regulatory principles of pluripotency: from the ground state up. Cell Stem Cell *15*, 416–430.

Hafemeister, C., and Satija, R. (2019). Normalization and variance stabilization of single-cell RNA-seq data using regularized negative binomial regression. Genome Biol. *20*, 296.

Han, D.W., Tapia, N., Joo, J.Y., Greber, B., Araúzo-Bravo, M.J., Bernemann, C., Ko, K., Wu, G., Stehling, M., Do, J.T., and Schöler, H.R. (2010). Epiblast stem cell subpopulations represent mouse embryos of distinct pregastrulation stages. Cell *143*, 617–627.

Haub, O., and Goldfarb, M. (1991). Expression of the fibroblast growth factor-5 gene in the mouse embryo. Development *112*, 397–406.

Hayashi, K., Ohta, H., Kurimoto, K., Aramaki, S., and Saitou, M. (2011). Reconstitution of the mouse germ cell specification pathway in culture by pluripotent stem cells. Cell *146*, 519–532.

Heinz, S., Benner, C., Spann, N., Bertolino, E., Lin, Y.C., Laslo, P., Cheng, J.X., Murre, C., Singh, H., and Glass, C.K. (2010). Simple combinations of lineage-determining transcription factors prime cis-regulatory elements required for macrophage and B cell identities. Mol. Cell *38*, 576–589.

Hooper, M.L., Hardy, K., Handyside, A., Hunter, S., and Monk, M. (1987). HPRT-deficient (Lesch-Nyhan) mouse embryos derived from germline colonization by cultured cells. Nature *326*, 292–295.

Hough, S.R., Laslett, A.L., Grimmond, S.B., Kolle, G., and Pera, M.F. (2009). A continuum of cell states spans pluripotency and lineage commitment in human embryonic stem cells. PLoS One *4*, e7708.

Hough, S.R., Thornton, M., Mason, E., Mar, J.C., Wells, C.A., and Pera, M.F. (2014). Single-cell gene expression profiles define self-renewing, pluripotent, and lineage primed states of human pluripotent stem cells. Stem Cell Reports *2*, 881–895.

Huang, D.W., Sherman, B.T., and Lempicki, R.A. (2009). Systematic and integrative analysis of large gene lists using DAVID bioinformatics resources. Nat. Protoc. *4*, 44–57.

Irie, N., Weinberger, L., Tang, W.W., Kobayashi, T., Viukov, S., Manor, Y.S., Dietmann, S., Hanna, J.H., and Surani, M.A. (2015). SOX17 is a critical specifier of human primordial germ cell fate. Cell *160*, 253–268.

Kalkan, T., and Smith, A. (2014). Mapping the route from naive pluripotency to lineage specification. Philos. Trans. R. Soc. Lond. B Biol. Sci. *369*, 20130540.

Kalkan, T., Olova, N., Roode, M., Mulas, C., Lee, H.J., Nett, I., Marks, H., Walker, R., Stunnenberg, H.G., Lilley, K.S., et al. (2017). Tracking the embryonic stem cell transition from ground state pluripotency. Development *144*, 1221–1234.

Kalkan, T., Bornelöv, S., Mulas, C., Diamanti, E., Lohoff, T., Ralser, M., Middelkamp, S., Lombard, P., Nichols, J., and Smith, A. (2019). Complementary Activity of ETV5, RBPJ, and TCF3 Drives Formative Transition from Naive Pluripotency. Cell Stem Cell *24*, 785–801.e7.

Kim, D., Pertea, G., Trapnell, C., Pimentel, H., Kelley, R., and Salzberg, S.L. (2013). TopHat2: accurate alignment of transcriptomes in the presence of insertions, deletions and gene fusions. Genome Biol. *14*, R36.

Kinoshita, M., and Smith, A. (2018). Pluripotency Deconstructed. Dev. Growth Differ. *60*, 44–52.

Kojima, Y., Kaufman-Francis, K., Studdert, J.B., Steiner, K.A., Power, M.D., Loebel, D.A., Jones, V., Hor, A., de Alencastro, G., Logan, G.J., et al. (2014). The transcriptional and functional properties of mouse epiblast stem cells resemble the anterior primitive streak. Cell Stem Cell *14*, 107–120.

Krueger, F., and Andrews, S.R. (2011). Bismark: a flexible aligner and methylation caller for Bisulfite-Seq applications. Bioinformatics *27*, 1571–1572.

Kunath, T., Saba-El-Leil, M.K., Almousailleakh, M., Wray, J., Meloche, S., and Smith, A. (2007). FGF stimulation of the Erk1/2 signalling cascade triggers transition of pluripotent embryonic stem cells from self-renewal to lineage commitment. Development *134*, 2895–2902.

Kurek, D., Neagu, A., Tastemel, M., Tüysüz, N., Lehmann, J., van de Werken, H.J.G., Philipsen, S., van der Linden, R., Maas, A., van IJcken, W.F.J., et al. (2015). Endogenous WNT signals mediate BMP-induced and spontaneous differentiation of epiblast stem cells and human embryonic stem cells. Stem Cell Reports *4*, 114–128.

Langmead, B., and Salzberg, S.L. (2012). Fast gapped-read alignment with Bowtie 2. Nat. Meth. *9*, 357–359.

Lau, K.X., Mason, E.A., Kie, J., De Souza, D.P., Kloehn, J., Tull, D., McConville, M.J., Keniry, A., Beck, T., Blewitt, M.E., et al. (2020). Unique properties of a subset of human pluripotent stem cells with high capacity for self-renewal. Nat. Commun. *11*, 2420.

Lawson, K.A., Meneses, J.J., and Pedersen, R.A. (1991). Clonal analysis of epiblast fate during germ layer formation in the mouse embryo. Development *113*, 891–911.

Liao, Y., Smyth, G.K., and Shi, W. (2019). The R package Rsubread is easier, faster, cheaper and better for alignment and quantification of RNA sequencing reads. Nucleic Acids Res. *47*, e47.

Liu, P., Wakamiya, M., Shea, M.J., Albrecht, U., Behringer, R.R., and Bradley, A. (1999). Requirement for Wnt3 in vertebrate axis formation. Nat. Genet. *22*, 361–365.

Loh, K.M., Ang, L.T., Zhang, J., Kumar, V., Ang, J., Auyeong, J.Q., Lee, K.L., Choo, S.H., Lim, C.Y., Nichane, M., et al. (2014). Efficient endoderm induction from human pluripotent stem cells by logically directing signals controlling lineage bifurcations. Cell Stem Cell *14*, 237–252.

Love, M.I., Huber, W., and Anders, S. (2014). Moderated estimation of fold change and dispersion for RNA-seq data with DESeq2. Genome Biol. *15*, 550.

Lu, B.C., Cebrian, C., Chi, X., Kuure, S., Kuo, R., Bates, C.M., Arber, S., Hassell, J., MacNeil, L., Hoshi, M., et al. (2009). Etv4 and Etv5 are required downstream of GDNF and Ret for kidney branching morphogenesis. Nat. Genet. *41*, 1295–1302.

Mak, W., Nesterova, T.B., de Napoles, M., Appanah, R., Yamanaka, S., Otte, A.P., and Brockdorff, N. (2004). Reactivation of the paternal X chromosome in early mouse embryos. Science *303*, 666–669.

Masaki, H., Kato-Itoh, M., Takahashi, Y., Umino, A., Sato, H., Ito, K., Yanagida, A., Nishimura, T., Yamaguchi, T., Hirabayashi, M., et al. (2016). Inhibition of Apoptosis Overcomes Stage-Related Compatibility Barriers to Chimera Formation in Mouse Embryos. Cell Stem Cell *19*, 587–592.

Mesnard, D., Guzman-Ayala, M., and Constam, D.B. (2006). Nodal specifies embryonic visceral endoderm and sustains pluripotent cells in the epiblast before overt axial patterning. Development *133*, 2497–2505.

Mulas, C., Kalkan, T., and Smith, A. (2017). NODAL Secures Pluripotency upon Embryonic Stem Cell Progression from the Ground State. Stem Cell Reports *9*, 77–91.

Mulas, C., Kalkan, T., von Meyenn, F., Leitch, H.G., Nichols, J., and Smith, A. (2019). Defined conditions for propagation and manipulation of mouse embryonic stem cells. Development *146*, dev173146.

Murakami, K., Günesdogan, U., Zylicz, J.J., Tang, W.W.C., Sengupta, R., Kobayashi, T., Kim, S., Butler, R., Dietmann, S., and Surani, M.A. (2016). NANOG alone induces germ cells in primed epiblast in vitro by activation of enhancers. Nature *529*, 403–407.

Najm, F.J., Chenoweth, J.G., Anderson, P.D., Nadeau, J.H., Redline, R.W., McKay, R.D., and Tesar, P.J. (2011). Isolation of epiblast stem cells from preimplantation mouse embryos. Cell Stem Cell *8*, 318–325.

Nakaki, F., Hayashi, K., Ohta, H., Kurimoto, K., Yabuta, Y., and Saitou, M. (2013). Induction of mouse germ-cell fate by transcription factors in vitro. Nature *501*, 222–226.

Nakamura, T., Okamoto, I., Sasaki, K., Yabuta, Y., Iwatani, C., Tsuchiya, H., Seita, Y., Nakamura, S., Yamamoto, T., and Saitou, M. (2016). A developmental coordinate of pluripotency among mice, monkeys and humans. Nature *537*, 57–62.

Nakanishi, M., Mitchell, R.R., Benoit, Y.D., Orlando, L., Reid, J.C., Shimada, K., Davidson, K.C., Shapovalova, Z., Collins, T.J., Nagy, A., and Bhatia, M. (2019). Human Pluripotency Is Initiated and Preserved by a Unique Subset of Founder Cells. Cell *177*, 910–924.e22.

Neagu, A., van Genderen, E., Escudero, I., Verwegen, L., Kurek, D., Lehmann, J., Stel, J., Dirks, R.A.M., van Mierlo, G., Maas, A., et al. (2020). In vitro capture and characterization of embryonic rosette-stage pluripotency between naive and primed states. Nat. Cell Biol. *22*, 534–545.

Nichols, J., and Smith, A. (2009). Naive and primed pluripotent states. Cell Stem Cell *4*, 487–492.

Nichols, J., and Ying, Q.L. (2006). Derivation and propagation of embryonic stem cells in serum- and feeder-free culture. Methods Mol. Biol. *329*, 91–98.

Niswander, L., and Martin, G.R. (1992). Fgf-4 expression during gastrulation, myogenesis, limb and tooth development in the mouse. Development *114*, 755–768.

O'Leary, T., Heindryckx, B., Lierman, S., van Bruggen, D., Goeman, J.J., Vandewoestyne, M., Deforce, D., de Sousa Lopes, S.M., and De Sutter, P. (2012). Tracking the progression of the human inner cell mass during embryonic stem cell derivation. Nat. Biotechnol. *30*, 278–282.

Ohinata, Y., Payer, B., O'Carroll, D., Ancelin, K., Ono, Y., Sano, M., Barton, S.C., Obukhanych, T., Nussenzweig, M., Tarakhovsky, A., et al. (2005). Blimp1 is a critical determinant of the germ cell lineage in mice. Nature *436*, 207–213.

Ohinata, Y., Ohta, H., Shigeta, M., Yamanaka, K., Wakayama, T., and Saitou, M. (2009). A signaling principle for the specification of the germ cell lineage in mice. Cell *137*, 571–584.

Ohtsuka, S., Nishikawa-Torikai, S., and Niwa, H. (2012). E-cadherin promotes incorporation of mouse epiblast stem cells into normal development. PLoS One *7*, e45220.

Osorno, R., Tsakiridis, A., Wong, F., Cambray, N., Economou, C., Wilkie, R., Blin, G., Scotting, P.J., Chambers, I., and Wilson, V. (2012). The developmental dismantling of pluripotency is reversed by ectopic Oct4 expression. Development *139*, 2288–2298.

Osteil, P., Studdert, J.B., Goh, H.N., Wilkie, E.E., Fan, X., Khoo, P.-L., Peng, G., Salehin, N., Knowles, H., Han, J.J., et al. (2019). Dynamics of Wnt activity on the acquisition of ectoderm potency in epiblast stem cells. Development *146*, dev172858.

Peng, G., Suo, S., Chen, J., Chen, W., Liu, C., Yu, F., Wang, R., Chen, S., Sun, N., Cui, G., et al. (2016). Spatial Transcriptome for the Molecular Annotation of Lineage Fates and Cell Identity in Mid-gastrula Mouse Embryo. Dev. Cell *36*, 681–697.

Peng, G., Suo, S., Cui, G., Yu, F., Wang, R., Chen, J., Chen, S., Liu, Z., Chen, G., Qian, Y., et al. (2019). Molecular architecture of lineage allocation and tissue organization in early mouse embryo. Nature *572*, 528–532.

Picelli, S., Faridani, O.R., Björklund, A.K., Winberg, G., Sagasser, S., and Sandberg, R. (2014). Full-length RNA-seq from single cells using Smart-seq2. Nat. Protoc. *9*, 171–181.

Ramirez, F., Ryan, D.P., Gruning, B., Bhardwaj, V., Kilpert, F., Richter, A.S., Heyne, S., Dundar, F., and Manke, T. (2016). deepTools2: a next generation web server for deep-sequencing data analysis. Nucleic Acids Res. *44*, W160–W165.

Rathjen, J., Lake, J.A., Bettess, M.D., Washington, J.M., Chapman, G., and Rathjen, P.D. (1999). Formation of a primitive ectoderm like cell population, EPL cells, from ES cells in response to biologically derived factors. J. Cell Sci. *112*, 601–612.

Robertson, E.J. (2014). Dose-dependent Nodal/Smad signals pattern the early mouse embryo. Semin. Cell Dev. Biol. *32*, 73–79.

Rossant, J. (2015). Mouse and human blastocyst-derived stem cells: vive les differences. Development *142*, 9–12.

Rossant, J., and Tam, P.P.L. (2017). New Insights into Early Human Development: Lessons for Stem Cell Derivation and Differentiation. Cell Stem Cell *20*, 18–28.

Rostovskaya, M., Stirparo, G.G., and Smith, A. (2019). Capacitation of human naïve pluripotent stem cells for multi-lineage differentiation. Development *146*, dev172916.

Sasaki, K., Yokobayashi, S., Nakamura, T., Okamoto, I., Yabuta, Y., Kurimoto, K., Ohta, H., Moritoki, Y., Iwatani, C., Tsuchiya, H., et al. (2015). Robust In Vitro Induction of Human Germ Cell Fate from Pluripotent Stem Cells. Cell Stem Cell *17*, 178–194.

Shiura, H., and Abe, K. (2019). Xist/Tsix expression dynamics during mouse peri-implantation development revealed by whole-mount 3D RNA-FISH. Sci. Rep. *9*, 3637.

Smith, A. (2017). Formative pluripotency: the executive phase in a developmental continuum. Development *144*, 365–373.

Smith, A.G., Heath, J.K., Donaldson, D.D., Wong, G.G., Moreau, J., Stahl, M., and Rogers, D. (1988). Inhibition of pluripotential embryonic stem cell differentiation by purified polypeptides. Nature *336*, 688–690.

Solter, D., and Knowles, B.B. (1975). Immunosurgery of mouse blastocyst. Proc. Natl. Acad. Sci. USA *72*, 5099–5102.

Song, Q., Decato, B., Hong, E.E., Zhou, M., Fang, F., Qu, J., Garvin, T., Kessler, M., Zhou, J., and Smith, A.D. (2013). A reference methylome database

CellPress
OPEN ACCESS

and analysis pipeline to facilitate integrative and comparative epigenomics. PLoS One *8*, e81148.

Sousa, E.J., Stuart, H.T., Bates, L.E., Ghorbani, M., Nichols, J., Dietmann, S., and Silva, J.C.R. (2018). Exit from Naive Pluripotency Induces a Transient X Chromosome Inactivation-like State in Males. Cell Stem Cell *22*, 919–928.e916.

Stavridis, M.P., and Smith, A.G. (2003). Neural differentiation of mouse embryonic stem cells. Biochem. Soc. Trans. *31*, 45–49.

Stavridis, M.P., Lunn, J.S., Collins, B.J., and Storey, K.G. (2007). A discrete period of FGF-induced Erk1/2 signalling is required for vertebrate neural specification. Development *134*, 2889–2894.

Stavridis, M.P., Collins, B.J., and Storey, K.G. (2010). Retinoic acid orchestrates fibroblast growth factor signalling to drive embryonic stem cell differentiation. Development *137*, 881–890.

Strawbridge, S.E., Blanchard, G.B., Smith, A., Kugler, H., and Martello, G. (2020). Embryonic stem cells commit to differentiation by symmetric divisions following a variable lag period. bioRxiv, 2020.2006.2017.157578.

Sun, X., Meyers, E.N., Lewandoski, M., and Martin, G.R. (1999). Targeted disruption of Fgf8 causes failure of cell migration in the gastrulating mouse embryo. Genes Dev. *13*, 1834–1846.

Takashima, Y., Guo, G., Loos, R., Nichols, J., Ficz, G., Krueger, F., Oxley, D., Santos, F., Clarke, J., Mansfield, W., et al. (2014). Resetting transcription factor control circuitry toward ground-state pluripotency in human. Cell *158*, 1254–1269.

Takenaga, M., Fukumoto, M., and Hori, Y. (2007). Regulated Nodal signaling promotes differentiation of the definitive endoderm and mesoderm from ES cells. J. Cell Sci. *120*, 2078–2090.

Tesar, P.J., Chenoweth, J.G., Brook, F.A., Davies, T.J., Evans, E.P., Mack, D.L., Gardner, R.L., and McKay, R.D. (2007). New cell lines from mouse epiblast share defining features with human embryonic stem cells. Nature *448*, 196–199.

Theunissen, T.W., Friedli, M., He, Y., Planet, E., O'Neil, R.C., Markoulaki, S., Pontis, J., Wang, H., Iouranova, A., Imbeault, M., et al. (2016). Molecular Criteria for Defining the Naive Human Pluripotent State. Cell Stem Cell *19*, 502–515.

Tsakiridis, A., Huang, Y., Blin, G., Skylaki, S., Wymeersch, F., Osorno, R., Economou, C., Karagianni, E., Zhao, S., Lowell, S., and Wilson, V. (2014). Distinct Wnt-driven primitive streak-like populations reflect in vivo lineage precursors. Development *141*, 1209–1221.

Varlet, I., Collignon, J., and Robertson, E.J. (1997). nodal expression in the primitive endoderm is required for specification of the anterior axis during mouse gastrulation. Development *124*, 1033–1044.

Watanabe, K., Ueno, M., Kamiya, D., Nishiyama, A., Matsumura, M., Wataya, T., Takahashi, J.B., Nishikawa, S., Nishikawa, S., Muguruma, K., and Sasai, Y. (2007). A ROCK inhibitor permits survival of dissociated human embryonic stem cells. Nat. Biotechnol. *25*, 681–686.

Williams, R.L., Hilton, D.J., Pease, S., Willson, T.A., Stewart, C.L., Gearing, D.P., Wagner, E.F., Metcalf, D., Nicola, N.A., and Gough, N.M. (1988). Myeloid leukaemia inhibitory factor maintains the developmental potential of embryonic stem cells. Nature *336*, 684–687.

Winnier, G., Blessing, M., Labosky, P.A., and Hogan, B.L. (1995). Bone morphogenetic protein-4 is required for mesoderm formation and patterning in the mouse. Genes Dev. *9*, 2105–2116.

Xiang, L., Yin, Y., Zheng, Y., Ma, Y., Li, Y., Zhao, Z., Guo, J., Ai, Z., Niu, Y., Duan, K., et al. (2019). A developmental landscape of 3D-cultured human pre-gastrulation embryos. Nature *577*, 537–542.

Yang, S.H., Kalkan, T., Morissroe, C., Marks, H., Stunnenberg, H., Smith, A., and Sharrocks, A.D. (2014). Otx2 and Oct4 drive early enhancer activation during embryonic stem cell transition from naive pluripotency. Cell Rep. *7*, 1968–1981.

Zerbino, D.R., Johnson, N., Juettemann, T., Wilder, S.P., and Flicek, P. (2014). WiggleTools: parallel processing of large collections of genome-wide datasets for visualization and statistical analysis. Bioinformatics *30*, 1008–1009.

Zhang, Y., Liu, T., Meyer, C.A., Eeckhoute, J., Johnson, D.S., Bernstein, B.E., Nusbaum, C., Myers, R.M., Brown, M., Li, W., et al. (2008). Model-based analysis of ChIP-Seq (MACS). Genome Biol *9*, R137.

Zhang, Z., Verheyden, J.M., Hassell, J.A., and Sun, X. (2009). FGF-regulated Etv genes are essential for repressing Shh expression in mouse limb buds. Dev. Cell *16*, 607–613.

Zylicz, J.J., Dietmann, S., Günesdogan, U., Hackett, J.A., Cougot, D., Lee, C., and Surani, M.A. (2015). Chromatin dynamics and the role of G9a in gene regulation and enhancer silencing during early mouse development. eLife *4*, e09571.

# STAR★METHODS

## KEY RESOURCES TABLE

| REAGENT or RESOURCE | SOURCE | IDENTIFIER |
|---|---|---|
| Antibodies | | |
| Mouse monoclonal anti-Oct3/4 (C-10) | Santa Cruz | Cat#SC-5279; RRID:AB_628051 |
| Goat polyclonal anti-Oct3/4 (N-19) | Santa Cruz | Cat#SC-8628; RRID:AB_653551 |
| Goat polyclonal anti-Brachyury | R&D systems | Cat#AF2085; RRID:AB_2200235 |
| Rabbit polyclonal anti-Sox1 | Cell Signaling Technology | Cat#4194; RRID:AB_1904140 |
| Rabbit polyclonal anti-Stella/Dppa3 | Abcam | Cat#ab19878; RRID:AB_2246120 |
| Rat monoclonal anti-Blimp1/Prdm1 | Santa Cruz | Cat#SC-47732; RRID:AB_628168 |
| Mouse monoclonal anti-Foxa2 | Abnova | Cat#H00003170-M10; RRID:AB_534871 |
| Mouse monoclonal anti-Tuj1 | R&D systems | Cat#MAB1195; RRID:AB_357520 |
| Mouse anti-cardiac Troponin T (1C11) | Abcam | Cat#Ab8295; RRID:AB_306445 |
| Goat polyclonal anti-Sox17 | R&D systems | Cat#AF1924; RRID:AB_355060 |
| Goat polyclonal anti-Gata4 | Santa Cruz | Cat#SC-1237; RRID:AB_2108747 |
| Rabbit polyclonal anti-Eomes | Abcam | Cat#ab23345; RRID:AB_778267 |
| Rat monoclonal anti-Ecadherin (ECCD2) | Kind gift from Prof. M Takeichi | N/A |
| Rat monoclonal anti-Nanog | eBioscience | Cat#14-5761-80; RRID:AB_763613 |
| Rat monoclonal anti-Sox2 | eBioscience | Cat#14-9811-82; RRID:AB_11219471 |
| Mouse monoclonal anti-Oct6 (Pou3f1) | Miilipore | Cat#MABN738; RRID:AB_2876862 |
| Rabbit polyclonal anti-mKusabira Orange | MBL | Cat#PM051M; RRID:AB_2876863 |
| Alexa Fluore 647 anti-SSEA1 | BD Bioscience | Cat#562277; RRID:AB_11154583 |
| PE Anti-mouse/rat CD61 | Biolegend | Cat#104307; RRID:AB_313084 |
| Anti-CD324 (Ecadherin) eFluor-660 | eBioscience | Cat#50-3249-82; RRID:AB_11040003 |
| PE-Cy7 Anti-Ecadherin | Biolegend | Cat#147310; RRID:AB_2564188 |
| APC Anti-mouse CD184 (Cxcr4) | Biolegend | Cat#146508; RRID:AB_2562785 |
| PE Anti-Flk1 | Biolegend | Cat#136403; RRID:AB_1967093 |
| Rabbit anti-RFP | Rockland | Cat#600-401-379; RRID:AB_2209751 |
| Rabbit anti-mvh | Abcam | Cat#ab13840; RRID:AB443012 |
| Rabbit anti-phospho Smad2 | Cell Signaling Technologies | Cat#3108; RRID:AB_490941 |
| Mouse anti-total Smad2/3 | BD Bioscience | Cat#610842; RRID:AB_398161 |
| Mouse anti-Gapdh | Sigma-Aldrich | Cat#G8795; RRID:AB_1078991 |
| Rabbit anti-H3K4me1 | Abcam | Cat#ab8895; RRID:AB_306847 |
| Rabbit anti-H3K4me3 | Diagenode | Cat#C15410003; RRID:AB_2616052 |
| Rabbit anti-H3K27Ac | Active Motif | Cat#39135; RRID:AB_2614979 |
| Rabbit anti-H3K27me3 | Merck | Cat#07-449; RRID:AB_310624 |
| Chemicals, Peptides, and Recombinant Proteins | | |
| XAV939 | Sigma Aldrich | X-3004 |
| BMS493 | Tocris Bio-Techne | 3509 |
| A83-01 | Generon | A12358-50 |
| SB-505124 | Selleckchem | S2186 |
| LDN193189 | Axon Medchem | Axon 1509 |
| PD0325901 | abcr | AB 253775 |
| CHIR99021 | abcr | AB 253776 |
| Y27632 | Millipore | Cat 688000 |
| Recombinant Mouse LIF | In house | N/A |
| Recombinant human LIF | In House | N/A |
| Recombinant human activin A | Qkine | Qk005 |

(*Continued on next page*)

*Continued*

| REAGENT or RESOURCE | SOURCE | IDENTIFIER |
|---|---|---|
| Recombinant zebrafish Fgf2 | Qkine | Qk002 |
| Recombinant mouse Stem Cell Factor | BioLegend | 579706 |
| Recombinant human BMP2 | In House | N/A |
| N2 Supplement | In house | N/A |
| B27 Supplement | Thermo Fisher Scientific | 17504044 |
| Neurobasal | Thermo Fisher Scientific | 11540566 |
| DMEM/F12 | Thermo Fisher Scientific | 21103049 |
| Human Plasma Fibronectin | Millipore | FC010 |
| Tissue culture Laminin | Millipore | CC095-5MG |
| Gelatin | Sigma-Aldrich | G-1890 |
| Accutase | Biolegend | 423201 |
| M2 medium | Sigma-Aldrich | M-7167 |
| Critical Commercial Assays | | |
| NEXTflex Rapid Directional RNA-seq Kit | Bioo Scientific | 5138-08 |
| Ribo-Zero rRNA Removal Kit | Illumina | MRZH11124 |
| PureLink RNA Mini kit | Thermo Fisher Scientific | 12183018A |
| PicoPure RNA Isolation kit | Thermo Fisher Scientific | KIT0214 |
| SMARTerR Stranded Total RNA-Seq Kit v2 – Pico InputMammalian | Takara Clontech | 634412 |
| Nextera DNA Library Preparation Kit | Illumina | FC-121-1030 |
| SAGE Warming Kit | CooperSurgical Fertility & Genomic Solutions | ART-8030 |
| NEXTflex Rapid DNA-Seq Kit 2.0 bundle with 96 HT barcodes | PerkinElmer | NOVA-5188-13 |
| Mouse Xist Stellaris RNA FISH Probe with Quasar 670 Dye | BioSearch Technologies | VSMF-3095-5 |
| 10 CIRCLE, 7MM ID, FROSTED, HEAVY TEFLON COATED Slide | Roboz Surgical Instrument | F107-HTC |
| TransIT LT1 | Mirus | MIR2304 |
| Alkaline Phaphatase Kit | Sigma Aldrich | 86R-1KIT |
| Deposited Data | | |
| RNA-seq | This paper | GEO: GSE131566 |
| ATAC-seq | This paper | GEO: GSE131566 |
| scRNA-seq | This paper | GEO: GSE156589 |
| ChIP-seq | This paper | GEO: GSE156261 |
| Experimental Models: Cell Lines | | |
| 5ar1 (mFS) | This paper | N/A |
| 5ar2 (mFS) | This paper | N/A |
| 5ar3 (mFS) | This paper | N/A |
| 5ar5 (mFS) | This paper | N/A |
| 5cdr1 (mFS) | This paper | N/A |
| 5cdr2 (mFS) | This paper | N/A |
| NBRA3.2 (mFS) | This paper | N/A |
| 5a6 (mFS) | This paper | N/A |
| E14Tg2a (mES) | Hooper et al., 1987 | N/A |
| Rd2 (mES) | Kalkan et al., 2017 | N/A |
| Sox1::GFP (mES) | Stavridis and Smith, 2003 | N/A |
| AFX6 (mEpiSC) | This paper | N/A |
| AFX33 (mEpiSC) | This paper | N/A |

(*Continued on next page*)

*Continued*

| REAGENT or RESOURCE | SOURCE | IDENTIFIER |
|---|---|---|
| AF32 (mEpiSC) | This paper | N/A |
| OEC2 (mEpiSC) | Guo et al., 2009 | N/A |
| HNES1 (hES) | Guo et al., 2016 | N/A |
| cR-H9 (hES) | Guo et al., 2017 | N/A |
| cR-Shef6 (hES) | Guo et al., 2017 | N/A |
| Etv4/5 dKO ES | This paper | N/A |
| Otx2 KO ES | This paper | N/A |
| hFS1 | This paper | N/A |
| hFS2 | This paper | N/A |
| hFS3 | This paper | N/A |
| Experimental Models: Organisms/Strains | | |
| Mouse/CD-1 | Charles River | 022 |
| Mouse/129aa | WT-Gurdon Institute | N/A |
| Mouse/ ROSA$^{mT/mG}$ | Jackson Laboratory | 007576 |
| Mouse/C57BL/6 | WT-Gurdon Institute | N/A |
| Oligonucleotides | | |
| gRNA sequences | See Table S3 | N/A |
| Genotyping primers | See Table S3 | N/A |
| Taqman probes and UPL primers for qRT-PCR | See Table S3 | N/A |
| Recombinant DNA | | |
| pPBCAG-mKO2-IP | This paper | N/A |
| pPBCAG-GFP-IP | This paper | N/A |
| pPBCAG-Cas9-IN | This paper | N/A |
| pCML32 | This paper | N/A |
| Software and Algorithms | | |
| Tophat2 v2.1.0 | Kim et al., 2013 | https://ccb.jhu.edu/software/tophat/index.shtml |
| TrimGalore v0.4.5 | N/A | https://www.bioinformatics.babraham.ac.uk/projects/trim_galore/ |
| FeatureCounts v1.5.0 | Liao et al., 2019 | http://subread.sourceforge.net/ |
| R v3.6.2 | N/A | https://www.R-project.org/ |
| DESeq2 v1.18.1 | Love et al., 2014 | https://bioconductor.org/packages/release/bioc/html/DESeq2.html |
| Pheatmap | N/A | https://cran.r-project.org/web/packages/pheatmap/index.html |
| ggplot2 | N/A | https://ggplot2.tidyverse.org/ |
| DeepTools | Ramirez et al., 2016 | https://doi.org/10.1093/nar/gkw257 |
| Diffbind v2.6.6 | N/A | https://bioconductor.org/packages/release/bioc/html/DiffBind.html |
| MACS2 | Zhang et al., 2008 | N/A |
| DAVID v6.8 | Huang et al., 2009 | https://david.ncifcrf.gov/ |
| HOMER v4.10 | Heinz et al., 2010 | http://homer.ucsd.edu/homer/ |
| Bismark | Krueger and Andrews, 2011 | https://www.bioinformatics.babraham.ac.uk/projects/bismark/ |
| MarkDuplicates | Picard tools | N/A |
| Seurat v3.1.0 | Butler et al., 2018 | https://satijalab.org/seurat/ |
| STAR v2.7.3a | Dobin et al., 2013 | https://github.com/alexdobin/STAR |
| Wiggletools | Zerbino et al., 2014 | https://github.com/Ensembl/WiggleTools |
| Bowtie | Langmead and Salzberg, 2012 | N/A |

(*Continued on next page*)

*Continued*

| REAGENT or RESOURCE | SOURCE | IDENTIFIER |
|---|---|---|
| Samtools v1.9 | N/A | http://www.htslib.org/ |
| FastQC v0.11.3 | N/A | https://www.bioinformatics.babraham.ac.uk/projects/fastqc/ |
| MultiQC v1.8 | N/A | https://multiqc.info/ |
| Methpipe | Song et al., 2013 | http://smithlabresearch.org/software/methpipe/ |
| Venny 2.1 | N/A | https://bioinfogp.cnb.csic.es/tools/venny/index.html |
| FCS Express 7 Research | N/A | De Novo Software |

## RESOURCE AVAILABILITY

### Lead Contact

Further information and requests for resources and reagents should be directed to and will be fulfilled by the Lead Contact, Austin Smith (austin.smith@exeter.ac.uk).

### Materials Availability

All stable reagents generated in this study are available from the Lead Contact without restriction except for human embryo derived cell lines for which permission must be requested from UK Stem Cell Steering Committee and a Materials Transfer Agreement completed.

### Data and Code Availability

The datasets reported in this paper are deposited in Gene Expression Omnibus (GEO) with the following accession codes: RNA-seq and ATAC-seq, GEO: GSE131556; scRNA-seq, GEO: GSE156589; ChIP-seq, GEO: GSE156261

## EXPERIMENTAL MODEL AND SUBJECT DETAILS

### Mice

Mice used in these studies were adult females. CD1 and 129aa strains provided embryos for cell line derivation and ROSA$^{mT/mG}$ mice provided donor embryos for primary epiblast injections. Host embryos for chimera generation were from C57BL/6. CBA/BL6 F1 animals were used as transfer recipients. Animals in the facility tested positive for *Helicobacter* and negative for other specific pathogens. Studies were carried out in a UK Home Office designated facility in accordance with EU guidelines for the care and use of laboratory animals, and under authority of UK Home Office project license 76777883. Use of animals in this project was approved by the Animal Welfare and Ethical Review Body for the University of Cambridge.

### Human Embryos

Supernumerary frozen human embryos were donated with informed consent by couples undergoing *in vitro* fertility treatment. Use of human embryos in this research is approved by the Multi-Centre Research Ethics Committee, approval O4/MRE03/44, and licensed by the Human Embryology & Fertilization Authority of the United Kingdom, research license R0178.

### Cell Cultures

Cell lines are listed in the Key Resources Table. Cell lines were cultured without antibiotics in humidified incubators at 37°C in 7% $CO_2$. Reduced oxygen (5%) was used except for mouse ES cells, which were maintained in atmospheric oxygen. Cell lines tested negative for mycoplasma by periodic PCR screening.

### Mouse FS cell, EpiSC and ES cell culture

FS cells were cultured in $A_{lo}$XR medium, comprising 3ng/ml of activin A, 2μM XAV939 and 1.0μM BMS493 in N2B27 medium (Nichols and Ying, 2006). EpiSCs were cultured in either AF (20ng/ml activin A and 12.5ng/ml Fgf2) or AFX (20ng/ml activin A, 12.5ng/ml Fgf2 and 2μM XAV939) in N2B27 medium. When passaging, cells were dissociated by Accutase into clumps and re-plated every 2-3 days at a ratio of 1:10-1:20. Mouse ES cells were maintained in 2i/LIF medium as described (Mulas et al., 2019). FS cells and EpiSCs were maintained on fibronectin (Fn) coated (16.7 μg/ml) plates. Experiments were generally performed between p10 and p30.

### Derivation of FS and EpiSCs from mouse embryo

E5.5 mouse embryos were dissected from decidua and further micro-dissected into embryonic and extraembryonic parts. Extra-embryonic endoderm layers were removed by mouth pipette and individual epiblasts were plated onto Fn coated (16.7 μg/ml) 4-well

plates in either FS or EpiSC medium. After the epiblast outgrowth became large enough, the outgrowth was briefly incubated in Accutase and collected in wash buffer and re-plated onto a fresh 4-well plate.

### Derivation of FS and EpiSCs from mouse ES cells

ES cells were plated either directly in $A_{lo}$XR, AF or AFX medium or N2B27 basal medium for two days and then re-plated in $A_{lo}$XR, AF or AFX medium. Cultures were passaged at higher densities for the first 4-5 passages with Accutase.

### Derivation of human FS cells from naive PSCs

Human naive PSC propagated in PXGL (Bredenkamp et al., 2019) were cultured in N2B27 medium for 7 days before changing to $A_{lo}$XR. Cells were passaged every 3-5 days at a ratio of 1:10-1:20 and Rock inhibitor was added for the first 24 hours after dissociation. hFS cells were cultured on plates pre-coated with Laminin (10 μg/ml) and Fn (16.7 μg/ml).

### Derivation of human FS cell from embryos

Day 5 or day 6 human embryos were thawed using SAGE REF ART 8030 vitrification warming kit as per the manufacturer's instructions and cultured for one or two days in N2B27 basal medium in 7% $CO_2$ and 5% $O_2$ at 37°C. ICMs were isolated on the following day by immunosurgery (Solter and Knowles, 1975) or mechanical dissociation and plated in $A_{lo}$XR in the presence of Rock inhibitor on laminin/Fn coated 4-well plates. 2-4 weeks later, outgrowths were mechanically dissociated into clumps and replated into a fresh well. After this initial passage, Accutase was used for routine passaging.

## METHOD DETAILS

### Embryoid body differentiation

2,000 cells were plated in low-binding 96-well plates in GMEM supplemented with 10% fetal calf serum, 2 mM L-glutamine, 0.1mM Non-essential Amino Acid (NEAA) (GIBCO), 1mM Sodium Pyruvate and 0.1mM 2-ME. After 5 days, the EBs were transferred for outgrowth onto gelatin-coated plates in fresh medium.

### PGCLC differentiation

3,000 cells were plated in low-binding 96-well plates in GK15 medium (GMEM and 15% Knockout Serum Replacement (GIBCO), 0.1 mM NEAA (GIBCO), 1mM Sodium Pyruvate, 2mM L-Glutamine, 0.1mM 2-mercaptoethanol) supplemented with 500 ng/ml BMP2, 100ng/ml mSCF, 1μg/ml hLIF, 50ng/ml EGF in the presence of 10μM Rho-associated kinase inhibitor Y27632.

### Mesoderm induction

Mouse FS cells were plated with 20ng/ml activin A and 3μM CH in N2B27 for 48 hours on Fn coated plates. Human FS cells were plated with 3μM CHIR99021 and 500 nM LDN193189 for the first 2 days followed by the addition of 20ng/ml of Fgf2 from day 3 to day 6.

### Endoderm induction

Mouse FS cells were plated with 20ng/ml activin A and 3 μM CH in N2B27 for 24 hours and the medium was replaced thereafter with 20ng/ml of activin A only for a further 2 days on Fn coated plate. Human FS cells were differentiated in 100ng/ml activin A, 100nM PI-103, 3μM CH, 10ng/ml Fgf2, 3ng/ml BMP4 and 10μg/ml Heparin for the first 24hrs and then replaced with 100ng/ml activin A, 100nM PI-103, 20ng/ml Fgf2, 250nM LDN193189 and 10 μg/ml Heparin for a further 2 days.

### Neural induction

Mouse FS cells were plated on laminin coated plates in N2B27 (Mulas et al., 2019). Human FS cells were plated with 1μM A83-01 and 500nM LDN193189.

### Signal responsiveness

Cells were plated in self-renewal medium and cultured overnight. On the following day, medium was changed to N2B27 medium with or without growth factors/inhibitors. The concentrations used were, activin A (20 ng/ml), Fgf2 (12.5 ng/ml), CHIR99021 (CH, 3μM), Bmp2 (10 ng/ml), XAV939 (2 μM).

### Flow cytometry analysis

Mouse endoderm and mesoderm cells were dissociated with Cell Dissociation Buffer (GIBCO). mPGCLC were dissociated with TripLE Express (GIBCO). After the dissociation, cells were incubated with fluorophore-conjugated antibodies in rat serum on ice for 20 min. Cells were washed once with wash buffer and analyzed in HANK's buffer supplemented with 1% BSA. Antibodies are listed in the Key Resources Table.

### RT-qPCR

Total RNAs were purified by Reliaprep RNA miniprep kit (Promega). cDNAs were prepared by GoScript reverse transcription system (Promega). PCR was performed by Taqman Gene Expression Master Mix (Thermo Fisher Scientific) with Taqman (Thermo Fisher Scientific) or Universal Probe Library (Roche) probes. Probes and primer information are listed in Table S3.

### Immunofluorescence analysis

Cells were fixed on plates in 4% PFA for 15 minutes at RT. Cell were blocked with 5% skimmed milk or BSA/PBS 0.1% TritonX. Primary and secondary antibodies were incubated for 1 hour at RT or overnight at 4°C. Antibodies used were listed in Key Resources Table. Cells were imaged by LeicaDMI4000. PGCLCs and embryo sections were imaged by Leica SP5.

### FISH for Xist

FS cells were plated on Fn coated glass slide (Roboz Surgical instrument). The fluorescent conjugated RNA probe was purchased from Stellaris (Biosearch Technologies). *Xist* FSIH was performed as described previously (Sousa et al., 2018). Nuclear was stained with Dapi and imaged by Eclipse Ti Spinning Disk confocal microscope (Nikon).

### Metaphase chromosome analysis

FS Cells were treated with KaryoMAX colcemid (GIBCO) and cultured further 2.5 hours. Cells were washed with PBS and harvested by Accutase and collected in wash buffer. After centrifuge, cells were resuspended in 5 mL of pre-warmed 0.075M KCl and incubated for 15 minutes at RT. Freshly prepared ice cold fixative solution (methanol: glacial acetic acid (3:1)) (100 μl) were added into the suspension and centrifuge. Cells were resuspended in 250-500 μl of fixative solution and up to 20 μl was spread onto a glass slide. DNA was counterstained with DAPI and spreads were imaged by Leica DMI4000 for counting. Karyotype analysis of embryo derived hFS cell lines were performed by Medical Genetics Service, Cytogenetics Laboratory, Cambridge University Hospitals.

### Immunoblotting

Culture plates were taken out from the incubator and placed on ice. Cells were washed with ice-cold PBS and lysed with RIPA buffer in the presence of Protease/Phosphatase inhibitor cocktail (Invitrogen). Lysed cells were rotated for 20 minutes and sonicated in Bioruptor (Diagenode). Cell lysates were cleared by centrifugation, and the supernatant was recovered. Protein concentrations were measured by the BCA method (Pierce). 25 μg of protein was loaded in each well. Blots were blocked with 5% BSA/TBS 0.1% Triton-X for 1 hour at RT and incubated overnight with primary antibodies at 4°C. Secondary antibodies were incubated for 1 hour at RT and signals were detected with ECL Select (GE Healthcare) and Odyssey Fc (Li-Cor). NaOH (0.2N) was used for stripping.

### Etv4/5 and Otx2 knock out analysis

*Etv4/5* dKO ES cell lines were established from Etv4 KO ES cells (Kalkan et al., 2019) using a CRISPR/Cas9 based method. Guide RNAs (gRNAs) were designed to excise exons 13–15 of Etv5 encoding the Ets domain. *Otx2* KO ES cell lines were established from E14tg2a ES cells. gRNAs were designed to excise Exon 3 encoding the homeobox. gRNAs were cloned into pCML32. Targeted ES cell clones were picked and genotyped by genomic PCR. Oct4 and Otx2 KO in FS cells were performed by co-transfected with one gRNA expression plasmid (pCML32, Oct4-1, Otx2-1 in Table S3, puromycin resistance, *piggyBac* vector) with Cas9 expressing plasmid (G418 resistance, *piggybac* vector) and PBase expressing plasmid by TransIT LT1 (Mirus). Transfected cells were selected with 1 μg/ml of puromycin and 250 μg/ml of G418 from 24-48 hours post-transfection. Cells were counted and re-plated for another three days to form colonies. Rock inhibitor was added for the first 24 hours after replating. Alkaline phosphatase staining was performed following manufacture's instruction (Sigma-Aldrich). gRNA sequences, genotyping primers and the amplicon sizes of each genotypes are listed in Table S3.

### RNA-sequencing

For the bulk RNA-sequencing experiment, cells were lysed in Trizol (Thermo Fisher Scientific) and total RNAs were prepared using the PureLink RNA Mini Kit (Thermo Fisher Scientific). Ribosomal RNAs were removed by Ribo-Zero rRNA Removal Kit (Illumina) and libraries were constructed using the NEXTflex Rapid Directional RNA-seq Kit (Bioo Scientific). For the low-input RNA-sequencing experiment, RNA was isolated from cells and epiblasts with the PicoPure RNA Isolation kit (Thermo Fisher Scientific) and libraries were constructed using the SMARTerR Stranded Total RNA-Seq Kit v2- Pico InputMammalian (Takara Clontech). 1,000 FS cells and isolated entire single epiblasts from E5.0, E5.5, E6.0 embryos were used per sample.

### ATAC-seq

50,000 cells were collected and washed with ice-cold PBS once then lysed in lysis buffer (10 mM Tris-HCl, pH 7.4, 10 mM NaCl, 3 mM $MgCl_2$, 0.1% IGEPAL). The nuclear pellets were collected and Tn5 tagmentation and library construction performed using the Illumina Nextera kit (FC-121-1030). DNA was purified with AMPure XP beads (Beckman Coulter).

### ChIP-seq

Chromatin immunoprecipitation (ChIP) was performed as described (Kalkan et al., 2019). Briefly, chromatin was cross-linked with 1% formaldehyde for 10 minutes at RT and quenched with 125 mM Glycine for 5 minutes at RT with rotation. After cell pellets were lysed,

sonication was performed for 16 cycles on High setting, 30sec ON/30 s OFF cycle by Bioruptor (Diagenode), 2x10$^7$ cells per 300 µl in Bioruptor tube. 10% inputs were collected for the later library construction. Chromatin was immunoprecipitated with 2 µg of each antibodies and 20 µl of Protein G Dynabeads (Invitrogen) were used against 3x10$^6$ cells. After the washes, DNA was eluted and each samples were treated with 2.5 µg/ml RNase A at 37°C for 30 minutes followed by 87.5 µg/ml Proteinase K at 55°C for 1 hour. DNA was purified with PCR clean-up kit (QIAGEN). Libraries were prepared by NEXTflex Rapid DNA-Seq Kit 2.0 bundle with 96 HT barcodes (ParkinElmer).

### Single-cell RNA-seq

Cells were directly sorted into each well of 96-well plate filled with 2.3 µl of lysis buffer (1 unit/µl of SUPERaseIN RNase inhibitor (Invitrogen), 0.2% Triton X) by BD FACSAria Fusion (BD Biosciences). Libraries were prepared using the Smart-seq2 protocol (Illumina) (Picelli et al., 2014).

### Chimeras

#### *FS cell chimeras*

FS cells were pre-treated with 10 µM Rock inhibitor for 1 hour before harvesting. Around 10 singly dissociated cells were injected into each blastocyst stage embryo. Embryos are either transferred into pseudo-pregnant mice or cultured *in vitro* for another 24 hours in N2B27. E9.5 mid-gestation stage embryos and juvenile mouse tissues were imaged by Leica stereo microscope. For sectioning, embryos and E12.5 gonads were replaced with 20% sucrose/PBS overnight at 4°C after the fixation then embedded in OCT compound and sectioned at 8 µm thickness. Sections were imaged by Zeiss apotome microscope or Leica SP5 confocal microscope.

#### *Epiblast chimeras*

Homozygous mTmG mice were crossed with CD1 mice to obtain embryos. E5.5, 6.0-6.25 and E6.5 embryos were dissected from decidua and separated into embryonic and extraembryonic halves. Extraembryonic endoderm layers were removed using a mouth-controlled pulled Pasteur pipette. Isolated epiblasts were treated with Accutase at room temperature and washed with M2 medium in the presence of 10 µM Rock inhibitor. Ten dissociated cells were injected per E3.5 blastocyst stage embryo of strain C57BL/6. Microinjection was performed in M2 medium containing Rock inhibitor. For sectioning, embryos were embedded in OCT compound and sectioned at 10µm thickness. Sections were stained with anti-RFP antibody and imaged using a Leica DMI4000.

## QUANTIFICATION AND STATISTICAL ANALYSIS

### Bulk RNA-seq analysis

Low-quality RNA-seq reads and adaptor sequences were removed using *Trim Galore!*. Reads were aligned to the mouse (GRCm38/mm10) and human (GRCh38/hg38) reference genomes using *TopHat2* with parameters " –read-mismatch 2 –max-multihits 1 –b2-sensitive" considering uniquely mapping reads only. Gene counts were obtained using *featureCounts* using ENSEMBL (release 89) gene annotations. Normalization and differential expression analyses were performed using the R/Bioconductor *DESeq2* package. Normalized counts were transformed into log2 fragments per million (FPKM). Genes with log2 fold change > 1.6 and adjusted p value < 0.05 were considered differentially expressed. Differentially expressed gene clusters for human cells were identified by k-means clustering of the first five principal components using the R '*kmeans*' function. The distance plot was calculated using Euclidean distance between samples based on log2 normalized counts of expression values. Heatmaps were generated using the R '*pheatmap*" function.

For transposable elements (Tes), reads were aligned to the human (GRCh38/hg38) reference genome using *bowtie* with parameters "-a –best –strata -m 1 -v 2," retaining uniquely mapping reads only in order to identify the genomic origin of TE transcription. Read counts on Tes were obtained using *featureCounts* on UCSC RepeatMasker-annotated regions. Normalization and differential expression analyses between cell types of identical genotype were performed with the R/Bioconductor *DESeq* package. Tes with an expression of at least log2-normalized counts > 3.5 in any cell type, a log2 fold change > 2 and an adjusted p value < 0.05 were considered differentially expressed.

### Published RNA-seq data comparison analysis

Mouse single cell RNA-seq data was downloaded from Nakamura et al. (2016) (GEO: GSE74767). Human naive and conventional PSC transcriptome data were downloaded from SRA: SRP104789, ENA:E-MTAB-5114, ENA:E-MTAB-5674, GEO:GSE123005. The data was processed using the same methods as described above, except that genes with zero counts were removed from the single cell RNA-seq data matrix before further processing by DESeq2. The matrix of log2 fragment per millions for the *Macaca fascicularis* was obtained from GEO: GSE74767 (Nakamura et al., 2016). The Human single cell RNA-seq FPKM ummarized counts matrix was downloaded from GEO: GSE136447 (Xiang et al., 2019).

### PCA plots

Principal component analyses (PCA) were performed using the R '*prcomp*' function based on log2-transformed Z-score expression values. To compare mouse and human bulk RNA-seq with mouse and macaque single cell RNA-seq, the principal components of the single cell RNA-seq data were calculated, with the bulk RNA-seq data projected onto this PCA space using the R '*predict'* function. These PCAs were computed using all expressed genes or with genes differentially expressed between the formative and primed lines

in order to narrow down genes important for developmental progression. To compare human bulk RNA-seq with human single cell RNA-seq data, Log2 transformed counts were used. Using the most variable genes across the single cell stages, a PCA of the bulk samples was computed and the single cells were projected using the R '*predict*' function.

### scRNA-seq analysis

Raw files were quality controlled using FastQC v0.11.3 and results ummarized with MultiQC, with checks including distributions of nucleotide content and sequencing depth. Reads were aligned to the *M.musculus* GRCm38.p6 reference genome with Ensembl v98 annotations using STAR v2.7.3a (–outSAMtype BAM SortedByCoordinate). Protein-coding gene quantification was done using Subread featureCounts v2.0.0 with Ensembl v98 annotations; only uniquely mapped reads were used. Cells with fewer than 3M reads were removed from further analysis, leaving 326 cells that passed the threshold. Raw expression levels were normalized using sctransform (Hafemeister and Satija, 2019), and the PCA created using the 2000 most abundant genes across the data. Jaccard similarity indices were calculated on the 2000 most abundant genes per cell, with similarities calculated between all cells of the same type.

### GO-terms

Gene ontology (GO) term enrichment analyses were performed using the *David* tool.

### ATAC-seq

Reads were quality-trimmed using *Trim Galore!*, and reads shorter than 15 nt were discarded. Reads were aligned to the mouse reference genome (GRCm38/mm10) using *bowtie* with parameters "-m1 -v1 –best –strata -X 2000 –trim3 1." Duplicates were removed using *Picard tools*. Reads longer than one nucleosome length (146 nt) were discarded, and an offset of 4 nts was introduced. Peaks were called with *MACS2* and parameters "–nomodel –shift −55 –extsize 110 –broad -g mm –broad-cutoff 0.1." Bigwig files for visualization on the UCSC Genome browser were generated using *deeptools bamcoverage* with parameters "–binSize 10 and –normalizeUsing RPKM." ATAC peaks specific to each cell type were identified using *edgeR* within the R/Bioconductor *DiffBind* package using the option "bNot = T" to allow for contrasts between each cell type against all others. Significant peaks were determined using a log2 fold change of > 1 and FDR < 0.05. Heatmaps of ATAC-seq peaks were generated with *deeptools plotHeatmap*. DNA motif enrichment analyses for cell type-specific ATAC-seq peaks was performed using *HOMER*.

### BS-seq

Whole genome BS-seq data was obtained from Zylicz et al., 2015 (GEO: GSE70355**)**. BS-seq reads were aligned to the mouse reference genome (GRCm38/mm10) and deduplicated using *Bismark*. *MethPipe* was used calculate methylation levels at each CpG, and only CpGs with at least 5X read coverage were retained for further analyses. Methylation levels were averaged using a 250nt-sliding window to generate bigwig files.

### ChIP-seq

Raw files were quality controlled using FastQC v0.11.3 and results summarized with MultiQC, with checks including distributions of nucleotide content, sequencing depth and adaptor contamination. Reads were aligned to the *M.musculus* GRCm38.p6 reference genome using bwa mem v0.7.10-r789 (default parameters); the MT, X, Y chromosomes and scaffolds were excluded from the resulting BAM files. Genome browser tracks for the UCSC genome browser were created with deepTools bamCoverage v3.3.1 (—binSize 30). Averaged genome browser tracks for ChIP profile visualization were created as follows: first the tracks were generated with bamCoverage (—binSize 5 –normalizeUsing RPKM), then the output was averaged using wiggletools v1.2.1 (Zerbino et al., 2014). Profiles of the ChIP tracks on the ATAC peaks were created using deepTools computeMatrix (reference-point–binSize 5 -b 4000 -a 4000–referencePoint center) and plotProfile (default parameters). To identify bivalent promoters, peak regions were called with macs2 v2.2.6 (-f BAMPE -q 0.05), only peaks with signalValue > 5 were considered for downstream analysis. Peak regions were intersected per condition and across histone marks using bedops v2.4.38. HOMER v4.10 was used to calculate distance between peaks and transcription start sites (mm10 -size 3000); peaks within 3kb of a TSS were considered as promoter peaks.

Cell Stem Cell, Volume *28*

## Supplemental Information

# Capture of Mouse and Human Stem Cells with Features of Formative Pluripotency

**Masaki Kinoshita, Michael Barber, William Mansfield, Yingzhi Cui, Daniel Spindlow, Giuliano Giuseppe Stirparo, Sabine Dietmann, Jennifer Nichols, and Austin Smith**

# Figure S1 (Related to Figure 1)

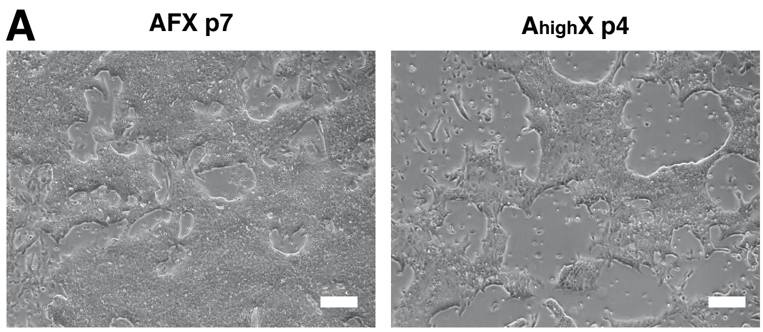


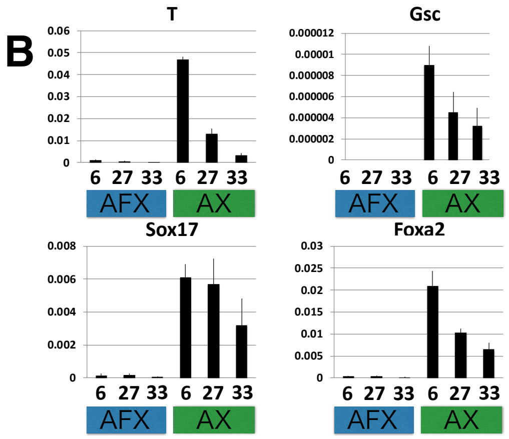


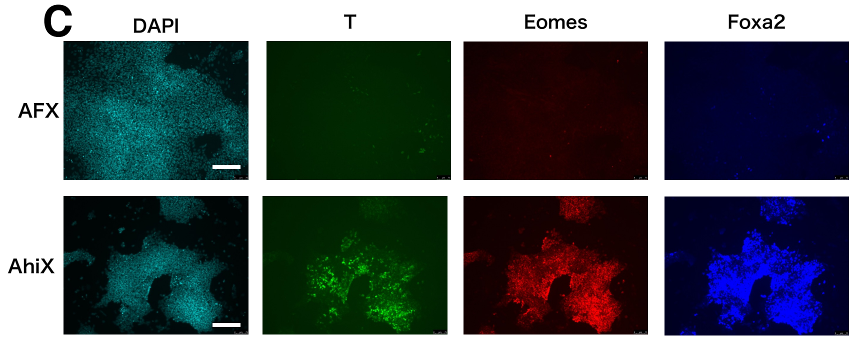


D

| Concentration of activin | No of E5.5 embryos | No of established lines (> p10) | Success rate |
|---|---|---|---|
| 1ng | 8 | 0 | 0.0% |
| 1.5ng | 8 | 1 | 12.5% |
| 2ng | 11 | 3 | 27.3% |
| 2.5ng | 4 | 0 | 0.0% |
| 3ng | 11 | 4 | 36.4% |

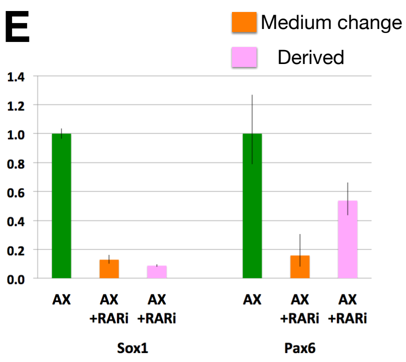


F

| | Number of E5.5 embryos | Lines established | Efficiency |
|---|---|---|---|
| AloX | 8 | 4 | 50% |
| AloX +RARi | 4 | 4 | 100% |

G

| Line | Percentage of diploid (=40) cells | Passage number |
|---|---|---|
| 5a6 | 75.0 % (24/32) | 19 |
| NBRA3.2 | 87.1 % (27/31) | 22 |
| 5ar1 | 64.0 % (16/25) | 21 |
| 5ar2 | 83.3% (20/24) | 26 |

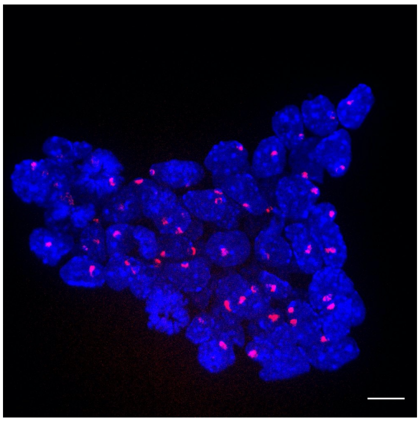


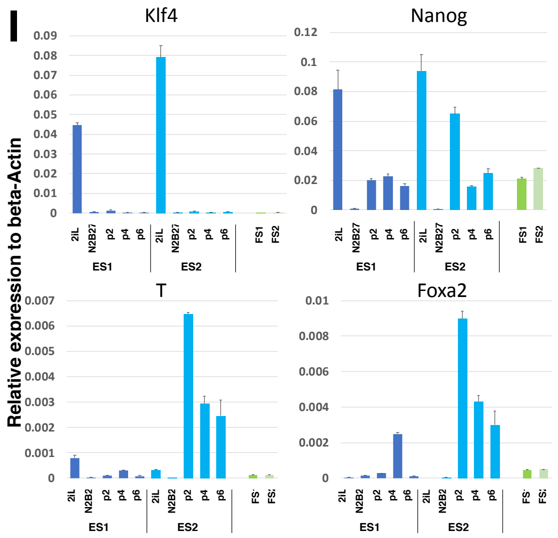


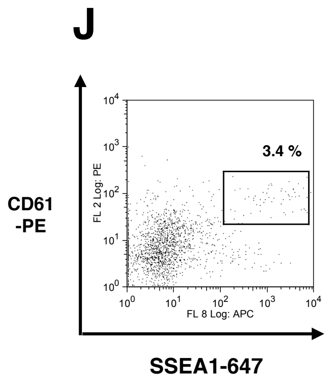


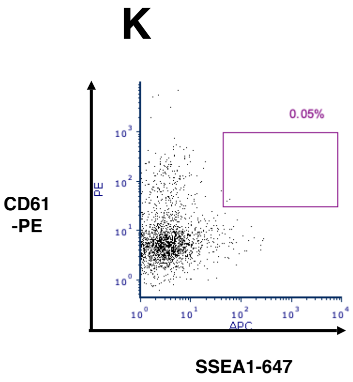


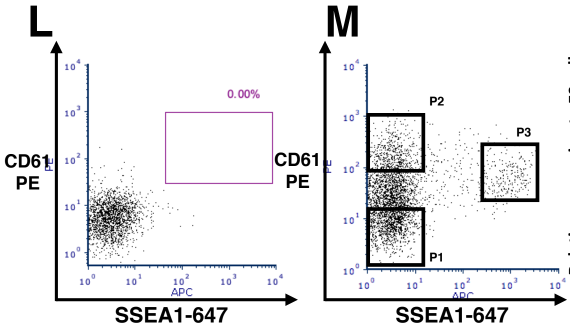


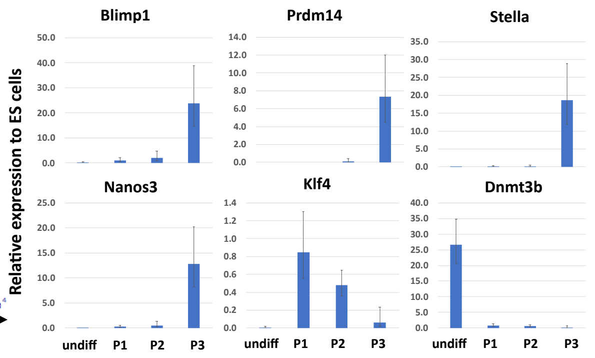


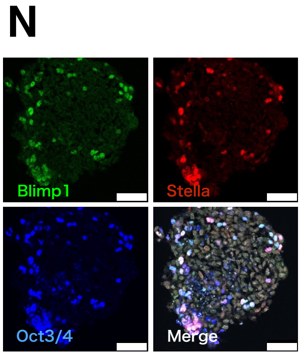

# Figure S2 (Related to Figure 2)

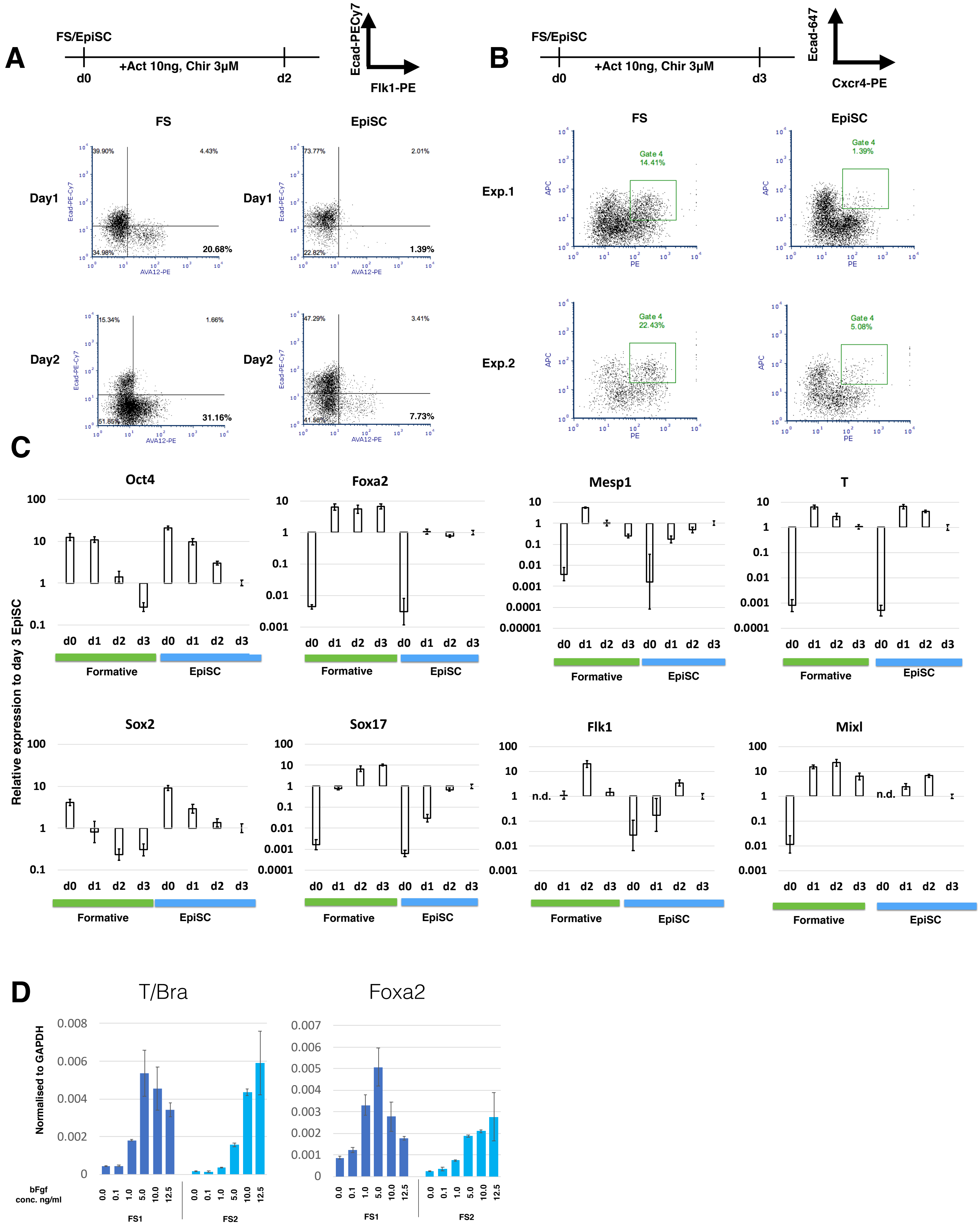

# Figure S3 (Related to Figure 3)

A B C

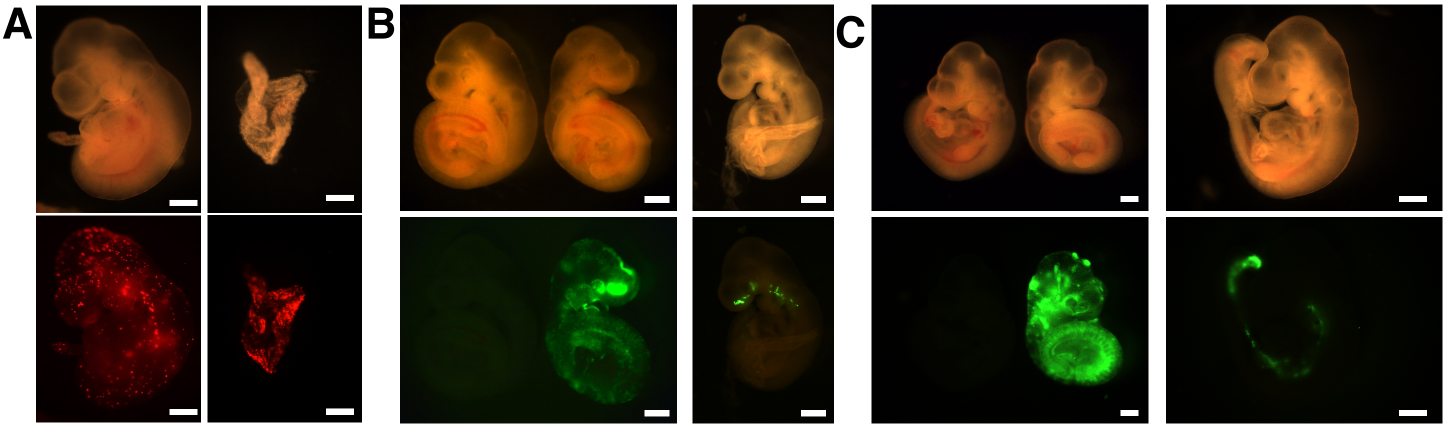

D

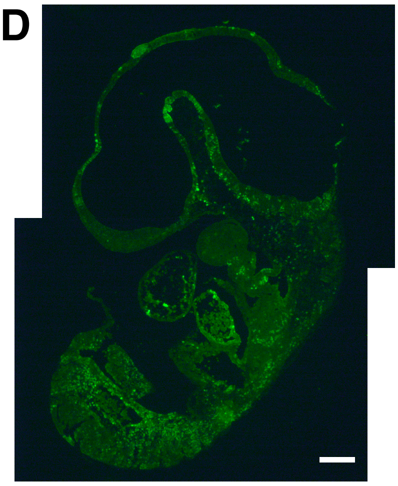

E

| Line | Embryos recovered | Widespread Contribution | Localised contribution | Yolk sac contribution | Chimaera rate |
|---|---|---|---|---|---|
| NBRA3.2 | 48 | 8 | 11 | 1* | 39.6% |
| 5a6 | 20 | 1 | 2 | 1 | 15.0% |
| 5ar1 | 17 | 1 | 3 | Not Investigated | 23.5% |

F

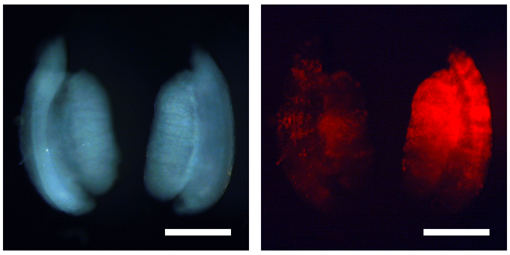

G

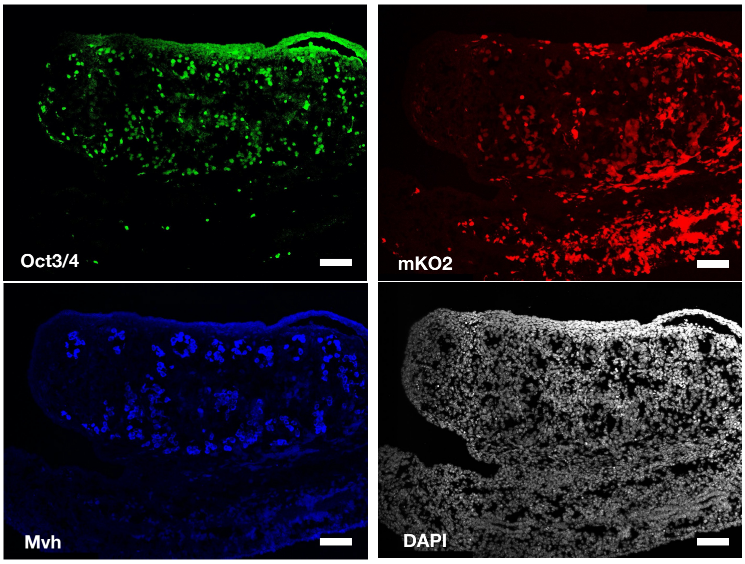


H E6.0 Epiblast Chimaeras

I E5.5 Epiblast Chimaera

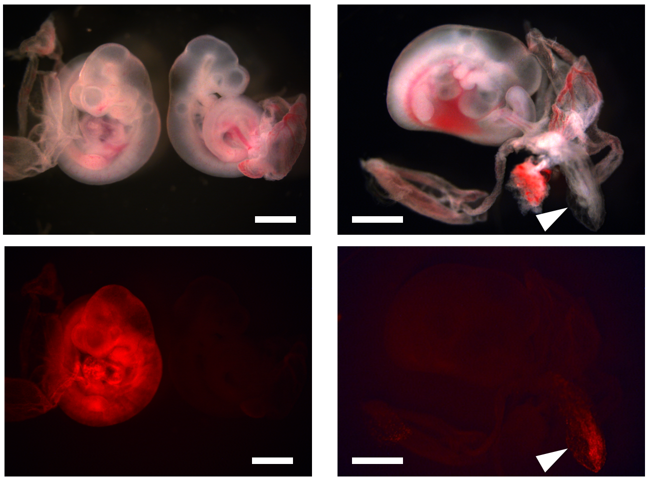

J E5.5 Epiblast Chimaera

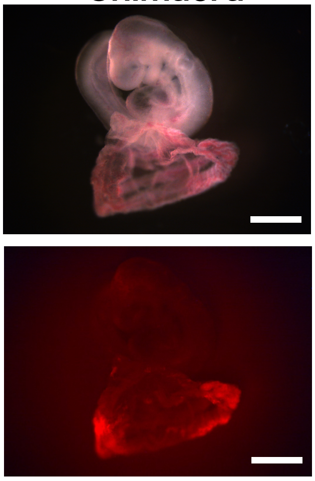

K

K'

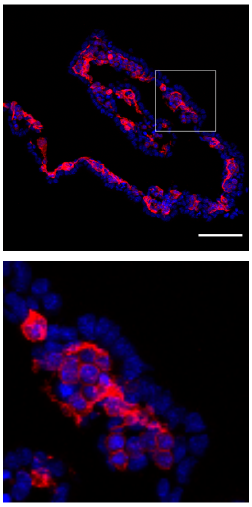

L

| Donor stage | Embryos recovered | Widespread Contribution | Localised contribution | contribution yolk sac and/or amnion | Chimaera rate |
|---|---|---|---|---|---|
| E5.5 | 44 | 2 | 5 | 7 | 16.0% |
| E6.0 | 47 | 1 | 3 | 4 | 8.5% |
| E6.5 | 29 | 0 | 0 | 0 | 0.0% |

# Figure S4 (Related to Figure 4)

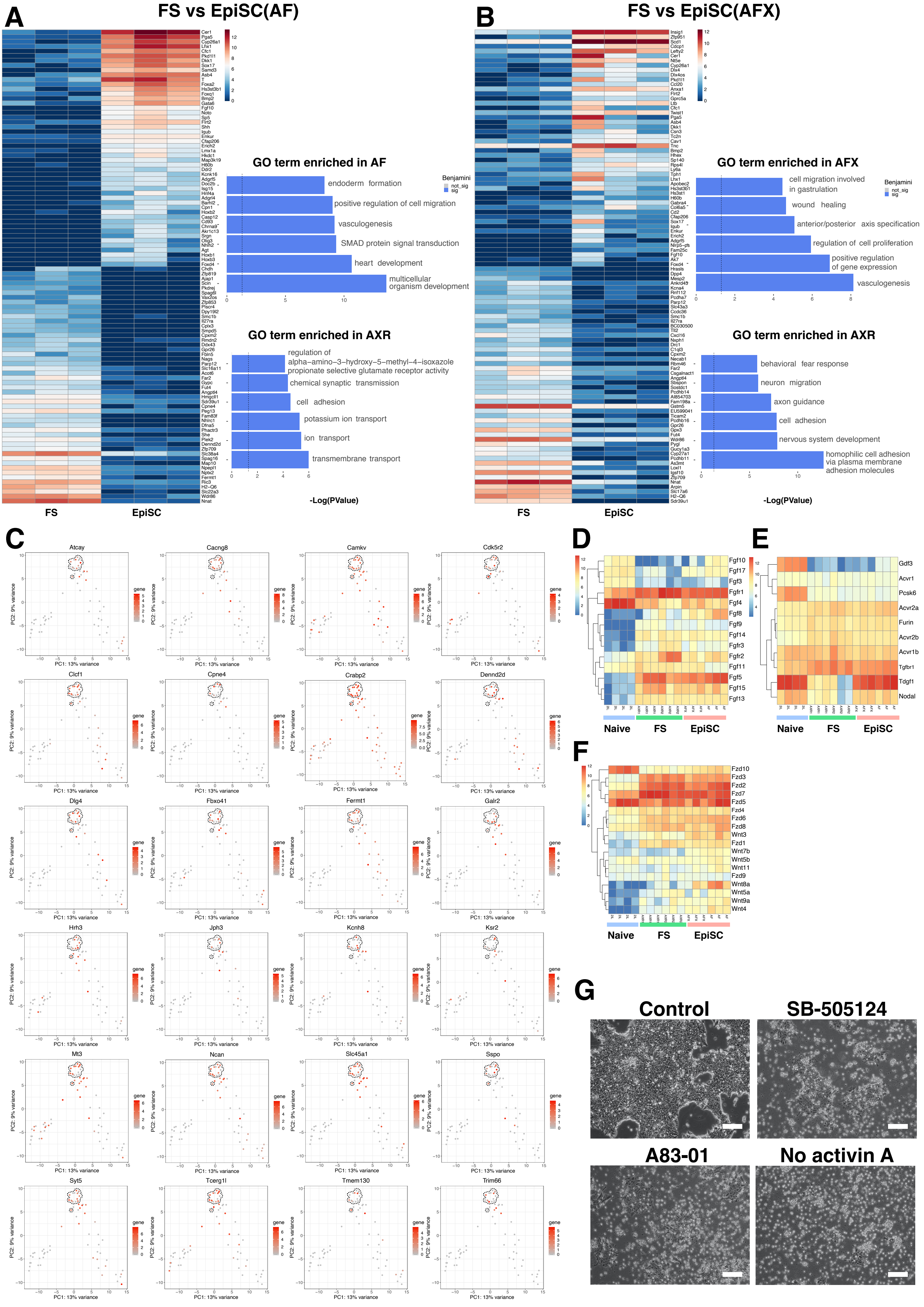

# Figure S5 (Related to Figure 5)

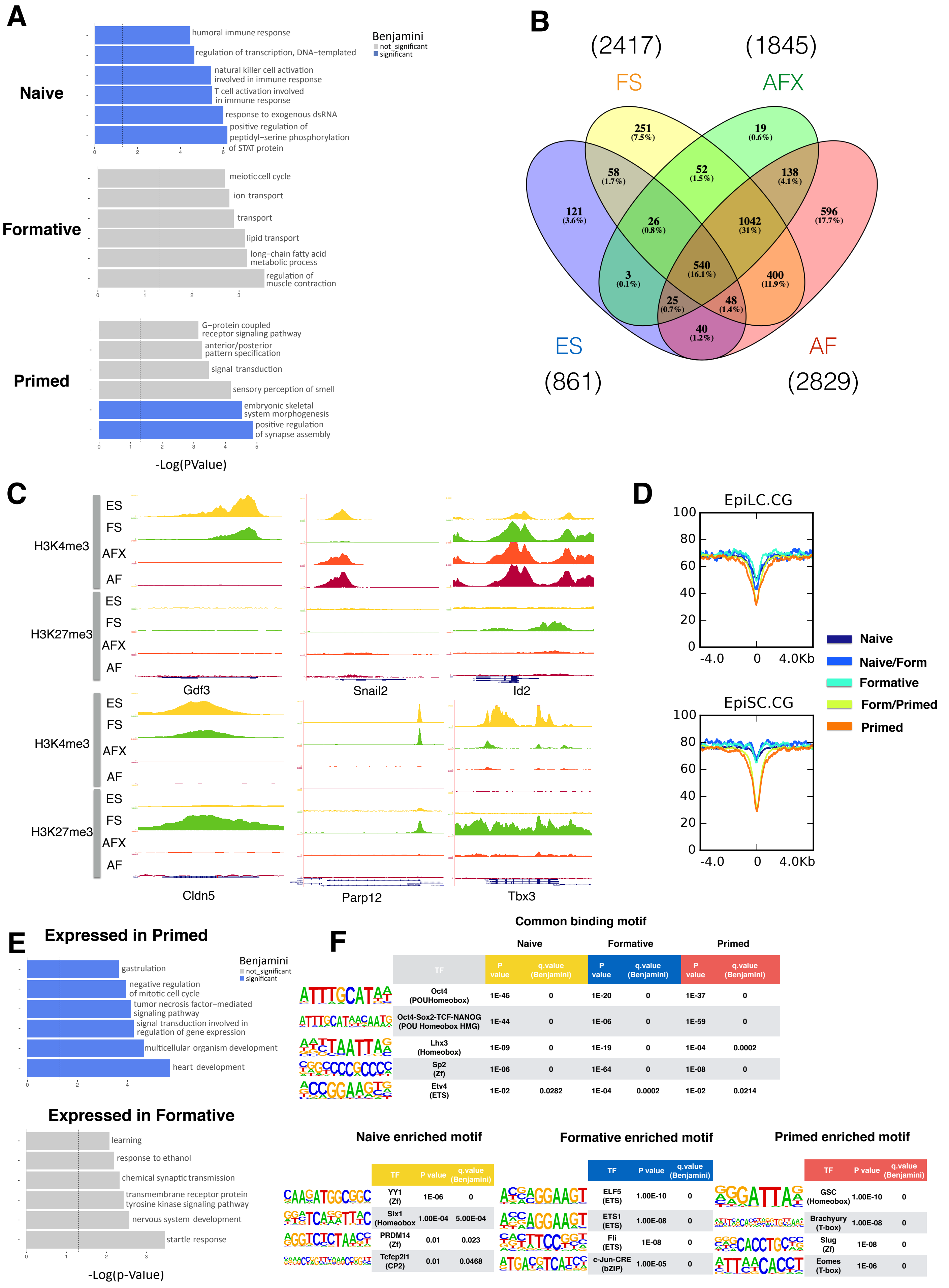


**Common binding motif**

| TF | Naive P value | Naive q.value (Benjamini) | Formative P value | Formative q.value (Benjamini) | Primed P value | Primed q.value (Benjamini) |
|---|---|---|---|---|---|---|
| Oct4 (POUHomeobox) | 1E-46 | 0 | 1E-20 | 0 | 1E-37 | 0 |
| Oct4-Sox2-TCF-NANOG (POU Homeobox HMG) | 1E-44 | 0 | 1E-06 | 0 | 1E-59 | 0 |
| Lhx3 (Homeobox) | 1E-09 | 0 | 1E-19 | 0 | 1E-04 | 0.0002 |
| Sp2 (Zf) | 1E-06 | 0 | 1E-64 | 0 | 1E-08 | 0 |
| Etv4 (ETS) | 1E-02 | 0.0282 | 1E-04 | 0.0002 | 1E-02 | 0.0214 |

**Naive enriched motif**

| TF | P value | q.value (Benjamini) |
|---|---|---|
| YY1 (Zf) | 1E-06 | 0 |
| Six1 (Homeobox) | 1.00E-04 | 5.00E-04 |
| PRDM14 (Zf) | 0.01 | 0.023 |
| Tcfcp2l1 (CP2) | 0.01 | 0.0468 |

**Formative enriched motif**

| TF | P value | q.value (Benjamini) |
|---|---|---|
| ELF5 (ETS) | 1.00E-10 | 0 |
| ETS1 (ETS) | 1.00E-08 | 0 |
| Fli (ETS) | 1E-08 | 0 |
| c-Jun-CRE (bZIP) | 1.00E-05 | 0 |

**Primed enriched motif**

| TF | P value | q.value (Benjamini) |
|---|---|---|
| GSC (Homeobox) | 1.00E-10 | 0 |
| Brachyury (T-box) | 1.00E-08 | 0 |
| Slug (Zf) | 1E-08 | 0 |
| Eomes (T-box) | 1E-06 | 0 |

## Figure S6 (Related to Figure 6)

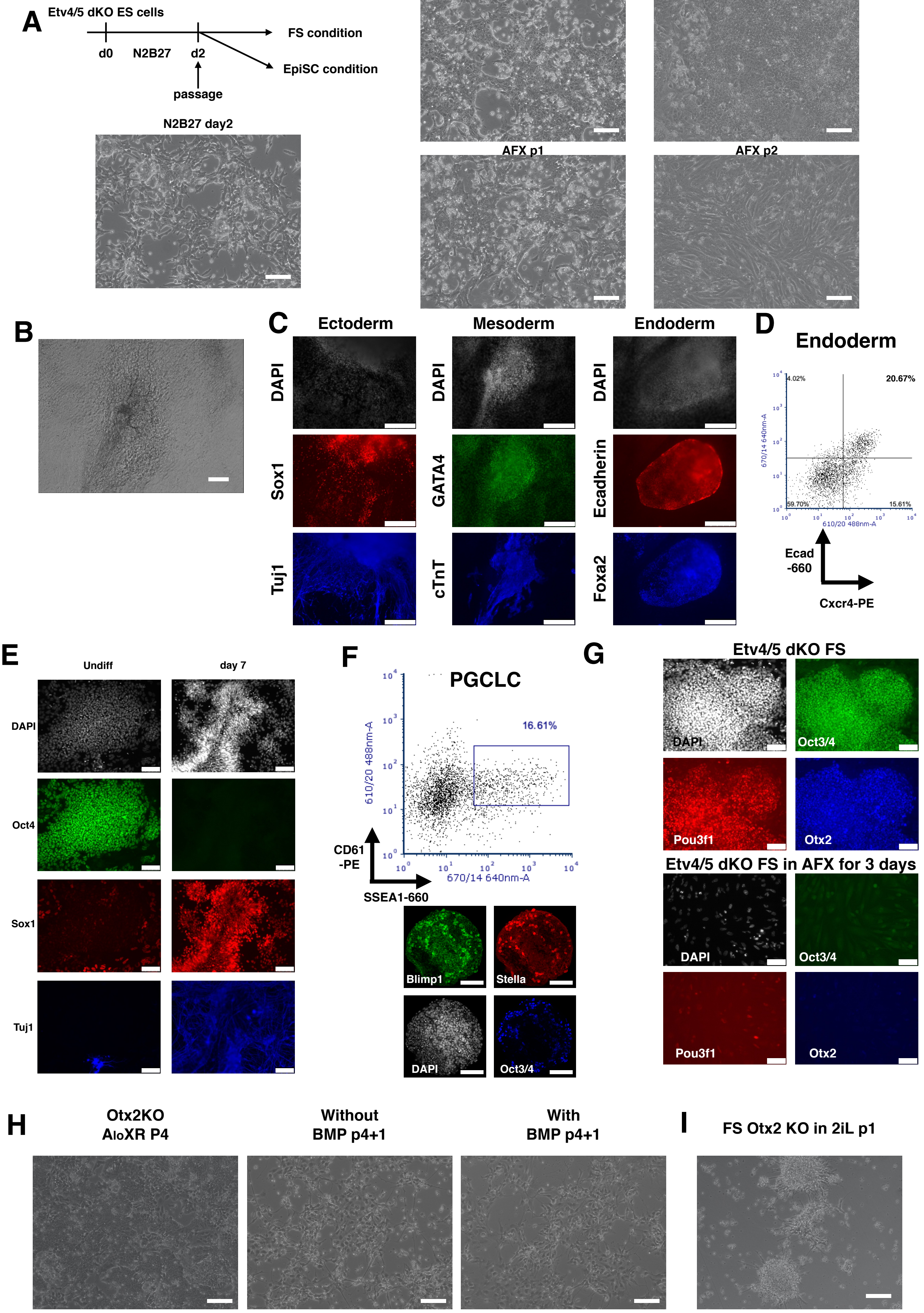

# Figure S7 (Related to Figure 7)

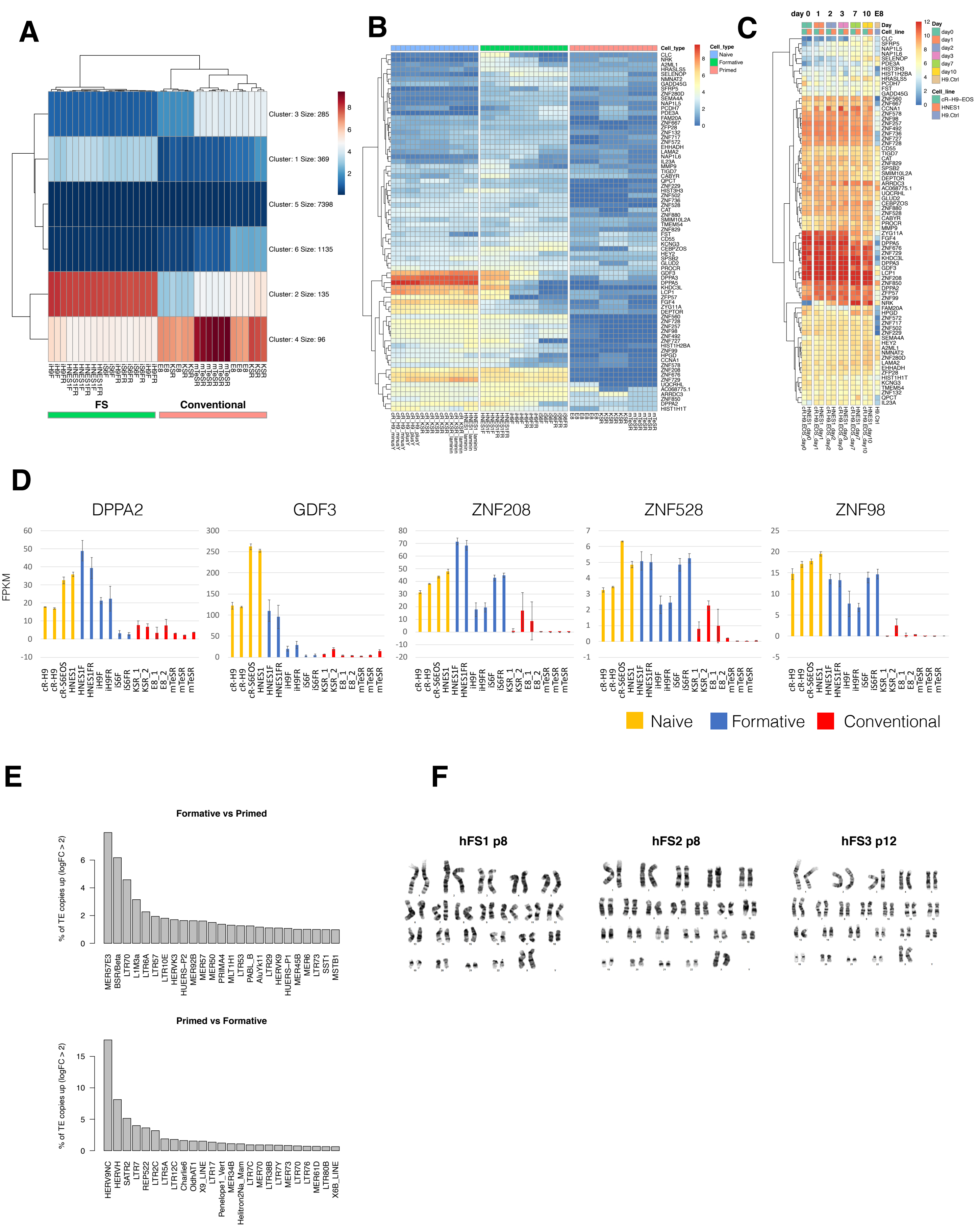

## Supplemental Figure Legends

**Figure S1. Derivation of stem cell lines from formative epiblast, Related to Figure 1**
(A) Bright field image of E5.5 epiblast derived AFX and $A_{hi}X$ cultures. Scale bars, 200µm. (B) Gene expression analysis after FGF withdrawal. Three AFX cell lines (6, 27 and 33) were passaged without FGF and analysed by RT-qPCR. Error bars are S.D. from technical triplicates. (C) Immunostained images of early lineage marker expression in AFX and $A_{hi}X$ cells. Scale bars, 100µm. (D) Summary of derivation efficiency from E5.5 epiblasts in different concentrations of activin A. (E) RT-qPCR analysis of RAR inhibitor treated cells. $A_{lo}XR$ samples established in $A_{lo}X$ and transferred to $A_{lo}XR$ are in orange and a line derived in $A_{lo}XR$ in pink. Error bars, S.D. from technical triplicates. (F) Derivation efficiency in the absence of presence of RAR inhibitor. (G) Percentages of diploid cells for 4 FS cell lines. (H) Maximum projection of Z-stack slices of *Xist* RNA FISH images (red) in female FS cells. Nuclei were stained with DAPI (blue). Scale bar, 10µm. (I) Gene expression analysis by RT-qPCR during ES cell to FS cell conversion. Gene expression is relative to beta-actin. Error bars are S.D. from two technical replicates. (J) Flow cytometry analysis of day 4 PGCLC induction from $A_{lo}X$ FS cells. (K) Analysis of day 4 PGCLC induction from AFX EpiSCs. (L) Analysis of day 4 PGCLC induction from AFX EpiSCs adapted to culture in $A_{lo}XR$. (M) $A_{lo}XR$ cells sorted for SSEA1 and CD61 co-expression on day 6 of PGCLC induction (left) and subject to RT-qPCR analysis (right). Relative expression level to 2iL ES cells (=1) normalized to Tbp. Error bars represent S.D. from technical triplicates. (N) Immunostaining of $A_{lo}X$ cell-derived PGCLC. Scale bars, 50µm.

**Figure S2. Lineage potency of FS cells and responsiveness to differentiation cues, Related to Figure 2**
(A) Flow cytometry profiles of $Flk1^{+}Ecad^{-}$ mesodermal fraction of differentiated FS cells and EpiSCs at day 1 and day 2. (B) $Cxcr4^{+}Ecad^{+}$ endoderm fraction at day 3. Two experiments are shown. (C) RT-qPCR analysis after activin A and CH treatment for 3 days. AFX EpiSC samples at day 3 were set as 1, normalisation to 36B4 (Rplp0). Error bars represent SD from technical triplicates. n.d. not detected. (D) RT-qPCR analysis of T and Foxa2 expression 24 hours after indicated doses of Fgf2 were added into $A_{lo}XR$ culture. Error bars represent S.D. from technical duplicates.

**Figure S3. Blastocyst chimaera contribution by FS cells and formative epiblast, Related to Figure 3**
(A) Left, low contribution E9.5 chimaera produced from mKO2-labelled NBRA3.2 FS cells. Right, yolk sac contribution in one of the chimaeras in Fig. 3A. Scale bars, 500µm. (B) E9.5 chimaeras from GFP-labelled 5a6 FS cells. Contributions were widespread (left) or localised (right). Scale bars, 500µm. (C) E9.5 chimaeras from GFP-labelled 5ar1 FS cells. Scale bars, 500µm. (D) Sagittal section of embryo from C, left panel, with widespread contribution of GFP positive cells. Scale bar, 200µm. (E) Summary of FS cell chimaeras examined at E9.5. *Not all yolk sacs from chimaeric embryos were examined. (F) E12.5 chimaeric gonads generated from mKO2-labelled FS cells. Scale bars, 500µm. (G) Section of gonad from (F) stained with anti-Oct4 and anti-Mvh antibodies. Nuclei were stained with DAPI. (H-J). E9.5 chimaeras with contribution from E5.5 and E6.0 donor epiblast. Contributions were detected in the embryo proper and yolk sac (H), amnion (arrowhead) (I), yolk sac (J). Scale bars, 1mm. (K) Yolk sac section showing membrane-tdTomato positive cells in the inner layer of extraembryonic mesoderm. Nuclei were stained with DAPI (blue). Scale bar, 100µm. Magnified image from boxed region is shown as (K’). (L) Summary of post-implantation epiblast chimaeras.

**Figure S4. Whole transcriptome analysis and nodal/activin pathway activity, Related to Figure 4**

(A) Heatmap for top 50 differentially expressed genes (DEG) between FS cells and EpiSCs (AF). GO terms are shown (Benjamini value<0.05) for analysis of 200 DEG. (B) Heatmap for top 50 DEG between FS cells and EpiSCs (AFX). GO term analysis as in A (Benjamini value<0.05). (C) Example embryo gene expression profiles of FS cell enriched genes identified in Fig. 4B. E5.5 epiblast cells are highlighted by the dashed circle. (D) Heatmap of expression of Fgfs and Fgfrs. (E) Heatmap of Nodal pathway gene expression. (F) Heatmap of expression of Wnts and Fzd receptors. Colour scale in (D-F) is log2(normalised counts +1) from RNA-seq. (G) Cell morphologies after two days in indicated culture conditions: $A_{lo}XR$; 1μM A83-01 in $A_{lo}XR$; 5μM SB505124 in $A_{lo}XR$; without activin A in 2μM XAV939 and 1μM BMS493. Scale bars,100μm.

**Figure S5. Chromatin landscape analysis, Related to Figure 5**

(A) GO term enrichment for genes proximal to phase specific ATAC-seq sites. Bars in blue have a significant Benjamini value<0.05. (B) Enumeration of bivalent domains in each cell type. (C) Genome browser screenshots of differential histone modifications. Lower three examples show formative specific bivalency. (D) Methylation at ATAC peaks in EpiLCs and EpiSCs (original data from Zylicz et al., 2015). (E) Related to Fig. 5G. GO term analysis performed against significantly expressed genes in EpiSCs or FS cells. Bars in blue have a significant Benjamini value<0.05. (F) Transcription factor binding motifs and P-values enriched in phase specific ATAC sites.

**Figure S6. Differential requirements for *Etv4/5* and *Otx2*, Related to Figure 6**

(A) Schematic of ES cell differentiation to FS cells or EpiSCs and morphologies of *Etv4/5*dKO cells at day 2, P1 and P2. (B) Bright field image of contracting *Etv4/5*dKO differentiated cells. (C) Immunostaining of *Etv4/5*dKO FS cell EB outgrowth. Neuroectoderm stained with Sox1 (red) and Tuj1 (Blue), mesoderm with Gata4 (Green) and cTnT (blue), and endoderm with Ecadherin (red) and Foxa2 (Blue). DAPI stainings were shown in white. (D) Flow cytometry plot of endoderm differentiated *Etv4/5*dKO FS cells. (E) Immunostaining for Oct3/4 (green), Sox1 (red) and Tuj1 (Blue) after neural differentiation of *Etv4/5*dKO FS cells. (F) PGCLC induction from *Etv4/5*dKO FS cells analysed by flow cytometry for SSEA1-660 and CD61-PE and by immunostaining for Blimp1 (green), Stella (red) and Oct4 (blue). (G) Immunostaining of *Etv4/5*dKO FS cells in $A_{lo}XR$ and after transfer to EpiSC culture (AFX) for three days. (H) *Otx2* KO cells passaged in $A_{lo}XR$ with or without BMP. (I) Bright field image of *Otx2* KO FS cells re-plated in 2iL. Scale bars in (A), (B), (F), (H), (I) 100μm, (C) 250μm and (E), (G) 75μm.

**Figure S7. Human FS-like cells established from naïve ES cells and embryos, related to Figure 7**

(A) K-mean clustering of differential gene expression between human FS-like cells and conventional PSCs. (B) Gene expression heatmap for cluster 1 protein coding genes. (C) Expression heatmap of cluster 1 protein coding genes during naïve cell capacitation (data from Rostovskaya et al 2019). (D) Related to Figure 7I, FPKM values for additional selected naïve-formative specific genes. (E) Bar charts of differentially expressed TE families between formative and conventional hPSCs. (F) G-banded chromosomes from three independent human embryo derived FS-like cell lines.
